# Systematics, conservation and morphology of the spider genus *Tayshaneta* (Araneae, Leptonetidae) in Central Texas Caves

**DOI:** 10.3897/zookeys.167.1833

**Published:** 2012-01-23

**Authors:** Joel Ledford, Pierre Paquin, James Cokendolpher, Josh Campbell, Charles Griswold

**Affiliations:** 1 California Academy of Sciences, Department of Entomology, San Francisco, CA 94118, USA; 2 Environmental Science, Policy and Management, Division of Organisms and Environment, University of California, Berkeley, Berkeley, CA 94720, USA; 3 SWCA Environmental Consultants, Austin, Texas, 78749, USA; 4 Museum of Texas Tech University, Lubbock, Texas, 79409, USA; 5 High Point University, High Point, North Carolina, 27262, USA

**Keywords:** Spiders, Haplogynae, Leptonetidae, *Neoleptoneta*, Caves, Endangered Species, Troglobites, Edwards Aquifer, Karst Faunal Regions, Phylogenetics

## Abstract

The spider genus *Tayshaneta* is revised based on results from a three gene phylogenetic analysis (Ledford et al. 2011) and a comprehensive morphological survey using scanning electron (SEM) and compound light microscopy. The morphology and relationships within *Tayshaneta* are discussed and five species-groups are supported by phylogenetic analyses: the *anopica* group, the *coeca* group, the *myopica* group, the *microps* group and the *sandersi* group. Short branch lengths within *Tayshaneta* contrast sharply with the remaining North American genera and are viewed as evidence for a relatively recent radiation of species. Variation in troglomorphic morphology is discussed and compared to patterns found in other Texas cave invertebrates. Several species previously known as single cave endemics have wider ranges than expected, suggesting that some caves are not isolated habitats but instead form part of interconnected karst networks. Distribution maps are compared with karst faunal regions (KFR’s) in Central Texas and the implications for the conservation and recovery of *Tayshaneta* species are discussed. Ten new species are described: *Tayshaneta archambaulti* **sp. n.**, *Tayshaneta emeraldae* **sp. n.**, *Tayshaneta fawcetti* **sp. n.**, *Tayshaneta grubbsi* **sp. n.**, *Tayshaneta madla* **sp. n.**, *Tayshaneta oconnorae* **sp. n.**, *Tayshaneta sandersi* **sp. n.**, *Tayshaneta sprousei* **sp. n.**, *Tayshaneta vidrio* **sp. n.** and *Tayshaneta whitei* **sp. n.** The males for three species, *Tayshaneta anopica* (Gertsch, 1974), *Tayshaneta devia* (Gertsch, 1974) and *Tayshaneta microps* (Gertsch, 1974) are described for the first time. *Tayshaneta furtiva* (Gertsch, 1974) and *Tayshaneta uvaldea* (Gertsch, 1974) are declared *nomina dubia* as the female holotypes are not diagnosable and efforts to locate specimens at the type localities were unsuccessful. All *Tayshaneta* species are thoroughly illustrated, diagnosed and keyed. Distribution maps are also provided highlighting areas of taxonomic ambiguity in need of additional sampling.

## Introduction

*Tayshaneta* are small spiders that belong to the family Leptonetidae, a group recognized for its association with caves and similar cryptic habitats (Ledford et al. 2004). *Tayshaneta* are widely distributed in caves of the Edward’s Plateau (Fig. 3), an extensive limestone region in Central Texas that drains into the Edward’s Aquifer and serves as the primary source of water for over 2 million people. The region is famous for its endemism and includes a high proportion of endangered and threatened species, many of which are subterranean specialists and known only from single springs or caves (Culver et al. 2003). Two *Tayshaneta* species are federally listed as endangered in Central Texas, *Tayshaneta microps* (Gertsch, 1974) and *Tayshaneta myopica* (Gertsch, 1974) and most others are of conservation concern (Bender et al. 2005; U.S. Fish and Wildlife Service 1998, 2010). However, management and recovery efforts are limited by existing taxonomy which is poorly resolved and leaves the identity and distribution of *Tayshaneta* species ambiguous.

Gertsch (1974) described the majority of the North American Leptonetidae and considered twelve species as part of a closely related Texas fauna. Although he originally described these species as congeneric with European *Leptoneta*, several publications (Brignoli 1977, 1979; Platnick 1986) refuted this hypothesis and transferred the Texas fauna to the genus *Neoleptoneta* Brignoli, 1972. Two species were later added by Cokendolpher and Reddell (2001) and Cokendolpher (2004), who also provided details on their general biology. Recent phylogenetic work has shown that *Neoleptoneta* is paraphyletic and three additional genera, *Chisosea*, *Ozarkia* and *Tayshaneta*, were described (Ledford et al. 2011). *Tayshaneta* presently includes eleven species restricted to Texas caves with close relatives in the Southeast, Southern Texas and Northern Mexico (Ledford et al. 2011).

While Gertsch’s (1974) study was the first to comprehensively treat the North American fauna, the taxonomic challenges of leptonetids frustrated him (D. Ubick, pers. comm.). Most species are represented by few specimens which in addition to being relatively small (1-2mm) are also delicate and easily damaged during examination. Furthermore, the characters used to separate species are exceptionally fine and not often visible using conventional microscopy. European specialists, including Brignoli (1972, 1974), Fage (1913) and Machado (1941, 1945) relied heavily on compound light microscopy to produce detailed illustrations which Gertsch was reluctant to use. Consequently, most species remain poorly diagnosed and positive identification is only possible with topotypic material. Morphological homogeneity within female specimens is also problematic (Ledford 2004; Ledford and Griswold 2010) and although microscopy and preparation techniques have improved, leptonetid taxonomy remains dependent upon the details of male genitalia. Diagnostic features for *Tayshaneta* in particular are subtle and often require examination using scanning electron microscopy.

Recent studies on *Cicurina* spiders in Texas caves (Paquin and Hedin 2004; Paquin et al. 2008) have addressed similar problems by using molecular phylogenetic methods and fine scale geographic sampling to help resolve species limits. Although based on a single genetic locus, Paquin and Hedin (2004) clearly demonstrated that the integration of molecular data is a valuable aid to overcoming the difficulties of working with cave fauna, especially when specimens are rare or present diagnostic challenges. Studies of cave invertebrates are also underscored by conservation concerns, especially in Central Texas, where taxonomic identity can have profound socioeconomic impact. As emphasized by Paquin et al. (2008), the interaction between taxonomists, conservation biologists and development interests can be volatile and highlights the need for robust, integrative taxonomy based on multiple lines of evidence.

Several geological areas are recognized on the Edward’s Plateau, however most of the subterranean diversity is known from caves along the heavily faulted Balcones Escarpment (Fig. 3). The faulting serves to isolate regions of limestone and is likely correlated with the diversification patterns of cave invertebrates (White et al. 2009). Conservation biologists have used this fragmented geology to develop a conservation strategy based on “karst faunal regions” (KFR’s), hypothesized as biologically discrete areas of cave habitat that are used to manage species recovery (U.S. Fish and Wildlife Service 1994; Veni 1992, 1994). Three KFR’s (Figs 61–62) are currently recognized in Bexar, Travis and Williamson Counties each of which includes large numbers of caves and encompasses the distributions of multiple endangered invertebrates. However, KFR boundaries are limited by existing taxonomy which in most cases does not accurately reflect species distributions (White and Carothers 2001).

This study revises the taxonomy of *Tayshaneta* based on the phylogenetic results of Ledford et al. (2011) and data collected from a morphological survey using scanning electron and compound light microscopy. Ten new species are described, along with three previously unknown sexes and all remaining species are imaged, diagnosed and keyed. The morphology and relationships within *Tayshaneta* are discussed and five species-groups are identified. Distribution maps are provided along with an evaluation of KFR’s based on revised species distributions. The primary objective of this study is to produce a functional taxonomy for *Tayshaneta* that will facilitate conservation and management efforts and contribute to an understanding of the Texas cave fauna.

## Materials and methods

### Taxon sampling

A resurgence of interest in Texas cave biology, driven largely by conservation efforts, has produced a wealth of new *Tayshaneta* specimens more than doubling records since Gertsch (1974). In order to prioritize collection sites, a database combining records for described species and all recent collections was developed. Collection sites were then selected to maximize sampling throughout known ranges with priority given to type localities. Outgroup selection was based on the most recent phylogenies of haplogyne spiders (Platnick et al. 1991; Ramírez 2000) and specimen availability. Between 1–10 individuals were collected from each site, placed directly into 95% ethanol and then transferred to storage at -20°C. Each specimen was assigned a unique voucher number and is accessioned in a database maintained at the California Academy of Sciences (CASC).

Voucher specimens for the study are deposited at the California Academy of Sciences (CASC), the Texas Memorial Museum (TMM), the Museum of Texas Tech University (TTU) and the Essig Museum, University of California, Berkeley (UCB).

Due to the sensitive nature of cave locations and in the spirit of respecting the rights of property owners and encouraging future research, precise locality information is not provided. Unless otherwise noted, all cave locations are limited to within 2 kilometers. Specimens used in this study along with their voucher codes are listed in Ledford et al. (2011) and a map highlighting the study area is provided in Fig 3.

Distribution maps were produced using Arc GIS 10.0 (Environmental Systems Research Institute, CA). Karst faunal region boundaries were derived from shape files provided by Zara Environmental (K. O’Connor) through the U.S. Fish and Wildlife Service.

### Morphology

Prior to examination with a Leo 1450VP Scanning Electron Microscope, all structures were cleaned with a fine brush or ultrasonicator and critical point dried. Best results were obtained by gradually dehydrating the specimen in increasing concentrations of ethanol for 24-48 hours prior to critical point drying. Dried specimens were then mounted on pin mount SEM stubs (Ted Pella Inc., Redding, USA) on copper-backed tape. Specimens were sputter coated for 120 seconds using a Denton Vacuum Sputter Coater. Large structures were photographed using a Nikon DMX1200 camera attached to a Leica MZ 16 stereomicroscope. Images were then montaged using Helicon Focus v. 4.2.1 (http://www/heliconsoft.com). For male specimens, the right palp was scanned and the left was maintained with the specimen post examination using compound light microscopy.

Vulvae were carefully excised and placed in a pancreatin solution for 24-48 hours to digest extraneous tissue (Àlvarez-Padilla and Hormiga 2008) then placed in water and manually cleaned. Best results were obtained by removing the cuticle from the dorsal surface of the abdomen and digesting the entire structure. If the vulva remained unclear, it was stained for one minute with Chlorazol Black and reexamined. Images of each species were prepared using a Nikon DMX1200 camera attached to a Leica DM 4000 compound microscope. Genitalia were placed in Hoyer’s solution and examined in well slides or temporary mounts following the procedure described by Coddington (1983).

Descriptions follow the format of Ledford and Griswold (2010) and Ledford (2004). Descriptions of previously unknown sexes were based upon individuals collected at the type locality. All measurements are in millimeters and quantify the structure at its widest or longest point. A summary of anatomical abbreviations used in the descriptions and keys is provided in Table 1. Individual images of all structures will be made available at the time of publication in Morphbank (www.morphbank.net) and species pages will be available in the Encyclopedia of Life (http://www.eol.org).

**Table 1.** List of Anatomical Abbreviations used in the text and figures.

**Abbreviation**	**Structure**
AER	Anterior Eye Row
AME	Anterior Median Eyes
At	Atrium
E	Embolus
PME	Posterior Median Eyes
RS	Retrolateral Sclerite
RTS	Retrolateral Tibial Sclerite
SH	Spermathecal Head
SS	Spermathecal Stalk
TS	Palpal Tarsus
VS	Ventral Sclerite

### Phylogeny

Detailed protocols for the extraction, amplification and sequencing of DNA are reported in Ledford et al. (2011). Three gene fragments were selected based on availability, prior use in systematics studies and amplification success. Mitochondrial cytochrome oxidase I (~800bp), nuclear histone 3 (~330bp) and 28s rDNA (~1000bp) were amplified following Ledford et al. (2011) and the primers and conditions used are reported in Table 3. Phylogenetic methods also follow Ledford et al. (2011) and both independent genes and concatenated data were analyzed under a variety of optimality criteria and conditions (Table 2). Sequence alignment was performed using CLUSTAL × v. 2.0 (Larkin et al. 2007) and additional 28s rDNA alignments were produced using Muscle v. 3.8 (Edgar 2004). Models of nucleotide evolution were selected using the Akaike Information Criterion (Akaike 1973) as implemented in MrModeltest v. 2.2 (Nylander 2004). Partitioning strategies for COI and histone 3 were evaluated using Bayes Factors (Brown and Lemmon 2007) for fully partitioned, partially partitioned and unpartitioned analyses.

Bayesian analysis was performed using MrBayes v. 3.1.2 (Huelsenbeck and Ronquist 2001) using four independent runs until the standard deviation of split frequencies fell below 0.01. Stationarity was evaluated by examining the stability of posterior probabilities for nodes of each MCMC run using the Cumulative and Compare plots in Are We There Yet? (http://ceb.csit.fsu.edu/awty; Nylander et al. 2008) and the first 25% of trees were discarded from the posterior distributions of each analysis. Maximum likelihood analysis was performed using 1000 bootstrap replicates in RAxML v. 7.0.4 (Stamatakis 2006) and parsimony analyses were performed in PAUP* (Swofford 2003) using 1000 iterations of a heuristic search holding 100 trees for each iteration, with random taxon addition and tree bisection-reconnection (TBR) branch-swapping. Branches were collapsed using the default rule in PAUP* v.4.0 b10 (collapse if maximum length is zero). Nonparametric bootstrap support values were calculated using 1000 replicate searches with random taxon addition.

Aligned data matrices and trees will be made available online in TreeBASE (http://www.treebase.org/).

**Table 2.** Summary tree statistics and conditions for each analysis.

**Analysis**	**Optimality Criterion, Software**	**Conditions**	**Statistics**
Concatenated	Parsimony, PAUP* v.4b10	1000 iterations, heuristic search with TBR	130 trees, 8124 steps
COI (full partitions)	Likelihood, RAxML v.7.0.4	1000 non-parametric bootstrap replicates	-lnL 16062.90
Histone 3 (full partitions)			-lnL 2910.70
28s rDNA			-lnL 20318.74
Three-gene concatenated			-lnL 34742.79
Two-gene concatenated (COI, 28s)			-lnL 34550.09
COI (full partitions)	Bayesian, Mr. Bayes v.3.1.2	20,000,000 generations, burnin= 25%	sdsf 0.003
COI (1^st^ and 2^nd^, 3^rd^ positions)			sdsf 0.003
COI (unpartitioned)			sdsf 0.003
Histone 3 (full partitions)			sdsf 0.004
Histone 3 (1^st^ and 2^nd^, 3^rd^ positions)			sdsf 0.01
Histone 3 (unpartitioned)			sdsf 0.03
28s rDNA			sdsf 0.008
Three-gene concatenated		50,000,000 gen, burnin= 25%	sdsf 0.05
Two-gene concatenated (COI, 28s)		20,000,000 gen, burnin= 25%	sdsf 0.01

**Table 3.** Primer sequences, source and annealing temperatures. Optimized annealing temperatures in bold.

**Gene**	**Forward**	**Sequence**	**Reference**	**Reverse**	**Sequence**	**Reference**	**Annealing Temperature**
COI	1718	5-ggA ggA TTT ggA AAT TgA TTA gTT CC-3	Simon et al. (1994)	2568	5-gCT ACA ACA TAA TAA gTA TCA Tg-3	Simon et al. (1994)	44-50, **48°C**
COI	1751	5 -gAg CTC CTg ATA TAg CTT TTC C-3	Simon et al. (1994)	2568	5-gCT ACA ACA TAA TAA gTA TCA Tg-3	Simon et al. (1994)	44-50, **48°C**
COI	PMT1	5-GGT CAA CAA ATC ATA AAG ATA TTG G-3	Folmer et al. (1994)	2568	5-gCT ACA ACA TAA TAA gTA TCA Tg-3	Simon et al. (1994)	44-50, **45°C**
COI	1490-ONO	5-CW ACA AAY CAT ARR GAT ATT GG-3	Simon et al. (1994)	2568	5-gCT ACA ACA TAA TAA gTA TCA Tg-3	Simon et al. (1994)	44-50, **45°C**
COI	2309	5-TTT ATg CTA TAg TTg gAA TTg g-3	Simon et al. (1994)	2776	5-ggA TAA TCA gAA TAN CgN CgA gg-3	Simon et al. (1994)	44-50, **48°C**
28srDNA	ZX1	5-ACC CGC TGA ATT TAA GCA TAT-3	Mallatt and Sullivan (1998)	ZR2	5-CCG AAG TTT CCC TCA GGA TAG C-3	Mallatt and Sullivan (1998)	50-60, **55°C**
28srDNA	28sOCS	5-CGT GAA ACT GCT CAG AGG-3	Miller et al. (2010)	28sC	5-GGC GAA AGA CTA ATC GAA CC-3	Miller et al. (2010)	50-60, **55°C**
Histone 3	H3af	5-ATG GCT CGT ACC AAG CAG ACV GC-3	Colgan et al. (1998)	H3ar	5-ATA TCC TTR GGC ATR ATR GTG AC-3	Colgan et al. (1998)	48-55, **50°C**
Histone 3	H3nf	5-ATG GCT CGT ACC AAG CAG AC-3	Colgan et al. (1998)	H3nr	5-ATR TCC TTG GGC ATG ATT GTT AC-3	Colgan et al. (1998)	48-55, **50°C**

## Results

### Morphology

Exemplars for each *Tayshaneta* species, including undescribed species discovered during the course of this study, were photographed using automontage, compound and scanning electron microscopy. Holotype specimens for each species were examined in order to confirm the identity of exemplars used in analyses.Images provided in this study are either taken directly from the holotype or from specimens collected at the type locality. Over 3,000 images were produced based on a set of standardized views and assembled into comparative plates. Careful attention was directed at diagnostic characters provided in Gertsch (1974) and to somatic features in order to assess variation in troglomorphic morphology.

Putativesynapomorphies for *Tayshaneta* include a unique conformation of the female genitalia, with short spermathecal stalks bearing large heads (SH, Figs 52–54) and the recurved to straight retrolateral spine on the male palpal tibia (RTS, Figs 32A–F). Body color ranges from pale brown-yellow to depigmented with faint dark patterns surrounding the eyes and ocular area. The legs are covered in fine setae and bear few scattered spines. A ventroapical preening comb on metatarsus III was observed in each species examined (Figs 12–13 in Ledford 2004). Patellar and tibial gland morphology was similar to that described by Platnick (1986) with triangular patellar plates bearing single small pores (Figs 30–31, 33, 38, 40, 46 in Platnick 1986). The abdomen lacks distinctive patterning, is sparsely setose and pale yellow to white in color. Spinning organs follow the descriptions of *Leptoneta infuscata* Simon, 1872 (Ledford and Griswold 2010) and *Calileptoneta* (Ledford 2004) with the exception of bearing fewer aciniform gland spigots (6–10) on the PMS and PLS (Figs 11A–C).

In contrast to other leptonetine genera, the palpal morphology of *Tayshaneta* is relatively conserved and the bulb bears few spines, specialized setae, or accessory sclerites. The shape of the palpal tarsus is of two basic types; divided, as in *Tayshaneta fawcetti* sp. n. (Fig. 31D) and tapering, as in *Tayshaneta coeca*, *Tayshaneta microps* and *Tayshaneta paraconcinna* (Figs 31A–C). The depth of the division ranges from deeply divided as in *Tayshaneta fawcetti* sp. n. and *Tayshaneta vidrio* sp. n. (Figs 31D–E) to weakly divided or swollen as in *Tayshaneta madla* sp. n. (Fig. 31F). An exposed tarsal organ is present dorsoapically and consists of a shallow circular base with a pair of round receptors (Figs 24G–H in Ledford et al. 2011). The embolus is weakly sclerotized, transparent and connected via a short tube to a large reservoir in the bulb (Figs 30A–D in Ledford et al. 2011). The sculpture along the margins of the embolus ranges from smooth as in *Tayshaneta coeca* and *Tayshaneta myopica* (Figs 36D, 44D) to bearing tooth-like extensions and folds as in *Tayshaneta anopica* (Fig. 33D). The embolus is typically curved or folded around the ventroapical portion of the bulb and bears a single, circular opening (Fig. 44F).

The ventral sclerite (VS) is a single, spine-like projection that extends approximately half the length of the embolus. The position and length of the VS ranges from elongate and mesal as in *Tayshaneta fawcetti* sp. n. (VS, Fig. 40E), to retroventral as in *Tayshaneta myopica* (VS, Fig. 44B and short as in *Tayshaneta sprousei* sp. n. (VS, Fig. 48E) The VS is absent in several species, including *Tayshaneta coeca* (Fig. 36E) and despite repeated efforts to determine whether this structure was related to expansion no VS was observed. The retrolateral sclerite (RS) is of two types, a shallow, pocket-like invagination as in *Tayshaneta fawcetti* sp. n. (RS, Fig. 40E–F) or a distinctly separated, oval sclerite as in *Tayshaneta whitei* sp. n. (RS, Figs 51D–E).

The retrolateral tibial spine (RTS) is recurved to straight and ranges from short, occupying less than half the length of the palpal tarsus (RTS, Figs 31A–B, D, 32A–B, 36F), to elongate in which the spine extends greater than half the length of the palpal tarsus (RTS, Figs 31C, F, 32C, F). The RTS is situated on a shallow to pronounced base and is moveable, possibly serving as a positioning structure during mating. A fine, comb-like sculpturing extends along the entire length of the RTS in most species, but may also be smooth near the base as in *Tayshaneta fawcetti* sp. n. (Fig. 32D) and *Tayshaneta devia* (Fig. 32B). Between three and four flattened setae are located near the base of the RTS (Figs 32A–F) along with several unmodified setae surrounding the base.

Examination of female genitalia using compound microscopy revealed relatively little variation among species and in most cases female specimens appear nearly identical in structural details (Figs 52–54). The preparation of female genitalia was problematic as the weakly sclerotized spermathecal stalks do not remain in a fixed position and slight differences in orientation can dramatically alter the structure’s appearance. Even with careful preparation techniques the vulva is difficult to precisely position for comparison among individuals. The atrium is suboval to triangular and covered in fine pores. The spermathecal stalks are twisted and connect to the atrium basally via short sclerotized tubes. The spermathecal heads are swollen, circular (Figs 52A, C–F, 53A–B, D–F, 54A–C, E) to elongate (Figs 53C, 54D) and covered in fine pores.

### Phylogeny

Results of phylogenetic analyses follow Ledford et al. (2011) and summary statistics for each analysis are presented in Table 2. Phylograms for concatenated analyses (Bayesian, maximum likelihood, parsimony) are presented in Figures 4-6 and independent gene trees are in Figures 7-9. Nodes with a posterior probability of 95% and greater are considered supported and all remaining nodes are collapsed. Nodes for maximum likelihood and parsimony analyses with bootstrap support values of 75% and greater are considered supported and all remaining nodes are collapsed.

Tree topologies are identical to Ledford et al. (2011) and few instances of conflict between analyses are observed. *Tayshaneta* monophyly is corroborated by all analyses although its relationship to other North American leptonetid genera is ambiguous (Figs 4-9). Eight described species are represented in the analyses, including *Tayshaneta anopica* (Gertsch, 1974), *Tayshaneta bullis* (Cokendolpher, 2004), *Tayshaneta coeca* (Chamberlin & Ivie, 1942), *Tayshaneta concinna* (Gertsch, 1974), *Tayshaneta devia* (Gertsch, 1974), *Tayshaneta microps* (Gertsch, 1974), *Tayshaneta myopica* (Gertsch, 1974) and *Tayshaneta paraconcinna* (Cokendolpher & Reddell, 2001). Five undescribed species are also represented, *Tayshaneta fawcetti* sp. n. (Figs 19, 40), *Tayshaneta madla* sp. n. (Figs 21, 42), *Tayshaneta oconnorae* sp. n. (Figs 24, 45), T*. sandersi* sp. n. (Figs 26, 47) and *Tayshaneta whitei* sp. n. (Figs 30, 51), each of which has diagnostic morphology.

Four clades recovered by analyses are identified as species-groups in the discussion: 1) the *anopica* group, consisting of *Tayshaneta anopica* + *Tayshaneta concinna* (Node A), 2) the *myopica* group, consisting of *Tayshaneta myopica* + *Tayshaneta paraconcinna* (Node B), 3) the *microps* group, consisting of *Tayshaneta microps* + *Tayshaneta madla* (Node C) and 4) the *sandersi* group, consisting of *Tayshaneta sandersi* sp. n. + *Tayshaneta whitei* sp. n. (Node D). Although conflict among trees is limited, the resolution among three species (*Tayshaneta bullis*, *Tayshaneta coeca*, *Tayshaneta devia*) in concatenated analysis differs with results from independent analysis of COI and 28s rDNA. In both gene trees, *Tayshaneta devia* is supported (Node E, Fig. 7) and in the COI tree a sister group relationship is recovered with *Tayshaneta coeca* (Node F, Fig. 7). Furthermore, the COI tree supports *Tayshaneta bullis* as sister to the *microps* species-group (Node G, Fig. 7). However, *Tayshaneta devia* is not supported by concatenated analyses and relationships among *Tayshaneta coeca* and *Tayshaneta bullis* are unresolved.

## Discussion

Among the most interesting results of the phylogenetic analyses is the contrast in branch lengths between *Tayshaneta* and the remaining North American genera (Figs 4-6). Although sampling and rate variation among genes (Figs 7-9) are known to affect branch lengths, the close relationships, morphological similarity and narrow geographic distributions of *Tayshaneta* suggest that it is a relatively recent radiation of species. Similar radiations are known for *Cicurina* spiders (Paquin and Dupérré 2009; Paquin and Hedin 2004) and *Texella* harvestmen (Ubick and Briggs 1992, 2004) both of which show similar biogeographic patterns and affinity for caves. Recent work has shown that the diversification patterns of *Cicurina* is correlated with the complex faulting in the region (White et al. 2009) and may serve as a general model to explain the diversity of the Texas cave fauna. On-going work has been directed at synthesizing the distributions for multiple cave invertebrates in order to develop a comprehensive understanding of the Texas fauna (Reddell et al. in prep.).

Although most *Tayshaneta* species have relatively conserved genitalic morphology, intraspecific variation in somatic features related to cave life (troglomorphism) is extreme and often includes a range of eye and pigment reduction. In *Tayshaneta myopica*, for example, multiple morphotypes are often found within a narrow geographic distribution and range from darkly pigmented, large-eyed individuals (Figs 55E–F) to lightly pigmented, reduced-eyed forms (Fig. 55A–C), to complete eye and pigment loss (Fig. 55D). While these differences likely indicate varying degrees of local adaptation to caves, the intergradient morphologies observed suggest that some species may have an adaptive cline from surface to cave-adapted morphotypes. Similar patterns of troglomorphic variation have been reported in *Texella* harvestmen that show multiple degrees of troglomorphic morphology between closely related species (Ubick and Briggs 1992, 2004). In *Tayshaneta reddelli* and *Tayshaneta reyesi* for example, species limits are often indistinct as specimens show a gradual reduction in eyes, pigment and tubercles on the carapace. One intriguing hypothesis is that populations are actively colonizing caves and becoming increasingly more troglomorphic, similar to the adaptive shift model proposed for Hawaiian isopods (Rivera et al. 2002).

Biogeographic relationships within *Tayshaneta* reflect the fragmented geology of region as distributions are allopatric and few cases of sympatry are known. However, distributions for most species remain poorly characterized and reflect incomplete sampling, especially of surface localities, which are rarely inventoried as part of cave surveys. Species distributions in Bexar and Travis Counties are particularly complex and several undetermined records (Fig. 61) likely represent range extensions or additional species, the identification of which will help resolve areas of taxonomic ambiguity. The most significant area of biogeographic ambiguity are caves and surface habitats in Comal and Hays Counties both of which remain poorly inventoried and are essential to resolving species limits, especially between *Tayshaneta coeca* (Chamberlin & Ivie, 1942) and *Tayshaneta devia* (Gertsch, 1974).

The majority of species described by Gertsch (1974) were known from single localities and was used as the primary justification for the endangered status of *Tayshaneta microps* (Gertsch, 1974) and *Tayshaneta myopica* (Gertsch, 1974) (U.S. Fish and Wildlife Service 1994, 2000). Recent sampling efforts, combined with the molecular and morphological data presented in this study, have shown that most species are more broadly distributed than expected but still of limited distribution. Furthermore, molecular data suggest that most troglobitic species are actively using subterranean microfissures and voids as corridors for dispersal between caves. The most striking examples are for the species *Tayshaneta anopica* (Gertsch, 1974), *Tayshaneta myopica* (Gertsch, 1974) and *Tayshaneta sandersi* sp. n. each of which have populations in different caves that share identical haplotypes for the loci surveyed in this study. While these connections are not surprising given the geology of the area, they set a precedent for interpreting the distribution of other *Tayshaneta* species and are likely to effect conservation and management decisions.

### Karst faunal regions

Karst faunal regions (KFR’s) were originally developed as tools to aid the recovery of endangered karst invertebrates by identifying geologically independent regions that had a relatively high proportion of endemic species (Veni, 1992, 1994). Although an evolutionary model was not explicitly proposed, the inherent reasoning is that the present distribution of the karst invertebrate fauna can be explained by the fragmented geology of the region (White and Carothers 2001). Although recent work has shown that phylogenetic divergence within *Cicurina* spiders is likely correlated with faulting, the distributions of most invertebrate groups are poorly understood which precludes a synthesis of biogeographic patterns in the area. Furthermore, the endemicity index used to help define KFR’s is necessarily constrained by existing taxonomy, most of which is inadequately resolved or erroneous (Paquin and Dupérré 2009).

While the distribution of *Tayshaneta* is broader than anticipated, it is nevertheless highly restricted, especially when compared to other endangered invertebrate groups. *Cicurina* and *Texella*, for example, have highly active hunting lifestyles and are known to occur in far more caves. In contrast, *Tayshaneta* are more sedentary, spending most of their lives in webs with the exception of males that may leave the web upon maturity. Not surprisingly, the distribution of most *Tayshaneta* species closely corresponds to established KFR’s. In Bexar County, *Tayshaneta microps* is restricted to the Government Canyon KFR (Fig. 63) and despite extensive sampling no additional populations have been discovered. *Tayshaneta madla* sp. n. and *Tayshaneta whitei* sp. n., however, occur in multiple KFR’s and although are not currently listed as endangered show that KFR’s are not biologically exclusive as presently defined. *Tayshaneta myopica* (Fig. 62) shows a similar pattern in Travis County, where most populations are known from the Jollyville KFR as well as in the McNeil/ Round Rock KFR.

Following the arguments of White and Carothers (2001), the presentation of these data is not designed to be a critique of the KFR strategy but rather highlights that the geological complexity and phylogenetic histories of invertebrates in the region make the delineation of boundaries a daunting task. From a conservation perspective, the use of KFR’s have been successful at acquiring new cave habitat and establishing karst preserves, both of which are essential to the long-term protection of the karst invertebrate fauna. As recovery plans, local initiatives and monitoring continue to develop in the region taxonomic studies that integrate all available data will be essential to the successful implementation of the KFR conservation strategy.

## Taxonomy

### Key to species of Tayshaneta

The key presented here relies heavily on fine details of the male and female genitalia, some features of which are not visible using conventional light microscopy or without special preparation techniques. Scanning electron and compound light microscopy is essential for positive identification and the females of most species are not diagnosable in the absence of associated males.

**Table:** 

1	Male palpal tarsus tapering apically, rarely with weak division (Figs 31A–C); ventral sclerite present or absent; embolus rounded to rectangular; retrolateral sclerite not pocket-like or absent; females with round spermathecal heads (Figs 52A–F, 53A, D–F, 54A–C, E)	2 Note. The division of the palpal tarsus in some species is very weak and often appears entire except under high magnification (Fig. 31F).
–	Male palpal tarsus with strong apical division (Figs 31D–F); ventral sclerite present; embolus rectangular to bifurcate; retrolateral sclerite pocket-like; females usually with elongate spermathecal heads (Figs 53B–C; 54D)	15
2	Bulb bearing a prominent ventral sclerite (VS, Figs 33, 37, 44-48); embolus rounded to sculptured along margin, with or without a large basal tooth or fold (Figs 33, 37, 45, 47)	3
–	Bulb lacking a ventral sclerite (Figs 34–36, 38, 42–43, 51); embolus rounded to distinctly sculptured along margin, with or without a small basal tooth (Figs 42–43, 51)	9
3	Ventral sclerite elongate (VS, Figs 44E, 46E) to greatly reduced (VS, Fig. 48E); embolus rounded and spoon-shaped (E, Figs 44D, 46D) to suboval (E, Fig. 48D), lacking sculpture along margin	4
–	Ventral sclerite elongate; embolus shape irregular, with sculpture, bifurcation, or large basal tooth along margin (E, Figs 33D, 37D, 45D, 47D)	6
4	Ventral sclerite short, occupying less than the width of the embolus (VS, Figs 48B, D–F); retrolateral tibial spine elongate, straight to slightly curved, at least 0.50×tarsus length; embolus suboval, flush with apical portion of bulb (E, Fig. 48D–F)	*Tayshaneta sprousei* sp. n. Dist. Known from two caves on Camp Bullis, Bexar County, Texas (Fig. 59).
–	Ventral sclerite elongate, occupying at least 0.50× length of apical portion of bulb (VS, Figs 44E, 46E); retrolateral tibial spine recurved, short, occupying much less than 0.50× length palpal tarsus (RTS, Figs 32A, 44A, 46A); embolus spoon shaped, apically extended beyond bulb (E, Figs 44E, 46E)	5
5	Base of embolus sharply curved, projecting ventrally (Fig. 44D), smooth along its margin; embolus folded over apical portion of bulb (E, Fig. 44E); retrolateral tibial spine weakly recurved, on elevated base (RTS, Fig. 44A); eyes and pigmentation variable, but usually greatly reduced (Figs 23A–F); large, thin spiders, length femur I 1.5–2.0× carapace length	*Tayshaneta myopica* (Gertsch, 1974) Dist. Caves of Travis and Williamson counties, Texas (Fig. 57).
	Notes. *Tayshaneta myopica* and *Tayshaneta paraconcinna* are difficult to separate and require close inspection of subtle genitalic characters, preferably using scanning electron microscopy. In several cases, particularly in Williamson County, these species are only reliably diagnosed using a combination of genitalic morphology and molecular data.
–	Base of embolus weakly curved, projecting anteriorly, often with an apical cleft (E, Fig. 46D); retrolateral tibial spine sharply recurved, pick-like, on short base (RTS, Fig. 46A); eyes and pigmentation variable, but usually darkly pigmented with well-developed eyes (Figs 25A–F); short, robust spiders, length femur I 1.2–1.5× carapace length	*Tayshaneta paraconcinna* (Cokendolpher & Reddell, 2001) Dist. Broadly distributed in caves and surface habitats from Bell County South through Williamson, Travis and Blanco Counties (Fig 57).
6	Depigmented, blind spiders (faint eyespots may be present under high magnification); length femur I at least 1.90× carapace length; retrolateral tibial spine short to elongate; embolus narrowly or broadly bifurcate, with or without large basal tooth	7
–	Pigmented, large-eyed spiders with dark patterns surrounding the ocular area; length femur I 1.0–1.5× carapace length; retrolateral tibial spine short, occupying less than 0.5× length of palpal tarsus; embolus broad, with prominent basal tooth	*Tayshaneta concinna* (Gertsch, 1974) Dist. Caves and surface habitats in Travis County, Texas (Fig. 56).
7	Embolus bifurcate, with sharp lobes (E, Figs 33D, 47D); ventral sclerite positioned retrolaterally, base indistinct; retrolateral tibial spine short to elongate	8
–	Embolus bifurcate, with rounded lobes (E, Fig. 45D); ventral sclerite positioned mesally, on distinct base (VS, Fig. 45E); retrolateral tibial spine short, occupying less than 0.5× length tarsus	*Tayshaneta oconnorae* sp. n. Dist. Known from two caves in Southern Hays County, Texas (Fig. 58).
8	Embolus broad, with distinct basal tooth (E, Fig. 33D); ventral sclerite straight; retrolateral tibial spine elongate, occupying at least 0.50× length of palpal tarsus	*Tayshaneta anopica* (Gertsch, 1974) Dist. Known from two caves in Northern Williamson County, Texas (Fig. 56).
–	Embolus narrow, bifurcate (E, Fig. 47D); ventral sclerite prolaterally curved (VS, Fig. 47E); retrolateral tibial spine short, occupying less than 0.5× length of palpal tarsus	*Tayshaneta sandersi* sp. n.
	Dist. Known from three caves in the Onion Creek watershed of Barton Springs, Travis County, Texas (Fig. 56).
9	Embolus rounded to rectangular, lacking basal tooth and with minimal sculpture along margins (E, Figs 34–36, 38); retrolateral tibial spine short to elongate, recurved or straight	10
–	Embolus rounded to rectangular, with prominent basal tooth (E, Figs 42, 43, 51); retrolateral tibial spine elongate, occupying at least 0.50× length of the palpal tarsus	13
10	Embolus oval or tapering apically (E, Figs 34D, 38D), flush or extended beyond apical portion of bulb (Figs 34B, 38B); retrolateral tibial spine short, occupying less than 0.50× length of palpal tarsus	11
–	Embolus rectangular to gently curved along its base, flush with apical portion of bulb; retrolateral tibial spine short to elongate	12
11	Eyes reduced, lacking pigmentation near ocular area (Figs 13A–F); femur I 1.35–1.75× carapace length; embolus rectangular to oval, not tapering apically (E, Fig. 34D), retrolateral tibial spine sculptured along its entire length (RTS, Fig. 34A)	*Tayshaneta archambaulti* sp. n. Dist. Known from two caves in Southern Hays County, Texas (Fig. 58).
–	Eyes large and darkly pigmented near ocular area (Figs 17A–E); femur I 1.0–1.3× carapace length; embolus tapering apically, extending beyond apical portion of bulb (E, Fig. 38D); retrolateral tibial spine stout, distinctly lacking sculpture along its base (RTS, Figs 31B, 32B)	*Tayshaneta devia* (Gertsch, 1974) Dist. Known from caves and surface habitats in Southern Williamson through Travis Counties, Texas (Fig. 56).
12	Embolus rectangular, folded apically (E, Figs 35D–F); retrolateral tibial spine elongate, occupying at least 0.50× length of the palpal tarsus (RTS, Fig. 35A)	*Tayshaneta bullis* (Cokendolpher, 2004) Dist. Known from two caves on Camp Bullis, Bexar County, Texas (Fig. 59).
–	Embolus rectangular and gently curved along its base (E, Fig. 36D); retrolateral tibial spine short, occupying less than 0.50× length of palpal tarsus (RTS, Fig. 36F)	*Tayshaneta coeca* (Chamberlin & Ivie, 1942) Dist. Known from caves and surface habitats in Hays through Comal Counties, Texas (Fig. 58).
13	Eyes and pigmentation variable, greatly reduced in one species (Figs 22A–F, 30A–F); palpal tarsus tapering apically (Figs 31A–C); femur I 1.3–1.9× carapace length; embolus with small basal tooth, rounded to quadrate apically (E, Figs 43D, 51E)	14
–	Eyes large and darkly pigmented near ocular area (Figs 21A–C); palpal tarsus weakly divided apically (TS, Fig. 31F); femur I 1.3–1.7× carapace length; embolus with large basal tooth, rounded at apex (E, Fig. 42D)	*Tayshaneta madla* sp. n. Dist. Known from caves and surface habitats in Bexar County, Texas (Fig. 59).
14	Eyes and pigment greatly reduced, only faint eyespots present (Figs 10A–B, 22A–F); retrolateral sclerite absent; embolus distinctly rounded at apex (E, Fig. 43D)	*Tayshaneta microps* (Gertsch, 1974) Dist. Known only from Government Canyon Bat Cave, Bexar County, Texas (Fig. 58).
–	Eyes and pigment normal, with dark marking surrounding ocular area (Figs 30A–F); retrolateral sclerite present, distinctly separated from bulb (RS, Figs 51D–E); embolus quadrate, curved apically (E, Fig. 51D)	*Tayshaneta whitei* sp. n. Dist. Known from caves in Bexar and Medina Counties, Texas (Fig. 59).
15	Embolus with distinctive basal tooth, shape rectangular to bifurcate (E, Figs 40F, 49D)	16
–	Embolus smooth along margins, shape oval to subquadrate, with weakly developed basal swelling or absent (E, Figs 39D, 41D, 50D)	17
16	Eyes and pigment greatly reduced (Figs 19A–F), femur I 1.60-1.83× carapace length; embolus rectangular, with distinctive basal tooth (E, Fig. 40F); retrolateral tibial spine short, smooth at base (RTS, Figs 40A, D); female genitalia with elongate spermathecal heads (SH, Fig. 53C)	*Tayshaneta fawcetti* sp. n. Dist. Known only from Fawcett’s Cave in the Devil’s River State Natural Area, Val Verde County, Texas (Fig. 60).
–	Eyes and pigment variable (Figs 28A–F), femur I 1.40–1.50× carapace length; embolus with large basal tooth and distinctive fold (E, Fig. 49D); retrolateral tibial spine elongate, sculptured along its length (RTS, Fig. 49A); female genitalia with circular spermathecal heads (SH, Fig. 54C)	*Tayshaneta valverdae* (Gertsch, 1974) Dist. Known from caves and surface habitats in Bandera, Uvalde and Val Verde Counties, Texas (Fig. 60).
17	Embolus oval, with or without apical fold (E, Figs 41D, 50D); ventral sclerite reduced or bifurcate apically (VS, Figs 41E, 50D–F); females with elongate spermathecal heads (SH, Fig. 54D)	18
–	Embolus rectangular, tapering apically, with weak basal swelling (E, Fig. 39D); ventral sclerite stout (VS, Fig. 39E); females with large, circular spermathecal heads (SH, Fig. 53B)	*Tayshaneta emeraldae* sp. n. Dist. Known only from Emerald Sink, in Western Val Verde County, Texas (Fig. 60).
18	Eyes and pigment reduced (Figs 29A–C), femur I 1.57–1.84× carapace length; ventral sclerite with distinctive division apically (VS, Figs 50D–F); embolus elongate, oval and without apical fold (E, Fig. 50D); female genitalia with elongate spermathecal heads (SH, Fig. 54D)	*Tayshaneta vidrio* sp. n. Dist. Known only from 400ft. Cave, Brewster County, Texas (Fig. 60).
–	Eyes and pigment normal (Figs 20A–C), femur I 1.8× carapace length; ventral sclerite reduced (VS, Fig. 41E); embolus oval with distinctive apical fold (E, Fig. 41D)	*Tayshaneta grubbsi* sp. n. Dist. Known only from Litterbarrel Cave, Val Verde County, Texas (Fig. 60).

**Figure 1.**
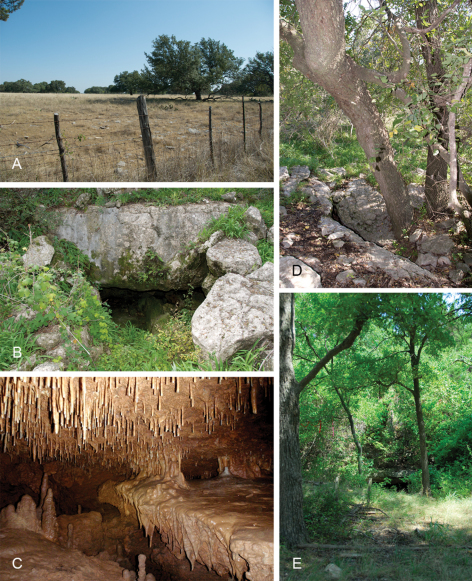
Images of habitat and cave entrances for *Tayshaneta* species. **A** Cobb’s Ranch, near Cobb’s Caverns, Williamson County, Texas, type locality for *Tayshaneta anopica* (Gertsch, 1974) showing karstic terrain **B** Entrance to Government Canyon Bat Cave, Bexar County, Texas, type locality for *Tayshaneta microps* (Gertsch, 1974) **C** General habitat of *Tayshaneta sandersi* sp. n., District Park Cave, Travis County, Texas, (M. Sanders) **D** Entrance to Lithic Ridge Cave, Bexar County, Texas, type locality for *Tayshaneta whitei* sp. n. **E** Entrance to Three Miles Bat Cave, Williamson County, Texas.

**Figure 2.**
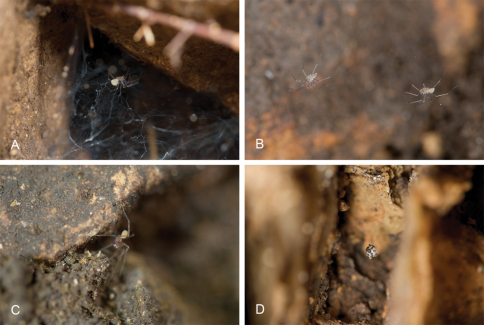
Live images of *Tayshaneta* species. **A** *Tayshaneta myopica* (Gertsch, 1974), female, Geode Cave, Travis County, Texas **B** *Tayshaneta fawcetti* sp. n., male and female in web, Fawcett’s Cave, Val Verde County, Texas **C** *Tayshaneta myopica* (Gertsch, 1974), male, Tooth Cave, Travis County, Texas **D** Egg-sac of *Tayshaneta anopica*, Corn Cobb’s Cave, Williamson County, Texas.

**Figure 3.**
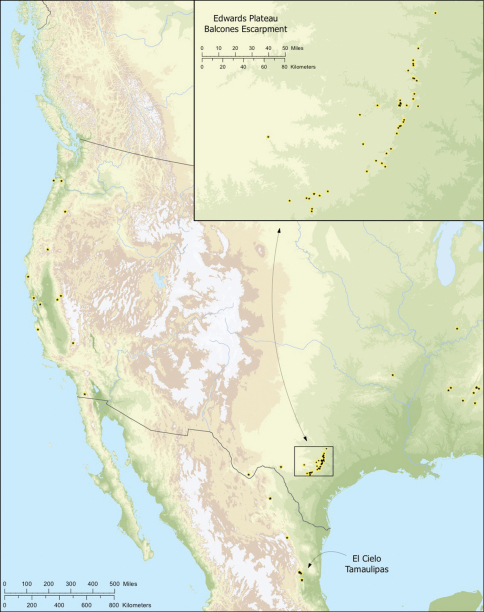
Map of the study area, with an inset highlighting the distribution of *Tayshaneta* on the Edward’s Plateau.

**Figure 4.**
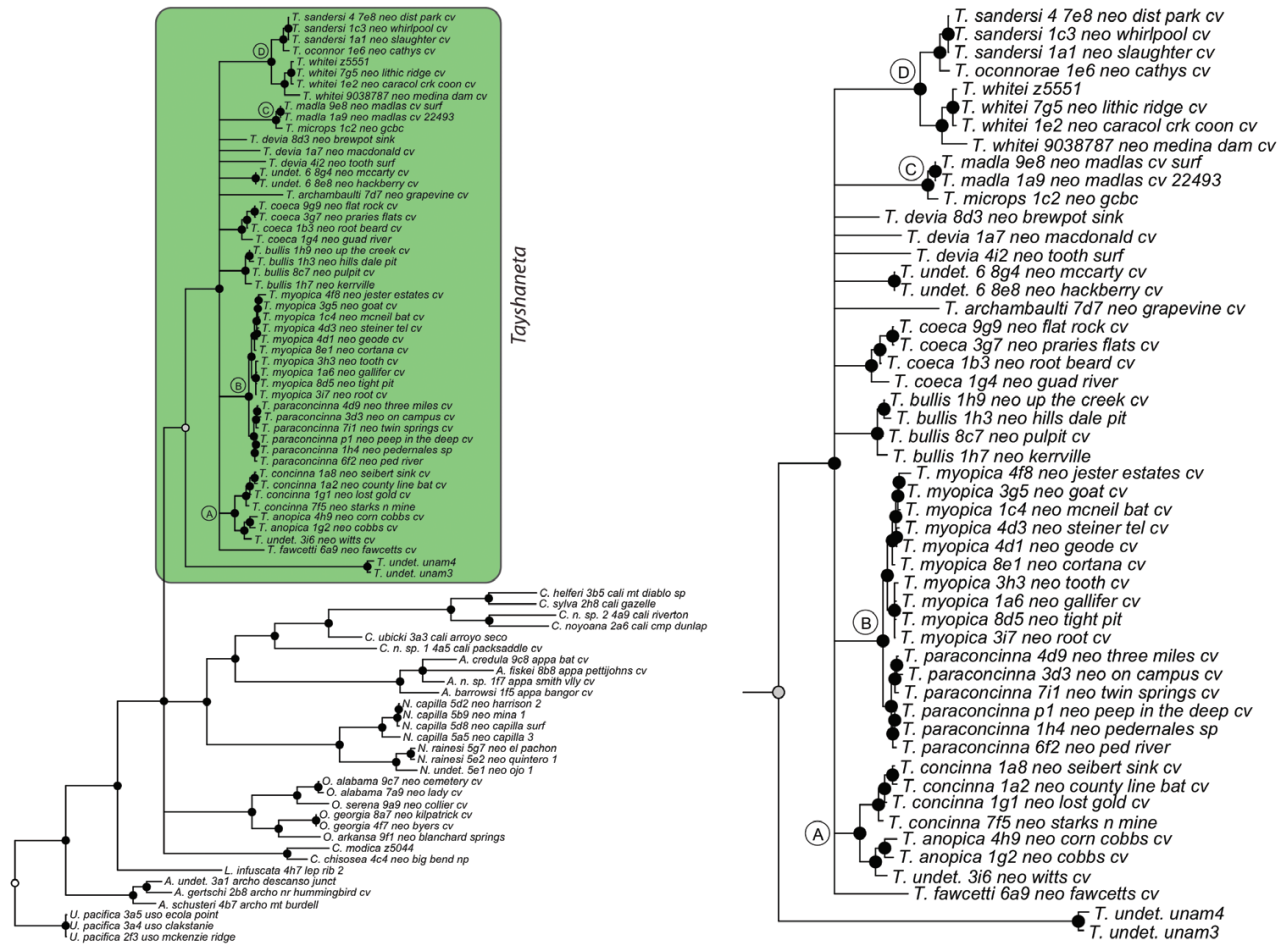
Three gene concatenated Bayesian phylogeny from Ledford et al. (2011). Highlighted and enlarged area indicates *Tayshaneta*. Black nodes correspond to a posterior probability >95%. *Tayshaneta* highlighted in green and enlarged at right. **A** *anopica* species-group **B** *myopica* species-group **C** *microps* species-group **D** *sandersi* species-group.

**Figure 5.**
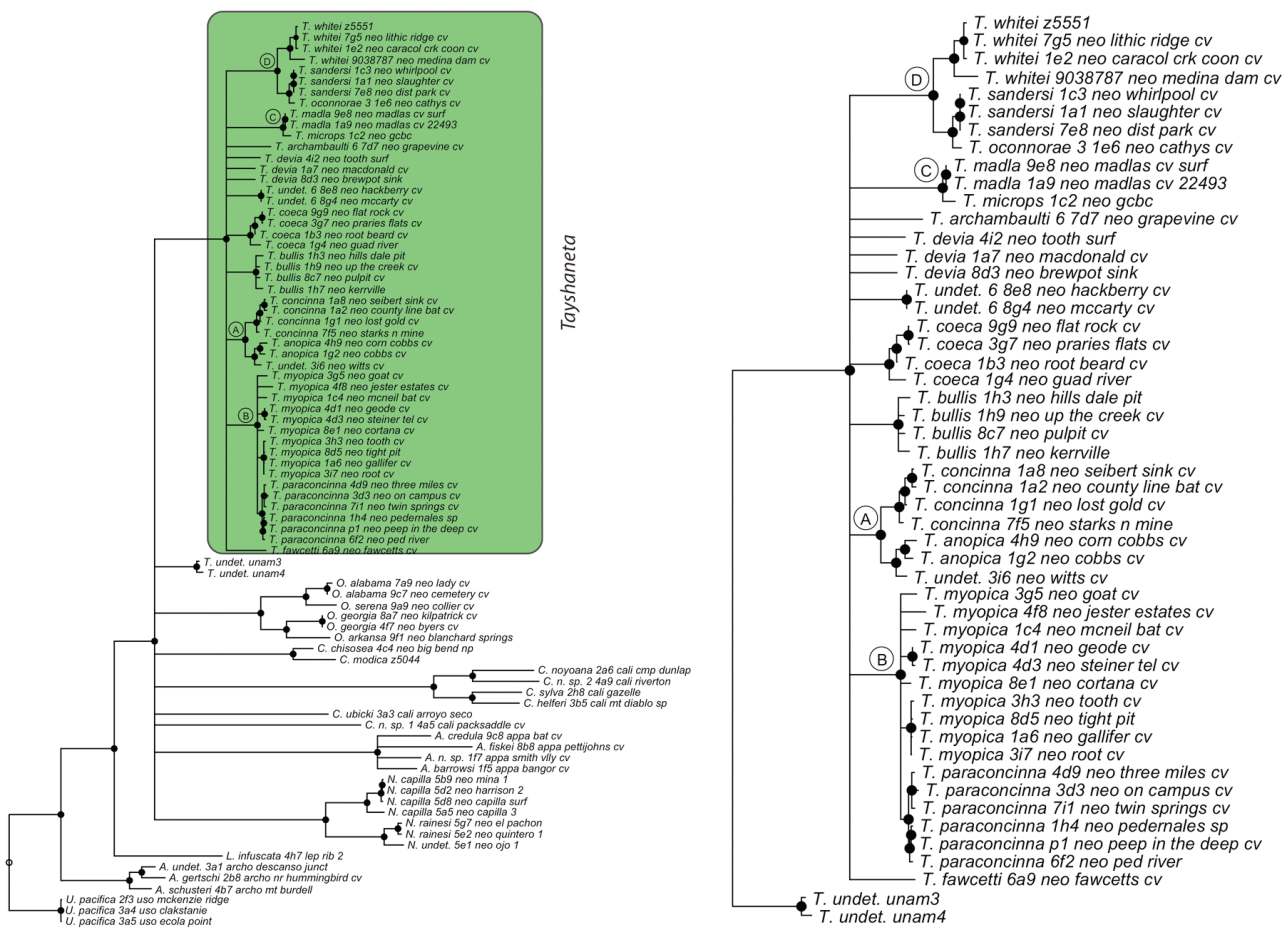
Three gene concatenated maximum likelihood phylogeny from Ledford et al. (2011). Highlighted and enlarged area indicates *Tayshaneta*. Black nodes correspond to bootstrap support >75%. *Tayshaneta* highlighted in green and enlarged at right. **A** *anopica* species-group **B** *myopica* species-group **C** *microps* species-group **D** *sandersi* species-group.

**Figure 6.**
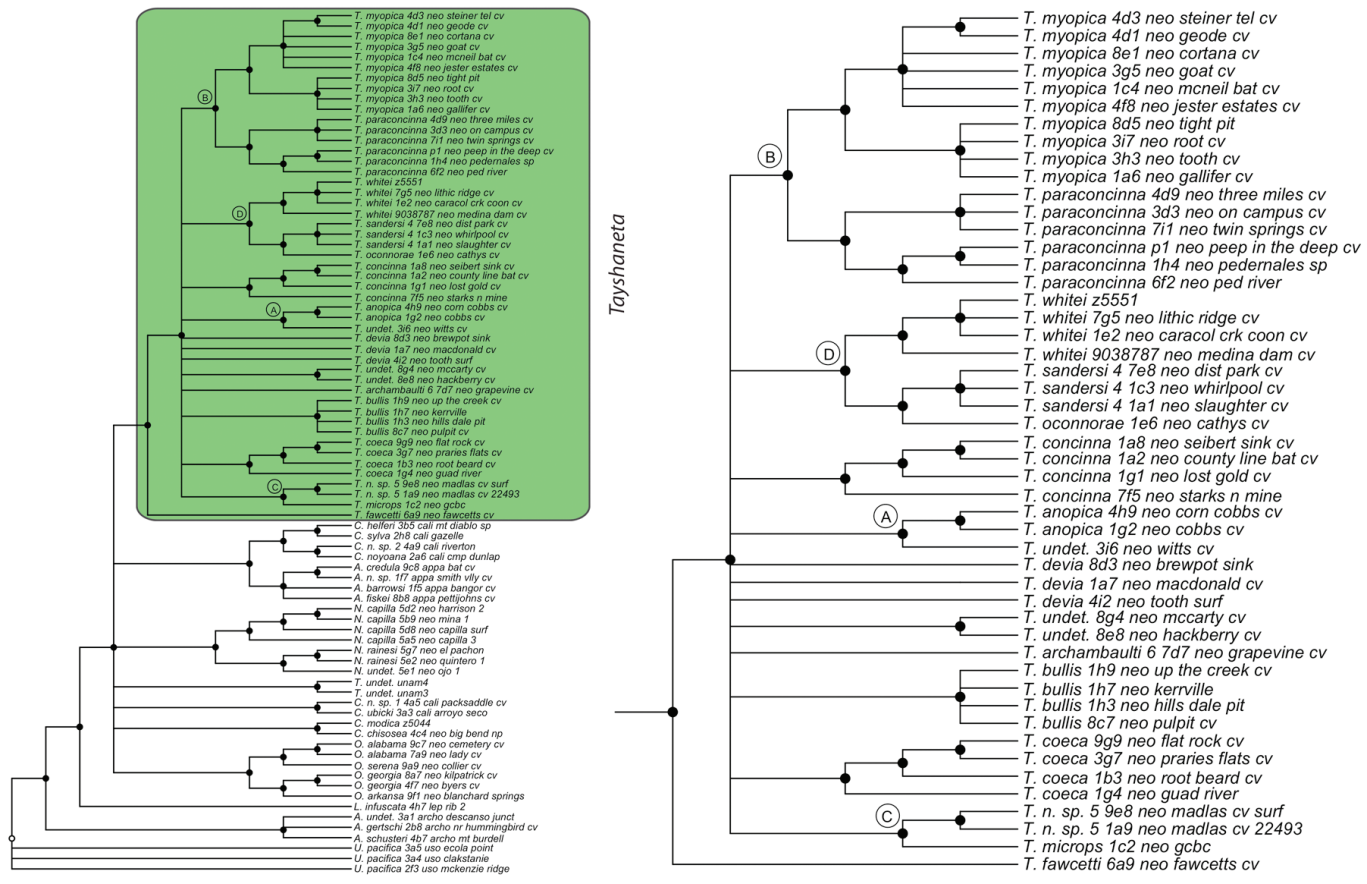
Three gene concatenated parsimony phylogeny from Ledford et al. (2011). Highlighted and enlarged area indicates *Tayshaneta*. Black nodes correspond to bootstrap support >75%. *Tayshaneta* highlighted in green and enlarged at right. **A** *anopica* species-group **B** *myopica* species-group **C** *microps* species-group **D** *sandersi* species-group.

**Figure 7.**
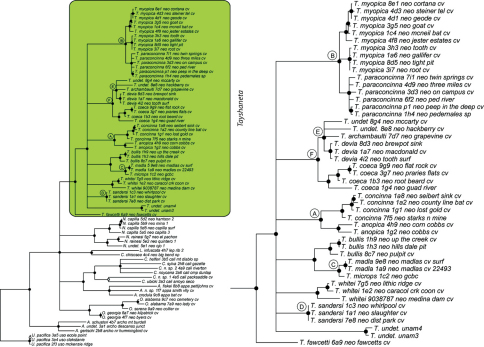
Bayesian gene tree, cytochrome oxidase I (COI) from Ledford et al. (2011). Highlighted and enlarged area indicates *Tayshaneta*. Black nodes correspond to a posterior probability >95%. *Tayshaneta* highlighted in green and enlarged at right. **A** *anopica* species-group **B** *myopica* species-group **C** *microps* species-group **D** *sandersi* species-group.

**Figure 8.**
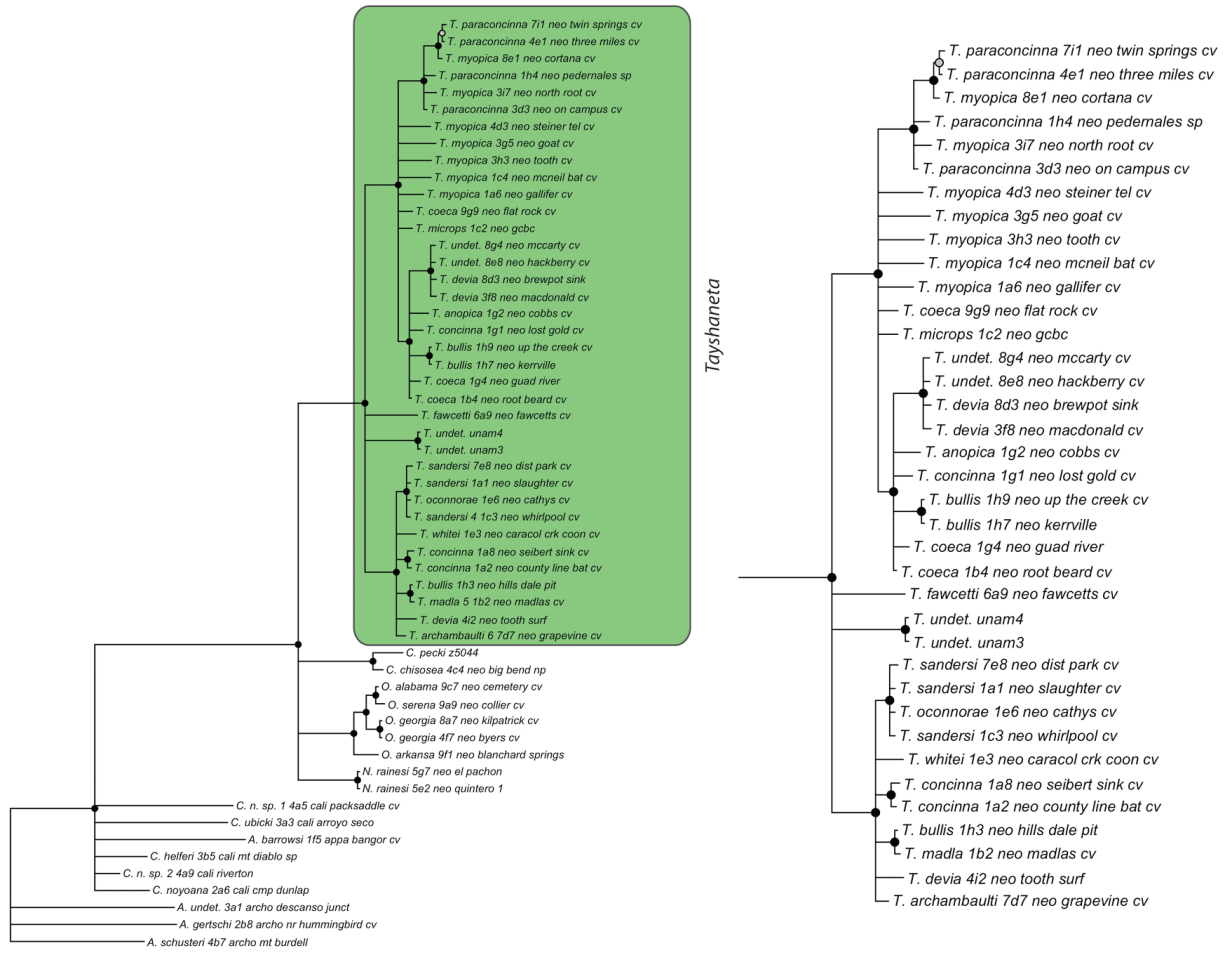
Bayesian gene tree, histone 3 (H3) from Ledford et al. (2011). Highlighted and enlarged area indicates *Tayshaneta*. Black nodes correspond to a posterior probability >95%. *Tayshaneta* highlighted in green and enlarged at right. **A** *anopica* species-group **B** *myopica* species-group **C** *microps* species-group **D** *sandersi* species-group.

**Figure 9.**
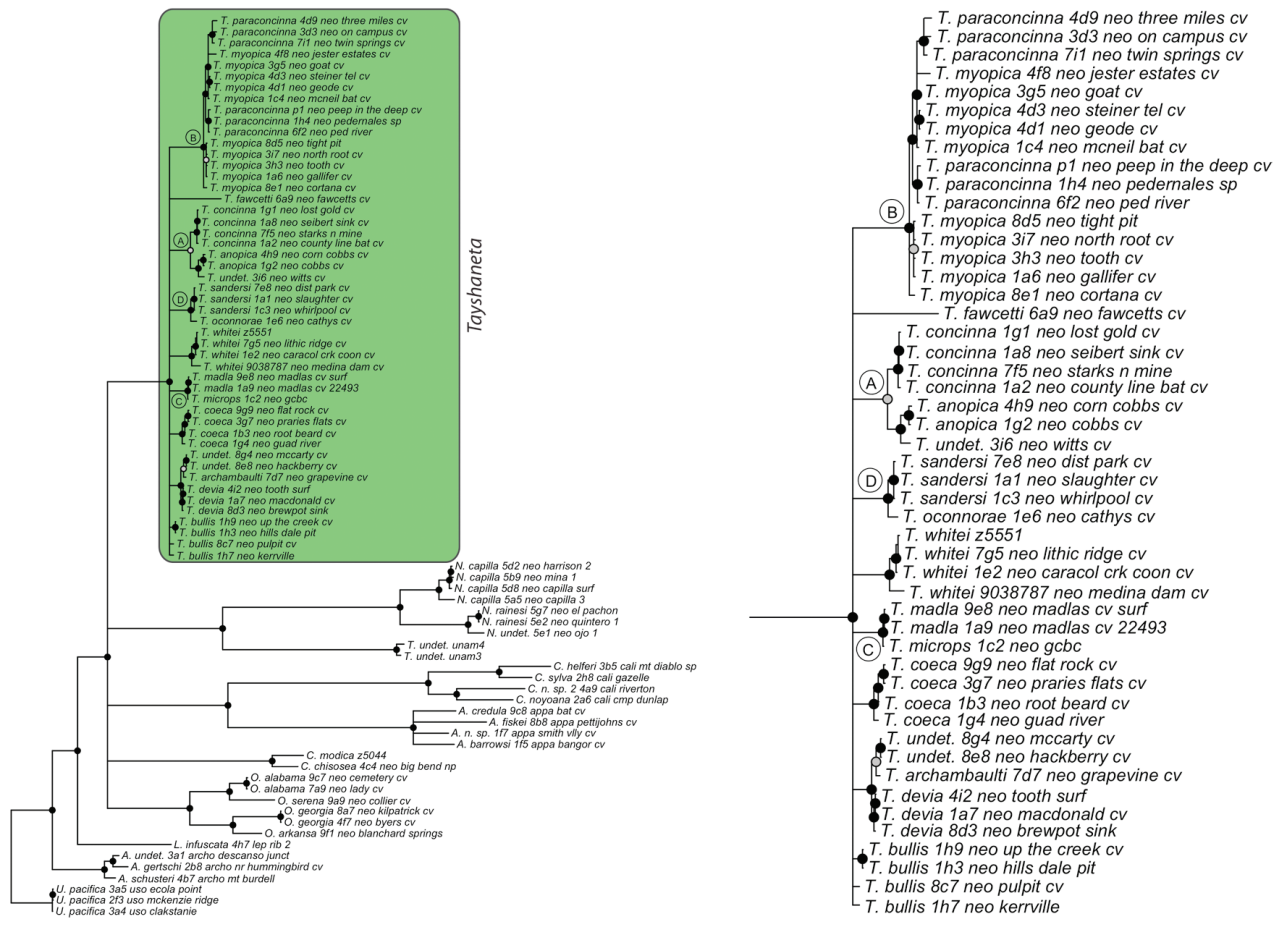
Bayesian gene tree, 28s rDNA (28s) from Ledford et al. (2011). Highlighted and enlarged area indicates *Tayshaneta*. Black nodes correspond to a posterior probability >95%, gray nodes to 75-94%. *Tayshaneta* highlighted in green and enlarged at right. **A** *anopica* species-group **B** *myopica* species-group **C** *microps* species-group **D** *sandersi* species-group.

**Figure 10.**
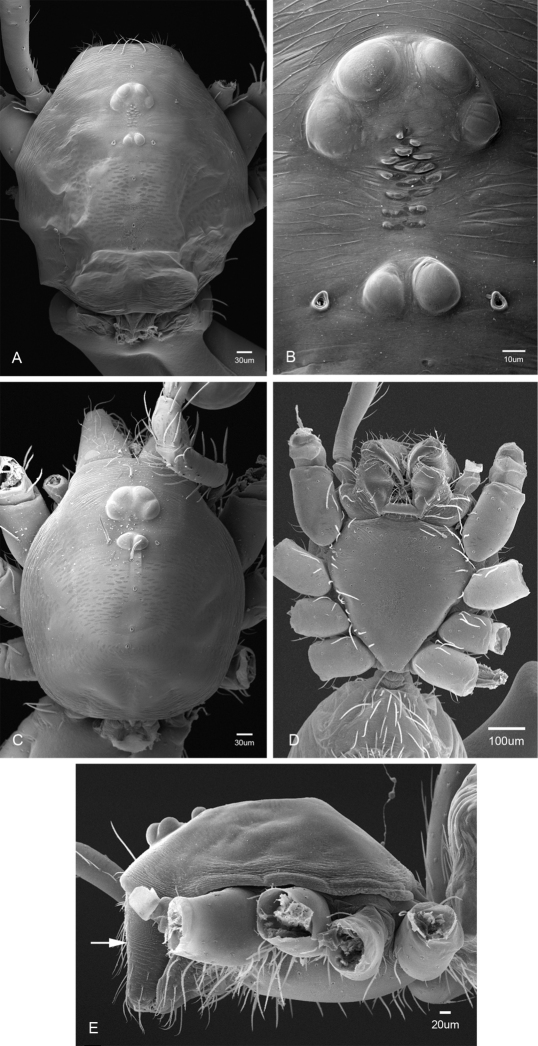
General morphology of *Tayshaneta* species. **A** *Tayshaneta microps* (Gertsch, 1974) male, Government Canyon Bat Cave, carapace dorsal view **B** *Tayshaneta microps* (Gertsch, 1974) male, Government Canyon Bat Cave, ocular area **C** *Tayshaneta coeca* (Chamberlin & Ivie, 1942) male, New Braunfels, carapace dorsal view **D** *Tayshaneta myopica* (Gertsch, 1974) male, Pedernales River, sternum **E** *Tayshaneta myopica* (Gertsch, 1974) male, Pedernales River, carapace lateral view, arrow highlighting stridulatory file.

**Figure 11.**
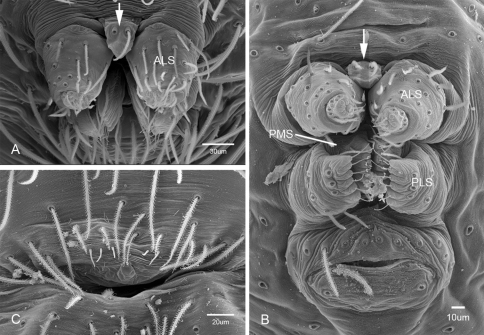
Spinneret morphology for male *Tayshaneta* species. **A** *Tayshaneta myopica* (Gertsch, 1974) male, Pedernales River, arrow to colulus **B** *Tayshaneta devia* (Gertsch, 1974), MacDonald Cave, spinning field **C** *Tayshaneta devia* (Gertsch, 1974), MacDonald Cave, epiandrous spigots.

### Tayshaneta

Ledford & Griswold, in Ledford et al. 2011

http://species-id.net/wiki/Tayshaneta

Leptoneta Simon 1872 (in part); Chamberlin and Ivie 1942 (in part); Gertsch 1974 (in part).
Neoleptoneta Brignoli 1972 (in part); Brignoli 1977 (in part); Platnick 1986 (in part); Cokendolpher and Reddell 2001 (in part); Cokendolpher 2004 (in part); Ledford et al. 2011 (in part).
Tayshaneta Ledford and Griswold, in Ledford et al. 2011: 334-388

#### Type species.

*Leptoneta coeca* Chamberlin & Ivie, 1942.

#### Nomen dubium.

*Leptoneta furtiva* (Gertsch, 1974) is described on on the basis of a single female specimen from Blackwell, Nolan County, Texas. The holotype is in poor condition, missing most of its appendages and genitalia. Efforts to recollect the species at the type locality have proven unsuccessful and the lack of diagnostic features prevents its diagnosis from any other *Tayshaneta* species. *Leptoneta uvaldea* (Gertsch, 1974) was described from Story Cave, Uvalde County, Texas, based on a single female specimen. While the holotype is in good condition, the genitalia are damaged and it cannot be separated from any other *Tayshaneta* species. Furthermore, the type locality, Story Cave, is widely recognized as a lost cave somewhere on the Marneldo Ranch (A. Gluesenkamp, pers. comm.). Given their lack of diagnostic features, both species are declared *nomena dubia* until additional specimens near the type localities can be obtained.

#### Diagnosis.

*Tayshaneta* is separated from all other leptonetids by having males with a recurved to straight retrolateral spine on the palpal tibia (Figs 32A–F) and females with short spermathecal stalks bearing large circular to oval heads (Figs 52-54).

#### Putative synapomorphies.

Species of *Tayshaneta* are united by the unique conformation of the female genitalia, with short spermathecal stalks bearing large heads (Figs 52–54) and the recurved to straight retrolateral spine on the male palpal tibia (Figs 32A–F).

#### Description.

Total length 1.0-1.98. Carapace depigmented to orange-brown; oval and covered by fine, irregular sculpturing which refracts light producing a distinctive iridescence (Figs 10A, C), sparsely setose, length 0.88-1.8× width. Eyes present, reduced, or absent with the PME displaced posteriad of the AEG (Fig. 10B), elevated to flattened in lateral profile (Fig. 10E); chelicerae free and with lateral stridulatory file (Fig. 10E). Sternum triangular to subquadrate (Fig. 10D); abdomen pale yellow to dark brown, lacking distinctive pattern. Colulus triangular, ALS cylindrical, PMS and PLS comb-like, with a linear row of 6–10 aciniform gland spigots (Figs 11A–C). Legs elongate and thin, femur I 1.0–2.26× carapace length; formula I, IV, II, III, covered in fine setae and with few scattered spines; patellar and tibial glands triangular with single, large pores; metatarsus III with ventroapical preening comb. Male palpal tarsus divided or tapering apically, with a middorsal division (Figs 31A–F); tibia with a single recurved to straight retrolateral spine on an elevated base surrounded by elongate setae and 2–4 paddle-shaped setae (Figs 32A–F); palpal bulb oval, longer than wide, with an apically situated embolus (E, Figs 33–51) and an oval prolateral lobe (PL, Figs 33–51); ventral sclerite present (VS, Figs 33, 37, 39–41, 44–50) or absent (Figs 34–36, 38, 42, 43, 51), consisting of a single spine; retrolateral sclerite present or absent, curved and weakly invaginated to oval (RS, Figs 39–41, 49–50) or distinctly separated from the bulb (RS, Fig. 51); tarsal organ circular, shallow and with a pair of receptors. Female genitalia (Figs 52–54) consisting of a single oval to triangular atrium with a pair of lateral spermathecae bearing large, circular (Figs 52A, C–F; 53A–B, D–F; 54A–C, E) to elongate heads (Figs 53C, 54D) that are covered in fine pores.

#### Composition. 

Nineteen species, ten of which are described in this paper:

*Tayshaneta anopica* (Gertsch, 1974), *Tayshaneta archambaulti* sp. n., *Tayshaneta bullis* (Cokendolpher, 2004), *Tayshaneta coeca* (Chamberlin & Ivie, 1942), *Tayshaneta concinna* (Gertsch, 1974), *Tayshaneta devia* (Gertsch, 1974), *Tayshaneta emeraldae* sp. n., *Tayshaneta fawcetti* sp. n., *Tayshaneta grubbsi* sp. n., *Tayshaneta madla* sp. n., *Tayshaneta microps* (Gertsch, 1974), *Tayshaneta myopica* (Gertsch, 1974), *Tayshaneta oconnorae* sp. n., *Tayshaneta paraconcinna* (Cokendolpher & Reddell, 2001), *Tayshaneta sandersi*, sp. n., *Tayshaneta sprousei* sp. n., *Tayshaneta valverdae* (Gertsch, 1974), *Tayshaneta vidrio* sp. n., *Tayshaneta whitei* sp. n.

#### Distribution.

Central to West Texas (Figs 3, 56–61).

#### Tayshaneta anopica

(Gertsch, 1974)

http://species-id.net/wiki/Tayshaneta_anopica

Figs 1A 2D 12A–F 33A–F 52A–B 56

Leptoneta anopica Gertsch 1974: 172.
Neoleptoneta anopica (Gertsch, 1974): Brignoli 1977: 216; Platnick 1986: 6; Platnick 2010.
Tayshaneta anopica (Gertsch, 1974): Ledford et al. 2011.

##### Type data.

Female holotype from Cobb Cave (= Cobb’s Caverns), 15 miles north of Georgetown, Williamson County, Texas, 31-March-1963, J. Reddell, D. McKenzie, 30.78N, 97.73W, (AMNH, examined).

##### Notes.

Cobb Cave is also known as Cobb’s Caverns and is located on the Cobb Ranch in Northern Williamson County (Figs 1A, 55). The general area of Cobb’s Spring has a long history of occupation by Indians who likely discovered the cave thousands of years ago (K. White, pers. comm.). The cave was first reported by the National Speleological Society in 1948 (K. White, pers. comm.) and briefly operated as a commercial cave from 1962 to 1969.

##### Other material examined.

**USA:** Texas: **Williamson County:** Cobb’s Caverns, 15mi. N. of Georgetown, 30-March-2004, M. Warton, 30.78N, 97.73W, 1♂, (TTU); Cobb’s Caverns, 15mi. N. of Georgetown, 12-October-2004, K. White, 30.78N, 97.73W, 1 ♀, (TMM); Cobb’s Caverns, 15mi. N. of Georgetown, 24-November-2004, P. Paquin, 30.78N, 97.73W, 1 ♀, (TMM); Cobb’s Caverns, 15mi. N. of Georgetown, 7-September-2007, P. Paquin, 30.78N, 97.73W, 1♂, (TMM); Cobb’s Caverns, 15mi. N. of Georgetown, 10-December-2009, P. Paquin, C. Crawford, 30.78N, 97.73W, 3 juvs, (TMM); Corn Cobb’s Cave, 17-July-2008, M. Archambault, J. Ledford, P. Paquin, 30.75N, 97.73W, 1 ♀, (TMM); Corn Cobb’s Cave, 15-October-2008, P. Paquin, Parker, Baird, 30.75N, 97.73W, 1 ♀, (TMM); Corn Cobb’s Cave, 31-October-2008, P. Paquin, Crawford, Parker, 30.75N, 97.73W, 1 ♀, (TMM).

##### Diagnosis.

**Figure 12.**
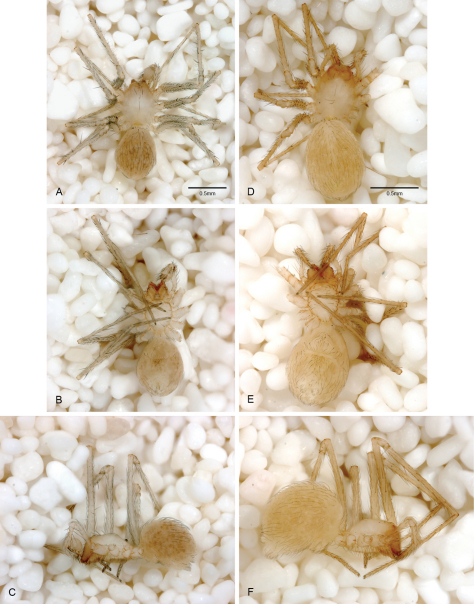
*Tayshaneta anopica* (Gertsch, 1974), Cobb’s Cave, Williamson County, Texas (CASC), habitus. **A** *Tayshaneta anopica* male, dorsal **B** *Tayshaneta anopica* male, ventral **C** *Tayshaneta anopica* male, lateral **D** *Tayshaneta anopica* female, dorsal **E** *Tayshaneta anopica* female, ventral **F** *Tayshaneta anopica* female, lateral.

*Tayshaneta anopica* may be separated from all *Tayshaneta* species that have a ventral sclerite and an undivided male palpal tarsus, except *Tayshaneta concinna*, *Tayshaneta oconnorae* and *Tayshaneta sandersi*, by the following combination of characters: pigmentation and eyes entirely absent (Figs 12A–B); femur I elongate, 1.7–2.3× carapace length; male retrolateral tibial spine thin, sculptured throughout, length 0.50× tarsus length (Fig. 33A); embolus curved distally and with prominent basal tooth (E, Fig. 33D). Separated from *Tayshaneta concinna*, *Tayshaneta oconnorae* and *Tayshaneta sandersi* by having a straight ventral sclerite (VS, Figs 33B, E) and by the unique shape of the embolus (E, Fig. 33D).

##### Description.

Complete description of female in Gertsch (1974: 172). Habitus of female in Figs 12D–F, genitalia as in Fig. 52A and images of egg-sac in Figs 2D, 52B.

**Male** (Cobb’s Caverns). Body length 1.38, carapace 0.62 long, 0.45 wide, length 1.36× width. Carapace depigmented to light brown, eyes absent, sparsely setose (Figs 12A–C). Legs elongate and thin, femur I 2.0× carapace length, covered in fine setae.Palpal tarsus entire, tapering apically; retrolateral tibial spine straight, on shallow base, sculptured throughout, length 0.50× tarsus length (RTS, Fig. 33A). Bulb suboval, length 1.84× width; embolus circular, with prominent basal tooth (E, Fig. 33D), length 1.17× width. Abdomen pale to yellow-brown, without pattern, 0.76 long, 0.54 wide, covered in fine setae.

**Variation** **(*n* = 2).** Total length 1.25–1.38; carapace length 1.19–1.36 × carapace width; femur I length 2.0–2.2 × carapace width.

##### Distribution.

Known only from two caves in Williamson County, Texas (Figs 1A, 56). Cobb’s Caverns is the largest known cave in the area, however, several smaller karst features occur on the property including Corn Cobb’s Cave (K. White, pers. comm.). The records of *Tayshaneta anopica* from Corn Cobb’s Cave suggest that it may be more broadly distributed in the Cobb’s Spring region.

##### Natural History.

An egg-sac for this species was found with a female specimen from Corn Cobb’s Cave (Figs 2D, 52B). The egg-sac was found hanging by a single thread covered with small pebbles and contained two eggs.

#### Tayshaneta archambaulti sp. n.

urn:lsid:zoobank.org:act:0A3E7B18-3AB5-44F9-AF73-4623ABDD215C

http://species-id.net/wiki/Tayshaneta_archambaulti

**Figure 13.**
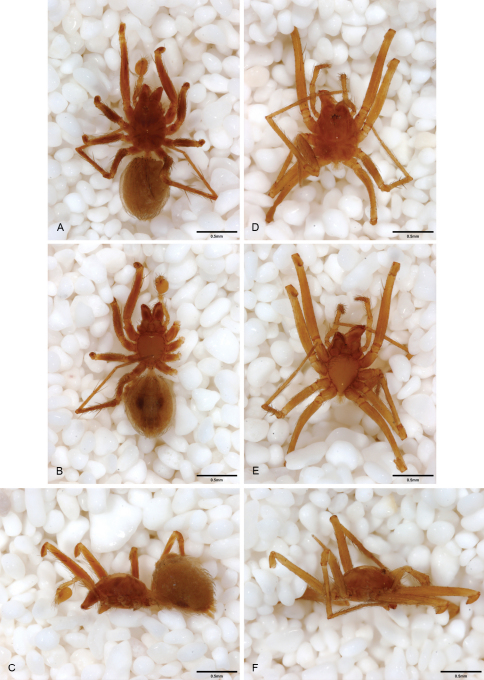
*Tayshaneta archambaulti* sp. n., Burnett Ranch Cave, Hays County, Texas (AMNH), habitus. **A** *Tayshaneta archambaulti* male, dorsal **B** *Tayshaneta archambaulti* male, ventral **C** *Tayshaneta archambaulti* male, lateral **D** *Tayshaneta archambaulti* female, dorsal **E** *Tayshaneta archambaulti* female, ventral **F** *Tayshaneta archambaulti* female, lateral.

Figs 13A–F 34A–F 52C 58

##### Type data.

Male holotype from Grapevine Cave, 7 miles west of Wimberly, Hays County, Texas, 18-Nov-2009, J. Ledford, K. O’Connor, 30.04N, 98.22W, (CASC).

##### Etymology.

This species is named in honor of Martin Archambault, fellow caver and friend who helped collect many leptonetids in Texas and Mexico.

##### Other material examined.

**USA: Hays County:** Burnett Ranch Cave, 7mi. W. of Wimberly, 1982, A. Grubbs, 30.02N, 98.21W, 1♂, 2 ♀, 3 juvs (AMNH); Grapevine Cave, 7mi. W. of Wimberly, 26-May-1989, A. Grubbs, 30.04N, 98.22W, 1♂ (AMNH); Grapevine Cave, 7mi. W. of Wimberly, 23-April-1995, A. Grubbs, Vreeland, 30.04N, 98.22W, 1♂, 1 ♀, 5 juvs, (TMM);Grapevine Cave, 7mi. W. of Wimberly, 18-November-2009, J. Ledford, K. O’Connor, 30.04N, 98.22W, 4♂, 5 ♀, 7 juvs, (TMM).

##### Diagnosis.

*Tayshaneta archambaulti* can be separated from all *Tayshaneta* species that lack a ventral sclerite, except *Tayshaneta coeca* and *Tayshaneta devia*, by the following combination of characters: embolus oval to quadrate, lacking sculpture along its margin (E, Fig. 34D); retrolateral tibial spine short, occupying less than 0.50× the length of the palpal tarsus (RTS, Fig. 34A). Separated from *Tayshaneta devia* by having a retrolateral tibial spine with sculpture along its entire length and from *Tayshaneta coeca* by having the embolus curved distally and extending beyond the apical portion of the bulb (E, Fig. 34E).

##### Description.

**Male** (holotype). Body length 1.35, carapace 0.64 long, 0.56 wide, length 1.13× width. Carapace light brown, eyes reduced, sparsely setose (Figs 13A–F). Legs elongate and thin, femur I 1.5× carapace length, covered in fine setae with few scattered spines.Palpal tarsus entire, tapering apically; retrolateral tibial spine weakly recurved, on weakly elevated base, sculptured throughout, length 0.32× tarsus length (RTS, Fig. 34A). Bulb suboval, length 1.71× width; embolus oval to quadrate (E, Fig. 34D), length 2.0× width. Abdomen pale to yellow-brown, without pattern, 0.70 long, 0.54 wide, covered in fine setae.

**Variation** **(*n* = 4).** Total length 1.34–1.63; carapace length 0.88–1.36 × carapace width; length femur I 1.35–1.65 × carapace width.

**Female** (Grapevine Cave). Body length 1.32, carapace 0.63 long, 0.51 wide, length 1.25× width. Pigmentation and setation same as for male (Figs 13D–F). Legs elongate and thin, femur I 1.34× carapace length, covered in fine setae with few scattered spines.Atrium oval, length 0.51× width, spermathecae with twisted stalks and large, circular heads (Fig. 52C). Abdomen pale to yellow-brown, without pattern, 0.69 long, 0.54 wide, covered in fine setae.

**Variation (*n* = 4).** Total length 1.32–1.72; carapace length 1.20–1.29 × carapace width; length femur I 1.34–1.75 × carapace width.

##### Distribution.

This species is known only from Burnett Ranch Cave and Grapevine Cave in southwestern Hays County (Fig. 58).

##### Natural History.

Individuals for this species were collected throughout Grapevine Cave, however, most specimens were encountered at the base of the cave’s vertical entrance in the twilight area under stones. They were collected in fine sheet webs similar to other *Tayshaneta* species.

#### Tayshaneta bullis

(Cokendolpher, 2004)

http://species-id.net/wiki/Tayshaneta_bullis

**Figure 14.**
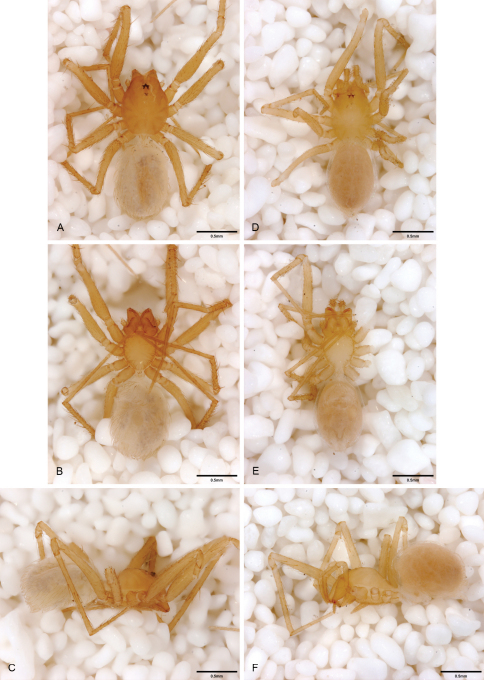
*Tayshaneta bullis* (Cokendolpher, 2004), Up the Creek Cave, Camp Bullis, Bexar County, Texas (male holotype AMNH, female TMM), habitus. **A** *Tayshaneta bullis* male, dorsal **B** *Tayshaneta bullis* male, ventral **C** *Tayshaneta bullis* male, lateral **D** *Tayshaneta bullis* female, dorsal **E** *Tayshaneta bullis* female, ventral **F** *Tayshaneta bullis* female, lateral.

Figs 14A–F 35A–F 52D 59

Neoleptoneta bullis Cokendolpher 2004: 65.
Tayshaneta bullis (Cokendolpher, 2004): Ledford et al. 2011.

##### Type data.

Male holotype from Up the Creek Cave, Camp Bullis, Bexar County, Texas, 10-September-1998, J. Cokendolpher, J. Reddell, J. Krejca, M. Reyes, 29.63N, 98.55W, (AMNH, examined).

##### Notes.

Two female specimens from Hills and Dale’s Pit are tentatively assigned to this species based on the similarity of the female genitalia and by having identical COI and 28s rDNA sequences to specimens collected in Up the Creek Cave.

##### Other material examined.

**USA:** Texas: **Bexar County:** Up the Creek Cave, Camp Bullis, 30-March-1995, J. Cokendolpher, J. Reddell, M. Reyes, J. Krejca 29.63N, 98.55W, 4 ♀, (TMM); Up the Creek Cave, Camp Bullis, 5-October-1995, J. Cokendolpher, J. Reddell, M. Reyes, J. Krejca, 29.63N, 98.55W, 1♂, 3 ♀, (TMM); Up the Creek Cave, Camp Bullis, 5-October-1995, J. Cokendolpher, J. Reddell, M. Reyes, J. Krejca, 29.63N, 98.55W, 1♂, 1 ♀, (CASC); Up the Creek Cave, Camp Bullis, 5-October-1995, J. Cokendolpher, J. Reddell, M. Reyes, J. Krejca, 29.63N, 98.55W, 1♂, 1 ♀, (TTU); Up the Creek Cave, Camp Bullis, 14-November-1995, J. Cokendolpher, J. Reddell, M. Reyes, J. Krejca, 29.63N, 98.55W, 1♂, (TTU); Up the Creek Cave, Camp Bullis, 10-September-1998, J. Krejca, J. Reddell, M. Reyes, 29.63N, 98.55W, 2♂, 2 ♀, 2 juvs, (TTU); Up the Creek Cave, Camp Bullis, 4-November-1998, J. Krejca, J. Reddell, M. Reyes, 29.63N, 98.55W, 2♂, 1 ♀, (TTU); UTSA Area, Hills and Dale’s Pit, 28-October-2000, K. White, H. Bechtol, 29.59N, 98.63W, 1 ♀, (TTU);Up the Creek Cave, Camp Bullis, 16-January-2002, J. Krejca, Engelhard, Schuman, 29.63N, 98.55W, 1♂, 1 ♀, (TTU); Up the Creek Cave, Camp Bullis, 6-August-2008, P. Sprouse, 29.63N, 98.55W, 1 ♀, (TMM).

##### Diagnosis.

*Tayshaneta bullis* can be separated from all other *Tayshaneta* species that lack a ventral sclerite by having an elongate retrolateral tibial spine at least 0.5× the length of the palpal tarsus (RTS, Fig. 35A) and a distinctly quadrate shaped embolus (E, Fig. 35D).

##### Description.

Complete description in Cokendolpher (2004: 65). Habitus of male and female in Figs 14A–F, scanning electron micrographs of male palp in Figs 35A–F and female genitalia in Fig. 52D.

##### Distribution.

Known from two caves in Bexar County, Up the Creek Cave on Camp Bullis and Hills and Dale’s Pit (Fig. 59).

##### Natural History.

Cokendolpher (2004) reported on the shape of the egg-sac for this species along with details on their general biology. The egg-sac was covered in small pebbles or detritus similar to that observed for *Tayshaneta anopica* (Fig. 2D, 53B). Females were observed to retain sperm for several months and the egg-sacs contained few, relatively large eggs.

#### Tayshaneta coeca

(Chamberlin & Ivie, 1942)

http://species-id.net/wiki/Tayshaneta_coeca

**Figure 15.**
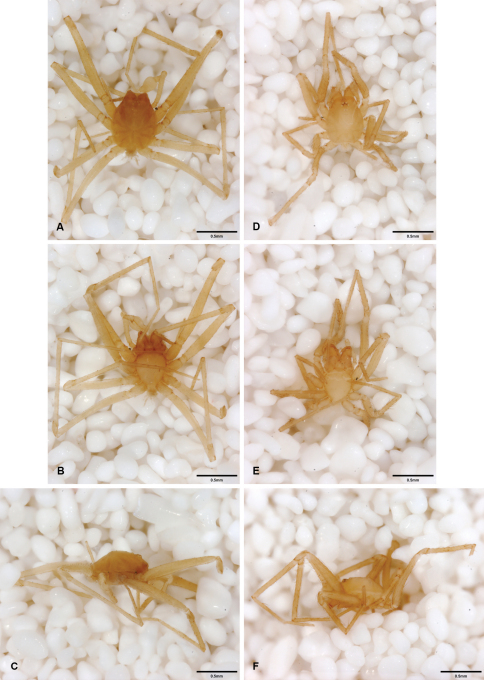
*Tayshaneta coeca* (Chamberlin and Ivie, 1942), Heidrich’s Cave, Comal County, Texas (male holotype, female paratype AMNH), habitus. **A** *Tayshaneta coeca* male, dorsal **B** *Tayshaneta coeca* male, ventral **C** *Tayshaneta coeca* male, lateral **D** *Tayshaneta coeca* female, dorsal **E** *Tayshaneta coeca* female, ventral **F** *Tayshaneta coeca* female, lateral.

Figs 15A–F 36A–F 52E 58

Leptoneta coeca Chamberlin and Ivie 1942: 10; Gertsch 1974: 170.
Neoleptoneta coeca (Chamberlin & Ivie, 1942): Brignoli 1977: 216; Platnick 1986: 7; Cokendolpher, 2004: 64.
Tayshaneta coeca (Chamberlin & Ivie, 1942): Ledford et al. 2011.

##### Type data.

Male holotype from Heidrich’s Cave, New Braunfels, 20-June-1938, Comal County, Texas, 20-June-1938, 29.70N, 98.10W, (AMNH, formerly in the University of Utah collection, examined).

##### Notes.

Heidrich’s Cave was the name used by Chamberlin and Ivie (1942) for Brehmmer Cave in the original description of the species (Reddell and Cokendolpher 2004). Gertsch (1974) considered specimens from Natural Bridge Caverns as conspecific with *Tayshaneta coeca*, however, no illustrations or diagnostic details were provided. Female specimens from Natural Bridges Caverns show similar somatic morphology and genitalia, but cannot be confidently determined in the absence of associated males. While male specimens are reported in Gertsch (1974) they were not located in collections. Given its proximity to the type locality and morphological similarity the specimens are tentatively maintained as conspecific. In several cases, specimens of *Tayshaneta devia* were difficult to separate from *Tayshaneta coeca* except by the fine details of the retrolateral tibial spine and embolus. Given the geographic disjunction between populations in Comal and Williamson Counties, additional sampling is required in these area, especially on the surface, in order to refine species limits.

##### Other material examined.

**USA:** Texas: **Comal County:** Brehmmer Cave (=Heidrich’s Cave), 5mi. W. of New Braunfels, 19-March-1960, W. Gertsch, W. Ivie, Schrammel, 29.70N, 98.10W, 1♂, 1 ♀, (AMNH); Coreth Bat Cave, 28-October-1995, J. Reddell, M. Reyes, 1♂, 1 ♀, 1 juv., (TMM); Coreth Bat Cave, 28-October-1995, J. Reddell, M. Reyes, 1 ♂, 1 ♀, (TTU); Guadeloupe River, 19-November-2004, P. Paquin, 29.81N, 98.17W, 3 ♀, (CASC); Natural Bridge Caverns, 13mi. W. of New Braunfels, 23-February-1963, O. Knox, J. Reddell, M. Reyes, 29.70N, 98.10W, 1 ♀, (TMM); Natural Bridge Caverns, 13mi. W. of New Braunfels, 13-July-1963, J. Reddell, 29.70N, 98.10W, 2 ♀, 3 juvs, (TMM); Natural Bridge Caverns, 13mi. W. New of Braunfels, 23-September-1989, O. Knox, J. Reddell, M. Reyes, 29.70N, 98.10W, 1 ♀, (TMM); Natural Bridge Caverns, 13mi. W. of New Braunfels, 1-March-1990, O. Knox, J. Reddell, M. Reyes, 29.70N, 98.10W, 1 ♀, (TTU); 7mi. W. of New Braunfels, 27-January-1995, A. Grubbs, 3 ♂, 2 ♀, 1 juv. (TMM); **Hays County:** Freeman Crawl, 8-August-2009, P. Sprouse, 1 juv., (TMM); Hackberry Cave, 7-May-2009, P. Sprouse, 30.01N, 97.94W, 1 ♂, 3 juvs, (TMM); Hackberry Cave, 13-October-2009, P. Sprouse, 30.01N, 97.94W, 2 ♂, 2 ♀, 2 juvs, (TMM); McCarty Cave, 14-October-2009, P. Sprouse, 29.85N, 97.99W, 1 ♀, 1 juv., (TMM); McGlothlin Sink, 26-May-1989, A. Grubbs, J. Reddell, M. Reyes, 29.92N, 97.94W, 1 ♂, 1 ♀, 4 juvs, (TMM); Root Beard Cave, 14-March-2005, P. Paquin, 29.97N, 97.98W, 2 ♂, (CASC); Root Beard Cave, 7-June-2009, P. Sprouse, 29.97N, 97.98W, 2 ♂, 1 juv., (TMM); Wiseman’s Sink No. 2, 10mi. W. of San Marcos, 22-April-1995, A. Grubbs, 29.97N, 97.98W, 2 ♀, 4 juvs, (TMM); Wiseman’s Sink, 28-April-1995, A. Grubbs, 29.97N, 97.98W, 1 ♂, (TMM); Wiseman’s Sink, 30-April-1995, A. Grubbs, 29.97N, 97.98W, 4 ♀, 1 juv., (TMM).

##### Diagnosis.

*Tayshaneta coeca* can be separated from other *Tayshaneta* species that lack a ventral sclerite, except *Tayshaneta archambaulti* and *Tayshaneta devia*, by having a short retrolateral tibial spine, occupying less than 0.5× the length of the palpal tarsus (RTS, Fig. 36F) and a rectangular embolus that lacks sculpture along its margin (E, Fig. 36D). Separated from *Tayshaneta devia* by having a retrolateral tibial spine with sculpture along its entire length (RTS, Fig. 36C, F) and from *Tayshaneta archambaulti* by the distinctive shape of the embolus (E, Fig. 36D).

##### Description.

Complete description in Gertsch (1974: 170–171). Habitus of male and female in Figs 15A–F, scanning electron micrographs of male palp in Figs 36A–F and female genitalia in Fig. 52E.

##### Distribution.

Caves and surface localities in Hays and Comal Counties (Fig. 58).

#### Tayshaneta concinna

(Gertsch, 1974)

http://species-id.net/wiki/Tayshaneta_concinna

**Figure 16.**
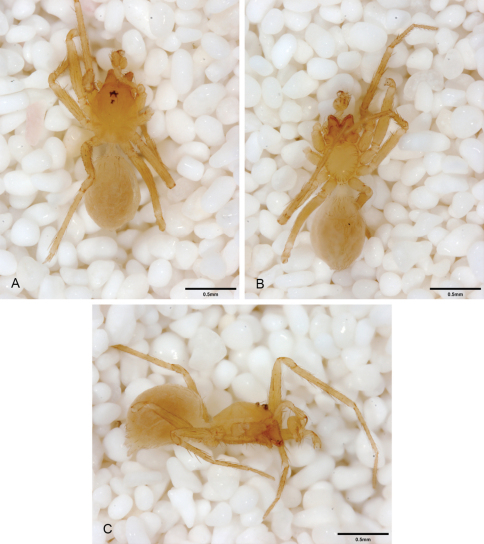
*Tayshaneta concinna* (Gertsch, 1974), Lost Gold Cave, Travis County, Texas (male holotype, AMNH), habitus. **A** *Tayshaneta concinna* male, dorsal **B** *Tayshaneta concinna* male, ventral **C** *Tayshaneta concinna* male, lateral.

Figs 16A–C 37A–F 53F 56

Leptoneta concinna Gertsch 1974: 169.
Neoleptoneta concinna (Gertsch, 1974): Brignoli 1977: 216; Platnick 1986: 7; Cokendolpher and Reddell 2001: 46.
Tayshaneta concinna (Gertsch, 1974): Ledford et al. 2011.

##### Type data.

Male holotype from Lost Gold Cave, 13 miles SW of Austin, Travis County, Texas, 27-May-1963, J. Reddell and B. Frank, 30.26N, 97.81W, (AMNH, examined).

##### Notes.

Gertsch (1974) included a single female specimen from Stark’s North Mine in Travis County as conspecific with *Tayshaneta concinna* although it is unclear which characters he based this decision upon. Stark’s North Mine is a unique feature in the Austin chalk formation and appears to be largely artificial, probably carved out by local residents. Recent inventories at the site have recovered additional *Tayshaneta* specimens, including adult males, which share the genitalic morphology of *Tayshaneta concinna* and are recovered as part of the *concinna* clade (Clade A, Fig. 4). Given the highly disturbed nature of the habitat, it is likely that *Tayshaneta concinna* also occurs on the surface. Although adult males are not available from the populations in Seibert Sink (Travis County) or County Line Bat Cave (Williamson County), molecular analyses support them as close relatives of *Tayshaneta concinna* and they are tentatively assigned to the species pending the discovery of males.

##### Other material examined.

**USA:** Texas: **Travis County:** Lost Gold Cave, 5mi. W. of Austin, 3-March-1985, J. Reddell, M. Reyes, 30.26N, 97.81W, 1 ♀, (AMNH); Lost Gold Cave, 5mi. W. of Austin, 24-November-2004, P. Paquin, 30.26N, 97.81W, 2 ♂, 2 ♀, (CASC); Seibert Sink (=Stinkin Sink), 1-January-1998, M. Sanders, 30.25N, 97.82W, 1 ♀, (TMM); Seibert Sink (=Stinkin Sink), 5-January-1998, M. Sanders, 30.25N, 97.82W, 2 ♀, (TMM, TTU); Stark’s North Mine, 9mi. NNE of Austin, 20-August-1963, W. Russell, 30.38N, 97.67W, 1 ♀, (AMNH); Stark’s North Mine, 9mi. NNE of Austin, 18-September-2000, J. Jenkins, 30.38N, 97.67W, 1 ♀, (TMM); Stark’s North Mine, 9mi. NNE of Austin, 21-November-2009, J. Ledford, P. Paquin, 30.38N, 97.67W, 1 ♂, 3 ♀, 1J (CASC).

##### Diagnosis.

*Tayshaneta concinna* may be separated from all *Tayshaneta* species that have a ventral sclerite, except *Tayshaneta anopica*, *Tayshaneta oconnorae* and T*. sandersi*, by the following combination of characters: male palpal tarsus undivided, tapering apically; male retrolateral tibial spine stout, sculptured throughout, length 0.4× tarsus length (RTS, Fig. 37B); embolus curved distally and with basal tooth (E, Fig. 37D). Separated from *Tayshaneta anopica*, *Tayshaneta oconnorae* and T*. sandersi* by being darkly pigmented with large eyes (Fig. 16A–C) and by the unique shape of the embolus (E, Fig. 37D).

##### Description.

Complete description in Gertsch (1974: 169–170). Habitus of male in Figs 16A–C, scanning electron micrographs of male palp in Figs 37A–F and female genitalia in Fig. 52F.

##### Distribution.

Known from three caves in Travis County, Texas (Fig. 54).

##### Natural History.

Individuals collected in Stark’s North Mine were found in small sheet webs at the base of chalk walls, rotting wood and breakdown material.

#### Tayshaneta devia

(Gertsch, 1974)

http://species-id.net/wiki/Tayshaneta_devia

**Figure 17.**
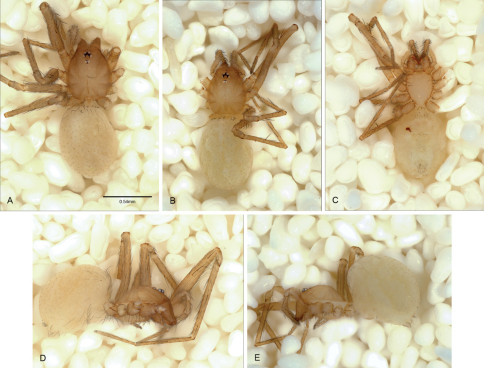
*Tayshaneta devia* (Gertsch, 1974), MacDonald Cave, Travis County, Texas (CASC), habitus. **A** *Tayshaneta devia* male, dorsal **B** *Tayshaneta devia* female, dorsal **C** *Tayshaneta devia* female holotype, ventral **D** *Tayshaneta devia* male, lateral **E** *Tayshaneta devia* female, lateral.

Figs 17A–E 31B 38A–F 53A 56

Leptoneta devia Gertsch 1974: 171.
Neoleptoneta devia (Gertsch, 1974): Brignoli 1977: 216; Platnick 1986: 8.
Tayshaneta devia (Gertsch, 1974): Ledford et al. 2011.

##### Type data.

Female holotype from Shultz Cave, 2mi. E. of Volente, Travis County, Texas, 21-August-1963, B. Russell, 30.43N, 97.86W, (AMNH, examined).

##### Notes.

Shultz Cave is commonly referred to as MacDonald Cave and is located approximately 2.5mi. NE of Volente in Travis County. Although the male for this species was not available to Gertsch (1974), recent inventories of caves in this area have produced the first male specimens and added several new records from nearby caves. Of special interest are records from leaf litter near the entrance of Tooth Cave (type locality for *Tayshaneta myopica*), approximately 2 miles south of MacDonald Cave. Although Gertsch (1974: 171–172) originally described *Tayshaneta devia* as a troglobite based on the type specimen’s reduced eyes and pigment, the discovery of surface populations suggests that the species is a widespread troglophile although some populations may be locally adapted to caves. One record from Williamson County (Village Idiot Cave) is tentative as diagnostic structures on the male palp are partially obscured.

##### Other material examined.

**USA:** Texas: **Travis County:** Brewpot Sink, 19-October-2009, K. O’Connor, 30.41N, 97.85W, 3 ♀, 1 juv., (TMM);Hammett’s Crossing, 14mi. NW of Dripping Springs, 29-September-1994, A. Grubbs, 30.33N, 98.13W, 1 ♂, 2 ♀, (TMM); Highway 71 and Pedernales River, 23mi. W. of Austin, 20-September-1994, A. Grubbs, 30.38N, 98.08W, 2 ♂, 6 ♀, 2 juvs, (TMM); MacDonald Cave, 18-April-1984, Pate, J. Reddell, M. Reyes, 30.43N, 97.86W, 1 ♀, (TMM); MacDonald Cave, 29-April-1989, W. Elliot, J. Reddell, M. Reyes, 30.43N, 97.86W, 3 ♀, (AMNH); MacDonald Cave, 7-January-2005, P. Paquin, 30.43N, 97.86W, 1 ♀, (CASC); MacDonald Cave, September-2008, P. Paquin, 30.43N, 97.86W, 3 ♂, 4 ♀, 4 juvs, (CASC); Stovepipe Cave, 25-October-1990, J. Reddell, M. Reyes, 30.42N, 97.84W, 1 ♂, 1 ♀, (TMM); Stovepipe Cave, 18-September-2009, K. O’Connor, 30.42N, 97.84W, 1 ♀, (TMM); surface above Tooth Cave, 21-November-08, P. Paquin, K. O’Connor, 30.40N, 97.85W, sifting leaf litter, 1 ♂, 3 ♀, (TMM); 9K-2 Cave (=Moonmilk Cave), Spicewood Springs Road, 11-February-95, Elliot, Sprouse, 30.37N, 97.76W, 1 ♂, 2 ♀, 1 juv., (TMM); **Williamson County:** Village Idiot Cave, 31-October-94, Warton, 30.73N, 97.83W, 1 ♂, 1 ♀, (TMM).

##### Diagnosis.

*Tayshaneta devia* may be separated from other *Tayshaneta* species that lack a ventral sclerite, except *Tayshaneta archambaulti* and *Tayshaneta coeca*, by having a short retrolateral tibial spine, occupying less than 0.5× the length of the palpal tarsus (RTS, Fig. 38A) and an apically tapering subquadrate embolus that lacks sculpture along its margin (E, Fig. 38D). Separated from *Tayshaneta archambaulti* and *Tayshaneta coeca* by having a retrolateral tibial spine with a base that lacks distinctive sculpture (RTS, Fig. 31B) and by the unique shape of the embolus (E, Fig. 38D).

##### Description.

Complete description of female in Gertsch (1974: 171–172). Habitus of male and female in Figs 17A–E and female genitalia in Fig. 53A.

**Male** (MacDonald Cave). Body length 1.4, carapace 0.58 long, 0.49 wide, length 1.18× width. Carapace light brown-yellow, sparsely setose; eyes large, ocular area enclosed in a dark pattern. Legs elongate and thin, femur I 1.4× carapace length, covered in fine setae with few scattered spines.Palpal tarsus entire, tapering apically; retrolateral tibial spine on an elevated base, weakly recurved and smooth at its base, length 0.36× tarsus length. Bulb suboval, length 1.70× width; embolus oval, tapering apically (Fig. 38D), length 1.90× width. Abdomen yellow-brown, without pattern, 0.81 long, 0.61 wide, covered in fine setae.

**Variation** **(*n* = 6).** Total length 1.25–1.40; carapace length 1.20–1.52 × carapace width; length femur I 1.0–1.4 × carapace width.

##### Distribution.

Known from caves and surface localities in Travis and Williamson Counties, Texas (Fig. 56).

#### Tayshaneta emeraldae sp. n.

urn:lsid:zoobank.org:act:D8DCEBB5-6DE2-4C79-8E16-C465282BBC99

http://species-id.net/wiki/Tayshaneta_emeraldae

**Figure 18.**
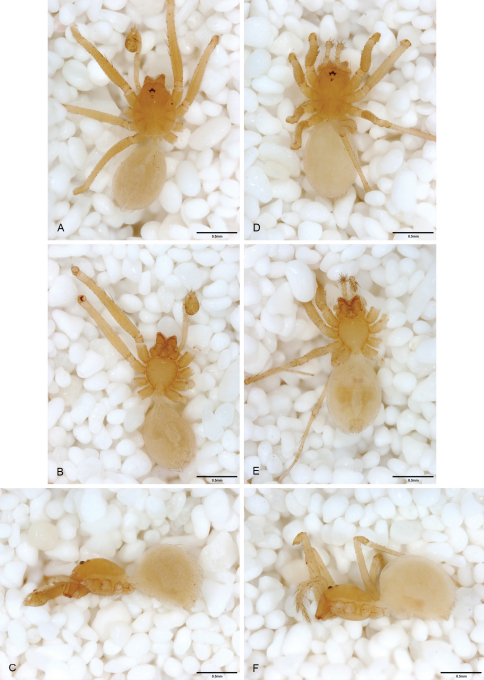
*Tayshaneta emeraldae* sp. n., Emerald Sink, Val Verde County, Texas (AMNH), habitus. **A** male, dorsal **B** male, ventral **C** male, lateral **D** female, dorsal **E** female, ventral **F** female, lateral.

Figs 18A–F 39A–F 53B 60

##### Type data.

Male holotype and female from Emerald Sink, Val Verde County, Texas, 3-November-1984, J. Reddell, M. Reyes, 29.84N, 101.55W, (AMNH).

##### Etymology.

The species name is taken in apposition to the type locality.

##### Diagnosis.

*Tayshaneta emeraldae* sp. n. can be separated from all *Tayshaneta* species, except *Tayshaneta fawcetti*, *Tayshaneta grubbsi*, *Tayshaneta valverdae* and *Tayshaneta vidrio*, by having the following combination of characters: male palpal tarsus divided apically; ventral sclerite short, mesoapically positioned (VS, Fig. 39E); retrolateral sclerite present, pocket-like (RS, Fig. 39D). Separated from *Tayshaneta fawcetti*, *Tayshaneta grubbsi*, *Tayshaneta valverdae* and *Tayshaneta vidrio* by having a distally tapering subquadrate embolus (E, Fig. 39D).

##### Description.

**Male** (holotype). Body length 1.45, carapace 0.63 long, 0.52 wide, length 1.20× width. Carapace orange-brown, sparsely setose; eyes large, ocular area enclosed in a dark pattern (Figs 18A–C). Legs elongate and thin, femur I 1.63× carapace length, covered in fine setae and with few scattered spines.Palpal tarsus divided apically; retrolateral tibial spine smooth at its base, length 0.51× tarsus width. Bulb suboval, length 1.66× width; embolus subquadrate, with weak basal swelling (E, Fig. 39D), length 2.0× width. Ventral sclerite stout, situated mesoapically (VS, Fig. 39E); retrolateral sclerite pocket-like, weakly invaginated (RS, Fig. 39D). Abdomen pale yellow, without pattern, 0.81 long, 0.61 wide, covered in fine setae.

**Female** (Emerald Sink).Body length 1.60, carapace 0.63 long, 0.50 wide, length 1.25× width. Pigmentation and setation same as for male (Figs 18D–F). Legs elongate and thin, femur I 1.4× carapace length, covered in fine setae and with few scattered spines.Atrium trapezoidal, length 0.5× width, spermathecae with twisted stalks and large, circular heads (Fig. 53B). Abdomen pale yellow, without pattern, 0.96 long, 0.65 wide, covered in fine setae.

##### Distribution.

Known only from Emerald Sink, Val Verde County, Texas (Fig. 60).

#### Tayshaneta fawcetti sp. n.

urn:lsid:zoobank.org:act:BA9C2F69-2835-4D9B-96E8-E34FAD673B87

http://species-id.net/wiki/Tayshaneta_fawcetti

Figs 2B 19A–F 31D 40A–F 53C 60

Leptoneta valverdae Gertsch 1974: 174.
Tayshaneta valverdae (Gertsch, 1974): Ledford et al. 2011.

##### Type data.

Male holotype from Fawcett’s Cave, Devil’s River State Natural Area, Val Verde County, Texas, 10-November-2009, J. Ledford, J. Kennedy, M. Sanders, T. Garot, K. Wardlaw, 29.90N, 100.91W, (CASC).

##### Etymology.

The species name is taken in apposition to the type locality and honors the Fawcett family, who owned Fawcett’s Cave and the surrounding Fawcett Ranch prior to its transition as a State Natural Area in 1988.

##### Notes.

Specimens from Fawcett’s Cave were originally considered by Gertsch (1974) as conspecific with *Tayshaneta valverdae* based on similarities in somatic morphology and geography. Male specimens from Fawcett’s Cave were unknown at the time and Gertsch (1974) could not evaluate their genitalic morphology. Recent work at Fawcett’s Cave has recovered a series of male and female specimens which are morphologically distinct from *Tayshaneta valverdae* and appear to not be closely related to other species-groups within *Tayshaneta* (Figs 4–7).

##### Other material examined.

**USA:** Texas: **Val Verde County:** Fawcett’s Cave, 8mi. W. of Loma Alta, 25-March-1961, M. Tandy, 29.90N, 100.91W, 2 juvs, (AMNH); Fawcett’s Cave, 6mi. N. of Del Rio, 10-April-1968, J. Reddell, 29.90N, 100.91W, 1 ♀, 2 juvs, (AMNH); Fawcett’s Cave, 6mi. N. of Del Rio, 10-November-2009, J. Ledford, J. Kennedy, M. Sanders, T. Garot, K. Wardlaw, 29.90N, 100.91W, 6 ♂, 12 ♀, (TMM).

##### Diagnosis.

*Tayshaneta fawcetti* can be separated from all *Tayshaneta* species, except *Tayshaneta emeraldae*, *Tayshaneta grubbsi*, *Tayshaneta valverdae* and *Tayshaneta vidrio*, by having the following combination of characters: male palpal tarsus divided apically (TS, Fig. 31D); ventral sclerite short, mesoapically positioned (VS, Fig. 40E); retrolateral sclerite present, pocket-like (RS, Fig. 40E, F). Separated from *Tayshaneta fawcetti*, *Tayshaneta grubbsi*, *Tayshaneta valverdae* and *Tayshaneta vidrio* by having a distally tapering subquadrate embolus with a distinct basal tooth (E, Fig. 40C, F).

##### Description.

**Figure 19.**
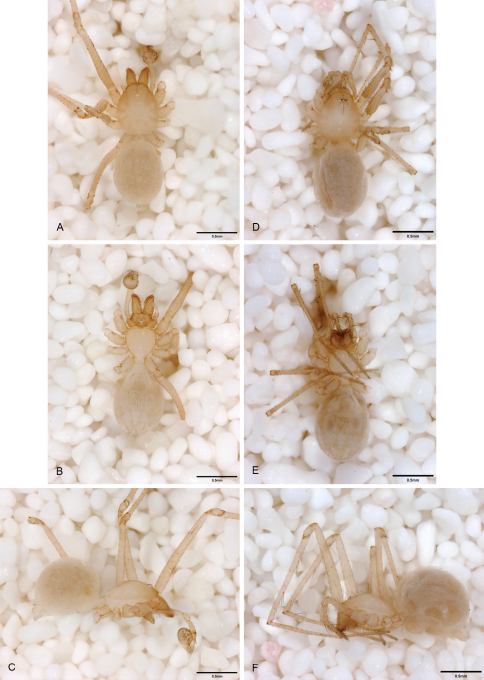
*Tayshaneta fawcetti* sp. n., Fawcett’s Cave, Val Verde County, Texas (CASC), habitus. **A** *Tayshaneta fawcetti* male, dorsal **B** *Tayshaneta fawcetti* male, ventral **C** *Tayshaneta fawcetti* male, lateral **D** *Tayshaneta fawcetti* female, dorsal **E** *Tayshaneta fawcetti* female, ventral **F** *Tayshaneta fawcetti* female, lateral.

**Male** (holotype). Body length 1.56, carapace 0.67 long, 0.58 wide, length 1.15× width. Carapace pale brown, slightly darker surrounding edges, sparsely setose; eyes reduced, ocular area depigmented (Figs 19A–C). Legs elongate and thin, femur I 1.83× carapace length, covered in fine setae and with few scattered spines.Palpal tarsus divided apically (Fig. 31D); retrolateral tibial spine smooth at its base (RTS, Fig. 40D), length 0.38× tarsus width. Bulb suboval, length 1.8× width; embolus rectangular, with basal tooth (E, Fig. 40F), length 1.25× width. Ventral sclerite stout, situated mesoapically (VS, Fig. 40E), retrolateral sclerite pocket-like, weakly invaginated (RS, Figs 40E, F).Abdomen pale brown, without pattern, 0.89 long, 0.67 wide, covered in fine setae.

**Variation** **(*n* = 2).** Total length 1.50–1.56; carapace length 1.15–1.2 × carapace width; length femur I 1.72–1.83 × carapace width.

**Female** (Fawcett’s Cave).Body length 1.4, carapace 0.60 long, 0.50 wide, length 1.17× width. Pigmentation and setation same as for male, except ocular area with a faint dark pattern enclosing the AER (Figs 19D–F). Legs elongate and thin, femur I 1.6× carapace length, covered in fine setae and with few scattered spines.Atrium trapezoidal, length 0.73× width, spermathecae with short twisted stalks and elongate heads (Fig. 53C).Abdomen pale brown, without pattern, 0.80 long, 0.58 wide, covered in fine setae.

**Variation** **(*n* = 2).** Total length 1.25–1.40; carapace length 1.20–1.52 × carapace width; length femur I 1.0–1.4 × carapace width.

##### Distribution.

Known only from Fawcett’s Cave in the Devil’s River State Natural Area, Val Verde County, Texas (Fig. 60).

##### Natural History.

Individuals of *Tayshaneta fawcetti* were photographed during a 2009 expedition to Fawcett’s Cave (Fig. 2B) where they were observed to make fine sheet webs similar to other leptonetid spiders. Male and female pairs were often found in the same web and the egg-sacs were suspended near the web margins. Most specimens were found at the base of the cave’s vertical entrance in twilight under loose rocks and breakdown material.

#### Tayshaneta grubbsi sp. n.

urn:lsid:zoobank.org:act:22A96E29-F1DE-4AEB-8B90-5DF97342F67A

http://species-id.net/wiki/Tayshaneta_grubbsi

**Figure 20.**
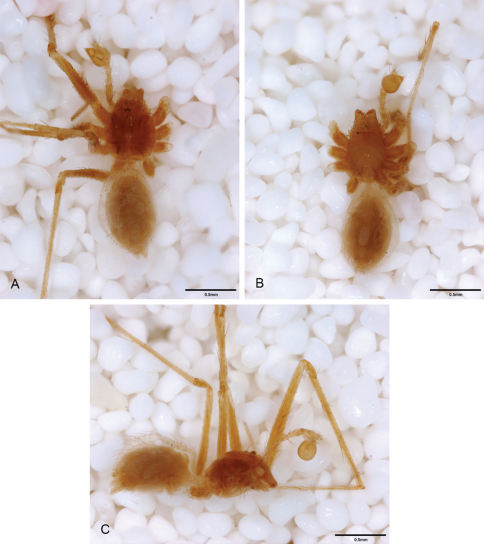
*Tayshaneta grubbsi* sp. n., Litterbarrel Cave, Val Verde County, Texas (AMNH), habitus. Color of specimen significantly darkened due to preservation issues. **A** *Tayshaneta grubbsi* male holotype, dorsal **B** *Tayshaneta grubbsi* male holotype, ventral **C** *Tayshaneta grubbsi* male holotype, lateral.

Figs 20A–C 32E 41A–F 60

##### Type data.

Male holotype from Litterbarrel Cave, 5mi. southeast of Comstock, Val Verde County, Texas, 1-September-1974, S. Sweet, M. Reaka, 29.65N, 101.16W, (AMNH).

##### Etymology.

This species is named in honor of Andy Grubbs, a remarkable collector of several new *Tayshaneta* species throughout Texas.

##### Note.

The coloration of this specimen has likely been affected by its preservation conditions.

**Diagnosis.** *Tayshaneta grubbsi* can be separated from all *Tayshaneta* species, except *Tayshaneta emeraldae*, *Tayshaneta fawcetti*, *Tayshaneta valverdae* and *Tayshaneta vidrio*, by having the following combination of characters: male palpal tarsus divided apically; ventral sclerite short, mesoapically positioned (VS, Fig. 41E); retrolateral sclerite present, pocket-like (RS, Fig. 40A, E). Separated from *Tayshaneta emeraldae* *Tayshaneta fawcetti*, *Tayshaneta valverdae* and *Tayshaneta vidrio* by the unique oval shape of the embolus (Fig. 41D) and the very short ventral sclerite (VS, Fig. 41E).

##### Description.

**Male** (holotype). Body length 1.36, carapace 0.58 long, 0.51 wide, length 1.14× width. Carapace dark orange-brown, sparsely setose; eyes large, ocular area enclosed in a faint dark pattern (Figs 20A–C). Legs elongate and thin, femur I 1.8× carapace length, covered in fine setae and with few scattered spines.Palpal tarsus divided apically; retrolateral tibial spine smooth at its base, length 0.40× tarsus width (RTS, Fig. 41A). Bulb suboval, length 1.8× width; embolus oval, with apical fold (E, Fig. 41D), length 1.8× width. Ventral sclerite short, situated mesoapically (VS, Fig. 41E); retrolateral sclerite pocket-like, weakly invaginated (RS, Figs 41A, E). Abdomen pale yellow, without pattern, 0.81 long, 0.61 wide, covered in fine setae.

##### Distribution.

Known only from Litterbarrel Cave, Val Verde County, Texas (Fig. 60).

#### Tayshaneta madla sp. n.

urn:lsid:zoobank.org:act:8BA1EF6D-5FE0-4842-B814-D92C99DFDBA3

http://species-id.net/wiki/Tayshaneta_madla

**Figure 21.**
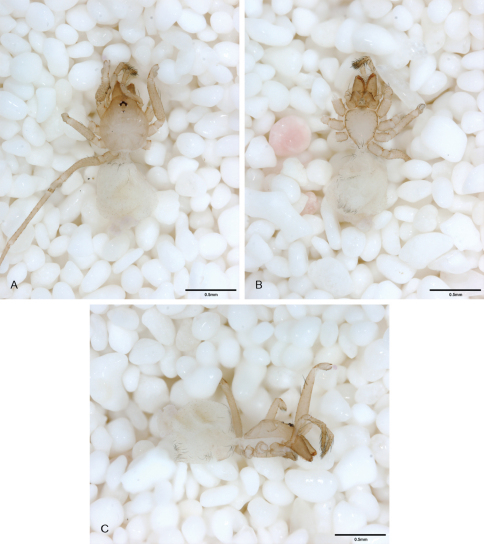
*Tayshaneta madla* sp. n., Madla’s Cave, Bexar County, Texas (CASC), habitus. **A** *Tayshaneta madla* female, dorsal **B** *Tayshaneta madla* female, ventral **C** *Tayshaneta madla* female, lateral.

Figs 21A–C 31F 32F 42A–F 53D 59

##### Type data.

Male holotype from Madla’s Cave, Bexar County, Texas, 18-December-2003, K. White, 29.60N, 98.69W, (CASC).

##### Etymology.

This species name is taken in apposition to the type locality and honors the Madla family, owners of Madla’s Cave and the surrounding property.

##### Notes.

Although the majority of records for this species are from caves, a small series of individuals have been collected from leaf litter near the entrance to Madla’s Cave that are genetically identical to specimens within the cave. The somatic morphology of the species (large, darkly pigmented eyes; Figs 21A–C) coupled with the surface records suggests that it is likely a widespread troglophile.

##### Other material examined.

**USA:** Texas: **Bexar County:** Cave Number 18, 4 miles NE of Helotes, 13-January-1995, A. Grubbs, 29.60N, 98.69W, 1 ♂, 2 ♀, (TMM); Cave Number 189, 4 miles NE of Helotes, 12-January-1995, A. Grubbs, N. Lake, Wade, 4 ♂, 6 ♀, 4J (TTU); Madla’s Cave, 18-December-2003, K. White, 29.60N, 98.69W, 1 ♂, 1 ♀, (TMM); Madla’s Cave, 9-March-2005, P. Paquin, 29.60N, 98.69W, 1 ♂, 1 ♀, 1 juv., (CASC); Madla’s Drop, 8-June-1993, Loftin, J. Reddell, M. Reyes, G. Veni, 29.62N, 98.71W, 1 ♂, (TMM); Scorpion Cave, 1-June-1993, Loftin, J. Reddell, 29.58N, 98.68W, 1 ♂, 6 ♀, 1 juv. (TMM); Young Cave Number 1, 6-September-1993, J. Reddell, M. Reyes, 29.62N, 98.66W, 1 ♂, (TMM).

##### Diagnosis.

*Tayshaneta madla* may be separated from all *Tayshaneta* species, except *Tayshaneta bullis* and *Tayshaneta microps*, by having males with an elongate retrolateral tibial spine (Figs 31F, 32F), more than 0.5× length of the palpal tarsus and lacking a ventral sclerite (Figs 42B, E). Separated from *Tayshaneta bullis* and *Tayshaneta microps* by the unique shape of the embolus with an enlarged basal tooth (E, Fig. 42D, F).

##### Description.

**Male** (holotype). Body length 1.21, carapace 0.58 long, 0.45 wide, length 1.28× width. Carapace pale brown, slightly darker surrounding edges, sparsely setose.

Eyes large, ocular area enclosed in a dark pattern (Figs 21A–C). Legs short and thin, femur I 1.3× carapace length, covered in fine setae and with few scattered spines.Palpal tarsus tapering to weakly divided apically (TS, Fig. 31F); retrolateral tibial spine elongate, sculptured throughout, length 0.58× tarsus length (RTS, Fig. 32F). Bulb suboval, length 1.84× width; embolus oval, with large basal tooth (E, Fig. 42F), length 1.32× width. Abdomen yellow-white, without pattern, 0.63 long, 0.45 wide, covered in fine setae.

**Variation** **(*n* = 5).** Total length 1.14–1.45; carapace length 1.16–1.29 × carapace width; length femur I 1.3–1.71 × carapace width.

**Female** (Madla’s Cave).Body length 1.83, carapace 0.74 long, 0.58 wide, length 1.28× width. Pigmentation and setation same as for male. Legs short and thin, femur I 1.5× carapace length, covered in fine setae and with few scattered spines.Atrium trapezoidal, length 0.56× width, spermathecae with twisted stalks and large, circular heads (Fig. 53D). Abdomen yellow-white, without pattern, 1.09 long, 0.78 wide, covered in fine setae.

**Variation** **(*n* = 3).** Total length 1.45–1.83; carapace length 1.21–1.34 × carapace width; length femur I 1.14–1.51 × carapace width.

##### Distribution.

Known only from Madla’s Cave in Bexar County, Texas (Fig. 59).

#### Tayshaneta microps

(Gertsch, 1974)

http://species-id.net/wiki/Tayshaneta_microps

Figs 1B 10A–B 22A–F 31C 32C 43A–F 53E 59

Leptoneta microps Gertsch 1974: 171–172.
Neoleptoneta microps (Gertsch, 1974): Brignoli 1977: 216; Platnick 1986: 8; Reddell 1988: 34; Cokendolpher 2004: 64; Reddell and Cokendolpher 2004: 86; Platnick 2010.
Tayshaneta microps (Gertsch, 1974): Ledford et al. 2011.

##### Type data.

Female holotype from Government Canyon Bat Cave, 5 miles SW Helotes, Bexar County, Texas, 11-August-1965, J. Reddell, J. Fish (AMNH, examined).

##### Notes.

*Tayshaneta microps* was listed under the Endangered Species Act in 2001 (U. S. Fish and Wildlife, 2010) due to pressure from urbanization in areas surrounding San Antonio, Texas. Two records are currently reported for the species, Government Canyon Bat Cave and Surprise Sink, both of which are in Northern Bexar County. The two specimens from Surprise Sink were examined in detail and while they share reduced eyes similar to *Tayshaneta microps*, both specimens are immature cannot be confirmed as this species in the absence of associated males.

##### Other material examined.

**USA:** Texas: **Bexar County:** Government Canyon Bat Cave, 5 miles SW Helotes, 24-April-1993, J. Reddell, M. Reyes, 29.56N, 98.76W, 1 ♀, (TTU); Government Canyon Bat Cave, 5 miles SW Helotes, 24-May-1993, J. Reddell, M. Reyes, 29.56N, 98.76W, 1 ♀, (TMM); Government Canyon Bat Cave, 5 miles SW Helotes, 24-May-1998, J. Reddell, M. Reyes, 29.56N, 98.76W, 4 ♀, (TMM); Government Canyon Bat Cave, 5 miles SW Helotes, 12-March-2005, P. Paquin, 29.56N, 98.76W, 2 ♂, 2 ♀, 6 juvs, (TMM); Government Canyon Bat Cave, 5 miles SW Helotes, 12-November-2009, J. Ledford, M. Sanders, N. Lake, 29.56N, 98.76W, 1 ♂, (TMM).

##### Diagnosis.

*Tayshaneta microps* may be separated from all *Tayshaneta* species, except *Tayshaneta bullis* and *Tayshaneta madla*, by having males with an elongate retrolateral tibial spine (RTS, Figs 31F, 32F), more than 0.5× length of the palpal tarsus and lacking a ventral sclerite (Figs 42B, E). Separated from *Tayshaneta bullis* and *Tayshaneta madla* by the unique shape of the embolus (Fig. 43D).

##### Description.

**Figure 22.**
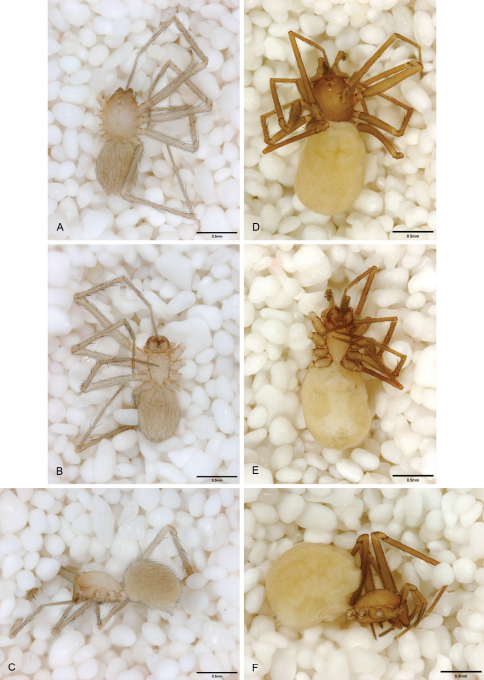
*Tayshaneta microps* (Gertsch, 1974), Government Canyon Bat Cave, Bexar County, Texas (CASC), habitus. **A** *Tayshaneta microps* male, dorsal **B** *Tayshaneta microps* male, ventral **C** *Tayshaneta microps* male, lateral **D** *Tayshaneta microps* female, dorsal **E** *Tayshaneta microps* female, ventral **F** *Tayshaneta microps* female, lateral.

Complete description of female in Gertsch (1974: 171–172). Habitus of male and female in Figs 22A–F, scanning electron micrographs of male genitalia in Figs 43A–F and female genitalia in Fig. 53E.

**Male.** (Government Canyon Bat Cave). Body length 1.27, carapace 0.56 long, 0.47 wide, length 1.19× width. Carapace light brown, sparsely setose; eyes greatly reduced (Figs 10A–B; 22A–C). Legs elongate and thin, femur I 1.64× carapace length, covered in fine setae with few scattered spines. Palpal tarsus entire, tapering apically (Fig. 31C); retrolateral tibial spine elongate, sculptured throughout, length 0.50× tarsus length (Fig. 31C, 32C). Bulb suboval, length 1.76× width; embolus distally oval, curved and with basal tooth (E, Fig. 43D), length 2.0× width. Abdomen light brown, without pattern, 0.70 long, 0.50 wide, covered in fine setae.

**Variation** **(*n* = 6).** Total length 1.25–1.40; carapace length 1.20–1.52 × carapace width; length femur I 1.0–1.4 × carapace width.

##### Natural History.

One adult male specimen was collected for DNA extraction and scanning electron microscopy in November 2009. Although only a single male was found, immature and female specimens were commonly observed in small sheet webs under breakdown material and at the base of walls on opposite sides of the cave entrance.

##### Distribution.

Known only from Government Canyon Bat Cave, Bexar County, Texas (Fig. 59).

#### Tayshaneta myopica

(Gertsch, 1974)

http://species-id.net/wiki/Tayshaneta_myopica

Figs 2A, 2C 23A–F 44A–F 53F 55 57

Leptoneta myopica Gertsch 1974: 168.
Neoleptoneta myopica (Gertsch, 1974): Brignoli 1977: 216; Platnick 1986: 9.
Tayshaneta myopica (Gertsch, 1974): Ledford et al. 2011.

##### Type data.

Male holotype from Tooth Cave, Travis County, Texas, 30-March-1965, J. Reddell, 30.40N, 97.85W, (AMNH, examined).

##### Notes.

*Tayshaneta myopica* was listed under the Endangered Species Act in 1988 (U. S. Fish and Wildlife, 2010) due to its extremely limited distribution in a rapidly urbanizing area outside of Austin, Texas. Recent work has been directed at refining the distribution of the species in order to set recovery goals and several additional localities were discovered during the course of this study (Fig. 56). Of special interest are caves near the type locality which share identical mitochondrial and nuclear DNA haplotypes (Tooth Cave, Root Cave, Gallifer Cave and Tight Pit) suggesting that individuals move between sites.

##### Other material examined.

**USA:** Texas: **Travis County:** Cortaña Cave, 13-September-2006, Shade, Rykwalder, 30.38N, 97.85W, 1 ♀, (TTU); Cortaña Cave, 25-September-2007, P. Sprouse, K. McDermid, 30.38N, 97.85W, 2 ♀, (TTU); Cortaña Cave, 3-October-2007, J. Krejca, P. Sprouse, 30.38N, 97.85W, 1 juv., (TTU); Cortaña Cave, 14-October-2009, K. O’Connor, 30.38N, 97.85W, 1 ♂, 1 ♀, 1 juv., (TMM); Gallifer Cave, 20-April-1991, J. Reddell, M. Reyes, 30.40N, 97.85W, 1 ♂, 3 juvs, (TMM); Gallifer Cave, 7-January-2005, J. Reddell, M. Reyes, 30.40N, 97.85W, 1 ♂, 3 ♀, (TMM); Geode Cave, 11-August-1993, W. Elliot, 30.39N, 97.86W, 1 ♀, (TMM); Geode Cave, 21-July-1994, W. Elliot, P. Sprouse, 30.39N, 97.86W, 2 ♂, 7 ♀, 2 juvs, (TMM); Geode Cave, 11-August-1994, W. Elliot, 30.39N, 97.86W, 2 ♂, 4 ♀, 1 juv., (TMM); Geode Cave, 13-September-1994, W. Elliot, 30.39N, 97.86W, 1 ♀, (TMM); Geode Cave, 18-July-2007, K. O’Connor, 30.39N, 97.86W, 1 ♂, 1 ♀, 1 juv., (TMM); Geode Cave, 16-October-2007, Myers, 30.39N, 97.86W, 2 juvs, (TTU); Geode Cave, 31-October-2007, J. Krejca, 30.39N, 97.86W, 2 ♂, 4 ♀, 8 juvs, (TTU); Jester Estate’s Cave, 14-March-2006, M. Sanders, 30.39N, 97.79W, 1 ♀, 1 juv., (TMM); Jester Estate’s Cave, 18-September-2009, M. Sanders, 30.39N, 97.79W, 1 ♂, 3 ♀, 1 juv., (TMM); McNeil Bat Cave, 2-March-1986, J. Reddell, M. Reyes, 30.09N, 97.72W, 1 ♂, 1 ♀, (AMNH); McNeil Bat Cave, 11-March-2005, P. Paquin, 30.45N, 97.72W, 1 ♀, 1 juv., (CASC); New Comanche Trail Cave, 11-January-1989, J. Reddell, M. Reyes, 30.39N, 97.86W, 2 ♀, (AMNH); New Comanche Trail Cave, 26-January-1989, J. Reddell, M. Reyes, 30.39N, 97.86W, 2 ♂, 1 ♀, 2 juvs, (AMNH); New Comanche Trail Cave, 16-October-2007, J. Krejca, 30.39N, 97.86W, 1 ♂, 3 ♀, (TTU); New Comanche Trail Cave, 23-October-2007, P. Sprouse, 30.39N, 97.86W, 2 juvs, (TMM); Root Cave, 1-September-2008, P. Paquin, 30.40N, 97.85W, 1 juv., (TMM); Steiner Telephone Pole Cave, 17-July-2008, J. Ledford, P. Paquin, M. Archambault, 30.39N, 97.86W, 1 ♂, 3 ♀, 1 juv., (CASC); Tight Pit, 14-October-2009, K. O’Connor, 1 ♀, (TMM); Tooth Cave, 25-February-1963, D. McKenzie, J. Reddell, 30.40N, 97.85W, 1 ♂, 3 ♀, (AMNH); Tooth Cave, 5-March-1964, J. Reddell, D. McKenzie, T. Phillips, 30.40N, 97.85W, 1 ♀, (AMNH); Tooth Cave, 9-June-1967, D. McKenzie, J. Reddell, 30.40N, 97.85W, 2 ♂, 4 ♀, (AMNH); Tooth Cave, 8-March-1968, J. Reddell, W. Russell, S. Fowler, 30.40N, 97.85W, 1 ♀, (AMNH); Tooth Cave, 19-July-1970, D. McKenzie, J. Reddell, 30.40N, 97.85W, 4 ♀, (AMNH); Tooth Cave, 24-May-1992, J. Reddell, 30.40N, 97.85W, 1 juv., (AMNH); Tooth Cave, 1-September-2008, P. Paquin, 30.40N, 97.85W, 2 ♂, 8 ♀, 6 juvs, (TMM); **Williamson County:** Goat Cave, 1-September-2008, P. Paquin, 30.49N, 97.71W, 1 ♀, 2 juvs, (CASC).

##### Diagnosis.

*Tayshaneta myopica* may be separated from all other *Tayshaneta* species, except *Tayshaneta paraconcinna*, by having an elongate ventral sclerite (VS, Fig. 44E) and a broad spoon shaped embolus (E, Fig. 44D). Separated from *Tayshaneta paraconcinna* by having the embolus sharply projecting ventrally (Fig. 44D) and having a recurved, but not sickle-shaped, retrolateral tibial spine (RTS, Fig. 44A).

##### Description.

**Figure 23.**
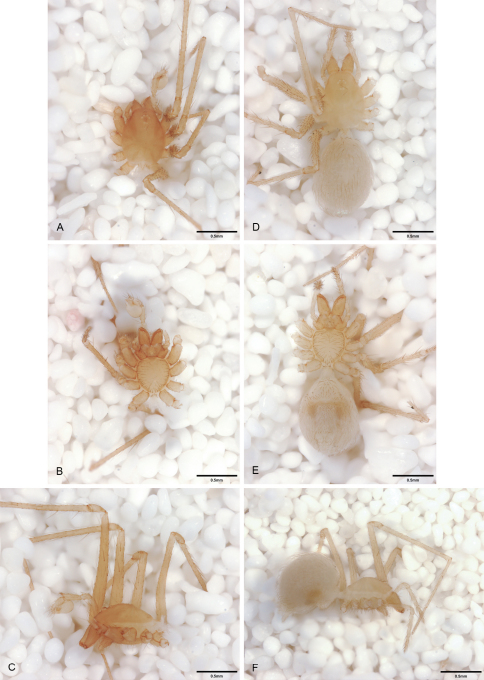
*Tayshaneta myopica* (Gertsch, 1974), Tooth Cave, Travis County, Texas (CASC), habitus. **A** *Tayshaneta myopica* male, dorsal **B** *Tayshaneta myopica* male, ventral **C** *Tayshaneta myopica* male, lateral **D** *Tayshaneta myopica* female, dorsal **E** *Tayshaneta myopica* female, ventral **F** *Tayshaneta myopica* female, lateral.

Complete description in Gertsch (1974: 169–170). Habitus of male and female in Figs 23A–F, scanning electron micrographs of male palp in Figs 44A–F and female genitalia in Fig. 53F.

##### Distribution.

Known from caves in Travis and Williamson Counties, Texas (Fig. 57).

##### Natural History.

Individuals in Geode Cave and Tooth Cave were observed suspended beneath sheet webs at the bases of stable rocks and breakdown material (Figs 2A, 2C). When disturbed, individuals would drop from their webs and fold their legs in a protective posture similar to that reported for *Calileptoneta* (Ledford, 2004).

#### Tayshaneta oconnorae sp. n.

urn:lsid:zoobank.org:act:1D28692E-D4C1-454F-A2FA-6AD0AF1AE2FA

http://species-id.net/wiki/Tayshaneta_oconnorae

Figs 24A–C 45A–F 58

##### Type data.

Male holotype from Fern Cave, Hays County, Texas, 26-May-1989, A. Grubbs, J. Reddell, M. Reyes, 29.97N, 97.99W, (AMNH).

##### Etymology.

This species is named in honor of Kathleen O’ Connor, fellow caver and biologist who helped collect many exciting *Tayshaneta* specimens.

##### Notes.

A single adult male collected from Cathy’s Cave, Hays County, Texas shares the genitalic morphology of *Tayshaneta oconnori* but was damaged during examination and only the right palp remains. The specimen was highly troglobitic and is tentatively assigned to *Tayshaneta oconnori* until additional specimens can be collected.

##### Other material examined.

**USA:** Texas: **Hays County:** Cathy’s Cave, 15-March-2005, P. Paquin, 29.90N, 98.08W, 1 ♂, (CASC).

##### Diagnosis.

**Figure 24.**
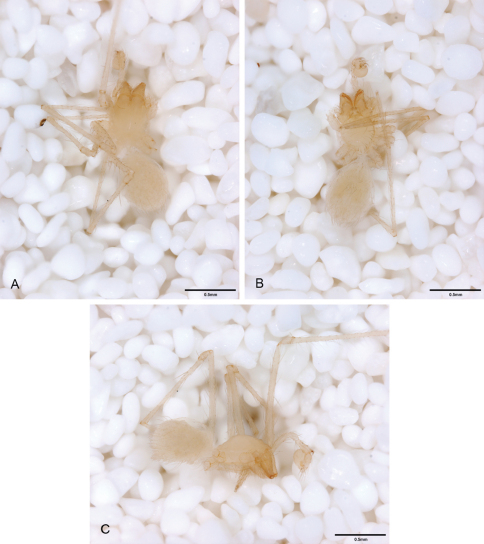
*Tayshaneta oconnorae* sp. n., Fern Cave, Hays County, Texas (AMNH), habitus. **A** *Tayshaneta oconnorae* male holotype, dorsal **B** *Tayshaneta oconnorae* male holotype, ventral **C** *Tayshaneta oconnorae* male holotype, lateral.

*Tayshaneta oconnori* may be separated from all *Tayshaneta* species, except *Tayshaneta anopica* and *Tayshaneta sandersi*, by having the following combination of characters: pigmentation and eyes entirely absent (Figs 24A–C); legs extremely long and thin, femur I 1.8–1.9× carapace length; embolus with a distinctive apical bifurcation (E, Fig. 45D). Separated from *Tayshaneta anopica* and *Tayshaneta sandersi* by having the ventral sclerite straight and short, not extending past the base of the embolus (VS, Fig. 45E) and by the unique shape of the embolus (E, Fig. 45D).

##### Description.

**Male** (holotype). Body length 1.1, carapace 0.52 long, 0.40 wide, length 1.31× width. Carapace depigmented, sparsely setose; eyes absent (Figs 24A–C). Legs elongate and thin, femur I 1.93× carapace length, covered in fine setae and with few scattered spines. Palpal tarsus entire, tapering apically; retrolateral tibial spine recurved, sculptured throughout, length 0.40× tarsus length. Bulb suboval, length 1.66× width; embolus oval, bifurcate apically (E, Fig. 45D), length 1.2× width. Abdomen depigmented, without pattern, 0.58 long, 0.45 wide, covered in fine setae.

##### Distribution.

Known only from two caves in Hays County, Texas (Fig. 58).

#### Tayshaneta paraconcinna

(Cokendolpher & Reddell, 2001)

http://species-id.net/wiki/Tayshaneta_paraconcinna

**Figure 25.**
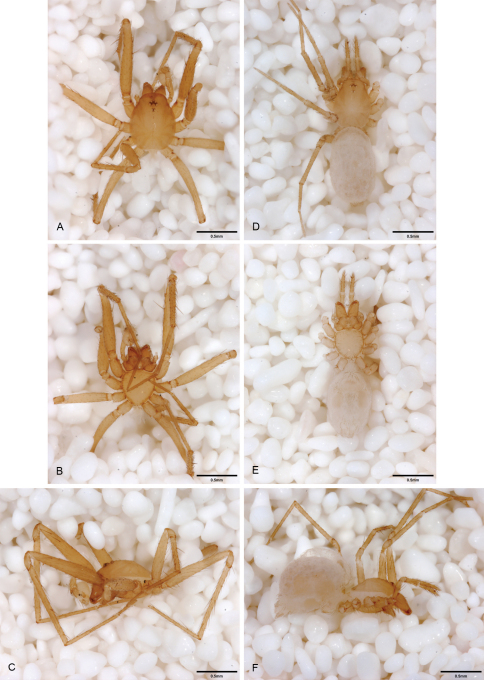
*Tayshaneta paraconcinna* (Cokendolpher & Reddell, 2001), Camp 6 Cave Number 1, Fort Hood, Bell County, Texas (TMM), habitus. **A** *Tayshaneta paraconcinna* male, dorsal **B** *Tayshaneta paraconcinna* male, ventral **C** *Tayshaneta paraconcinna* male, lateral **D** *Tayshaneta paraconcinna* female, dorsal **E** *Tayshaneta paraconcinna* female, ventral **F** *Tayshaneta paraconcinna* female, lateral.

Figs 25A–F 31A 32A 46A–F 54A 57

Neoleptoneta paraconcinna Cokendolpher and Reddell 2001: 46; Platnick 2010.
Tayshaneta paraconcinna (Cokendolpher and Reddell 2001): Ledford et al. 2011.

##### Type data.

Male holotype from Peep in the Deep Cave, Fort Hood, Bell County, Texas, 8-May-1998, J. Reddell, M. Reyes, 31.20N, 97.51W, (AMNH).

##### Other material examined.

**USA:** Texas: **Bell County:** Camp 6 Cave Number 1, Fort Hood, 5-April-1999, J. Reddell, M. Reyes, 31.20N, 97.51W, 1 ♂, (TMM); Figure 8 Cave, Fort Hood, 20-April-1998, Graves, J. Reddell, M. Reyes, 31.20N, 97.51W, 1 ♀, (TMM);Hidden Pit Cave, Fort Hood, 18-August-2003, Perkins, J. Reddell, M. Reyes, 31.20N, 97.51W, 1 ♀, 3 juvs, (TMM);Hidden Pit Cave, Fort Hood, 21-March-2004, J. Fant, J. Reddell, M. Reyes, 31.20N, 97.51W, 1 ♀, 3 juvs, (TMM);Peep in the Deep Cave, Fort Hood, 8-May-1998, J. Reddell, M. Reyes, 31.20N, 97.51W, 1 ♀, (TMM);Peep in the Deep Cave, Fort Hood, 21-April-1998, J. Reddell, M. Reyes, 31.20N, 97.51W, 2 ♀, (TMM);Peep in the Deep Cave, Fort Hood, 3-November-1998, J. Reddell, M. Reyes, 31.20N, 97.51W, 1 ♀, (TMM);Peep in the Deep Cave, Fort Hood, 8-June-2010, J. Fant, 31.20N, 97.51W, 3 ♀, 1 juv., (TMM); Talking Crows Cave, Fort Hood, 20-May-1998, Graves, J. Reddell, M. Reyes, 31.20N, 97.51W, 1 juv., (TMM); **Blanco County:** Flat Creek Ranch, 12miles E. Johnson City, 28-May-1995, A. Grubbs, 30.27N, 98.21W, 1 ♂, 1 ♀, (TTU); Pedernales State Park, 17-December-2003, P. Paquin, W. Wytrykush, 30.30N, 98.26W, 1 ♂, 1 ♀, (CASC); **Burnet County:** Doublehorn Creek and Highway 71, 4.9 miles SE Marble Falls, 20-January-1995, A. Grubbs, 30.49N, 98.23W, 3 ♂, 5 ♀, 4 juvs, (TMM); Moon Rocks Ranch, 5 miles W. Spicewood, A. Grubbs, Waid, 30.47N, 98.24W, 1 ♂, (TMM); County Road 404, 5 miles W. Spicewood, site #1, 29-November-1994, A. Grubbs, 30.47N, 98.24W, 1 ♂, 3 ♀, (TTU); **Travis County:** Hwy. 71 and Pedernales River, 23mi. W. Austin, 3-October-1994, Grubbs, 30.38N, 98.08W, 1 ♂, 1 ♀, (TMM); Hwy. 71 and Pedernales River, 23mi. W. Austin, 17-November-2009, P. Paquin, J. Ledford, 30.38N, 98.08W, 18 ♂, 14 ♀, 4 juvs, (CASC); **Williamson County:** Fissure F-8, The Sanctuary, 3.3mi. W. Georgetown, A. Grubbs, 1 ♂, (TMM); Lizard’s Lounge Cave, F-11, 3.3mi. W. Georgetown, 14-Aug-2003, Fant, A. Grubbs, 30.62N, 97.73W, 2 ♂, 2 ♀, (TMM); On Campus Cave, 1-Sep-2008, P. Paquin, 30.61N, 97.69W, 1 ♀, (CASC); Salt Lick Cave, the Sanctuary, 3.3mi. W. Georgetown, A. Grubbs, 30.62N, 97.73W, 4 ♂, 3 ♀, 1 juv. (TMM); Scoot Over Cave, 1-April-1994, Warton, 30.48N, 97.72W, 2 ♂, 1 ♀, 1 juv., (TMM); Serta Cave, 1-April-1994, Warton, 30.48N, 97.72W, 1 ♀, 1 juv., (TMM);Short Stack Cave, 1-April-1994, Warton, 30.53N, 97.70W, 1 ♂, 2 ♀, (TTU);Short Stack Cave, 19-May-1995, J. Reddell, 30.53N, 97.70W, 1 ♂, 2 ♀, 1 juv., (TMM);Three Miles Cave (=Three Mile Bat Cave), 17-Aug-2008, P. Paquin, J. Ledford, M. Archambault, 30.63N, 97.73W, 6 ♀, 2 juvs, (CASC);Twin Springs Cave (=Whitney West Cave), 10.xii.2009, P. Paquin, 30.69N, 97.78W, 1 ♂, 1 juv., (CASC).

##### Diagnosis.

*Tayshaneta paraconcinna* may be separated from all other *Tayshaneta* species, except *Tayshaneta myopica*, by having an elongate ventral sclerite (VS, Fig. 46E) and a broad spoon shaped embolus (E, Fig. 46D). Separated from *Tayshaneta myopica* by having the embolus projecting anteriorly (E, Fig. 46D) and having a sharply recurved, sickle-shaped, retrolateral tibial spine (RTS, Fig. 46A).

##### Description.

Complete description in Cokendolpher (2001: 46). Habitus of male and female in Figs 25A–F, scanning electron micrographs of male palp in Figs 46A–F and female genitalia in Fig. 54A.

##### Distribution.

Caves of Fort Hood, Bell County, Texas and surface localities in Blanco, Burnett, Travis and Williamson Counties, Texas (Fig. 57).

#### Tayshaneta sandersi sp. n.

urn:lsid:zoobank.org:act:3722907F-76E3-475B-8A0A-77B574BC8A8E

http://species-id.net/wiki/Tayshaneta_sandersi

Figs 1C 26A–C 47A–F 54B 56

##### Type data.

Female holotype from District Park Cave, Travis County, Texas, 19-November-2009, J. Ledford, M. Sanders, 30.21N, 97.85W, (CASC).

##### Etymology.

This species is named in honor of Mark Sanders, fellow caver, biologist, and collector of several *Tayshaneta* species in Texas.

##### Notes. 

The only known adult male for *Tayshaneta sandersi* is from Whirlpool Cave and is missing most of its appendages and the carapace. Individuals from District Park Cave, Slaughter Creek Cave and Whirlpool Cave are genetically identical suggesting that the species may occur more broadly in the Onion Creek watershed of Barton Springs.

##### Other material examined.

**USA:** Texas: **Travis County:** Slaughter Creek Cave, 6-January-2005, P. Paquin, 30.19N, 97.87W, 1J, (CASC); Whirlpool Cave, 2-March-2005, P. Paquin, 1M, (TMM); District Park Cave, 19-November-2009, J. Ledford, M. Sanders, 30.21N, 97.85W, 1 ♀, 2J, (TMM).

##### Diagnosis.

**Figure 26.**
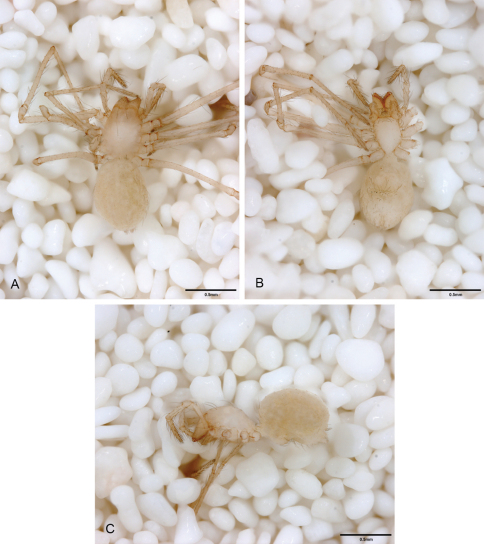
*Tayshaneta sandersi* sp. n., District Park Cave, Travis County, Texas (CASC), habitus. **A** *Tayshaneta sandersi* female holotype, dorsal **B** *Tayshaneta sandersi* female holotype, ventral **C** *Tayshaneta sandersi* female holotype, lateral.

*Tayshaneta sandersi* may be separated from all *Tayshaneta* species, except *Tayshaneta anopica* and *Tayshaneta oconnori*, by having the following combination of characters: pigmentation and eyes entirely absent (Figs 26A–C); legs extremely long and thin, femur I 1.8–1.9× carapace length; embolus with a distinctive apical bifurcation (E, Fig. 47D). Separated from *Tayshaneta anopica* and *Tayshaneta oconnori* by having the ventral sclerite curved prolaterally (VS, Fig. 47E) and by the unique shape of the embolus (E, Fig. 47D).

##### Description.

**Male** (Whirlpool Cave, genitalia only). Palpal tarsus entire, tapering apically; retrolateral tibial spine recurved, sculptured throughout, length 0.38× tarsus length (Fig. 47A). Bulb suboval, length 1.97× width; embolus suboval, bifurcate apically (E, Fig. 47D), length 1.6× width. Ventral sclerite elongate, curved prolaterally (VS, Fig. 47E).

**Female** (holotype).Body length 1.2, carapace 0.58 long, 0.41 wide, length 1.39× width. Carapace depigmented, sparsely setose; eyes absent. Legs elongate and thin, femur I 1.90× carapace length, covered in fine setae and with few scattered spines.Atrium suboval, length 0.41× width, spermathecae with twisted stalks and large, circular heads (Fig. 54B). Abdomen depigmented, 0.61 long, 0.49 wide, covered in fine setae.

##### Natural History.

Three individuals were found deep in District Park Cave in fine sheet webs under loose rocks. The single male individual was found wandering among loose rocks in Whirlpool Cave.

##### Distribution.

Known from three caves in Travis County, Texas (Fig. 56).

#### Tayshaneta sprousei sp. n.

urn:lsid:zoobank.org:act:9395BD71-ADF6-4F41-B8BC-8CD45F88A016

http://species-id.net/wiki/Tayshaneta_sprousei

Figs 27A–C 48A–F 59

##### Type data.

Male holotype from Constant Sorrow Cave, Camp Bullis, Bexar County, Texas, 6-March-2001, G. Veni, 29.63N, 98.58W, (AMNH).

##### Etymology.

This species is named in honor of Peter Sprouse, fellow caver, biologist and collector of several *Tayshaneta* species in Texas caves.

##### Other material examined.

**USA:** Texas: **Bexar County:** Breached Dam Cave, 4-October-1995, J. Reddell, M. Reyes, 1 juv., (TMM);Breached Dam Cave, 1-November-2000, J. Reddell, M. Reyes, 1 ♂, (TMM).

##### Diagnosis.

*Tayshaneta sprousei* may be separated from all *Tayshaneta* species by having the following combination of characters: male palpal tarsus undivided, tapering apically (Fig. 48C); retrolateral tibial spine elongate, 0.5× length of palpal tarsus; embolus oval, smooth along margins (E, Fig. 48D); ventral sclerite short, less than the width of the embolus (Fig. 48B, D–F).

##### Description.

**Figure 27.**
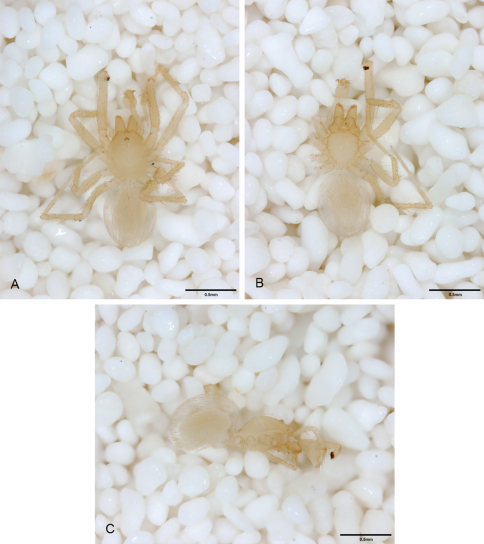
*Tayshaneta sprousei* sp. n., Constant Sorrow Cave, Camp Bullis, Bexar County, Texas (TMM), habitus. **A** *Tayshaneta sprousei* male holotype, dorsal **B** *Tayshaneta sprousei* male holotype, ventral **C** *Tayshaneta sprousei* male holotype, lateral.

**Male** (holotype). Body length 1.1, carapace 0.47 long, 0.40 wide, length 1.18× width. Carapace pale yellow, sparsely setose; eyes surrounded by faint dark markings (Figs 27A–C). Legs elongate and thin, femur I 1.19× carapace length, covered in fine setae and with few scattered spines. Palpal tarsus entire, tapering apically (TS, Fig. 48C); retrolateral tibial spine straight, sculptured throughout, length 0.49× tarsus length. Bulb suboval, length 1.52× width; embolus oval, smooth along margins (E, Fig. 48D), length 2.0× width; ventral sclerite short, less than embolus width (VS, Figs 48B, D–F). Abdomen pale yellow, without pattern, 0.63 long, 0.50 wide, covered in fine setae.

**Variation** **(*n* = 2).** Total length 1.10–1.12; carapace length 1.18–1.36 × carapace width; length femur I 1.19–1.2 × carapace width.

##### Distribution.

Known from two caves in Bexar County, Texas (Fig. 59).

#### Tayshaneta valverdae

(Gertsch, 1974)

http://species-id.net/wiki/Tayshaneta_valverdae

**Figure 28.**
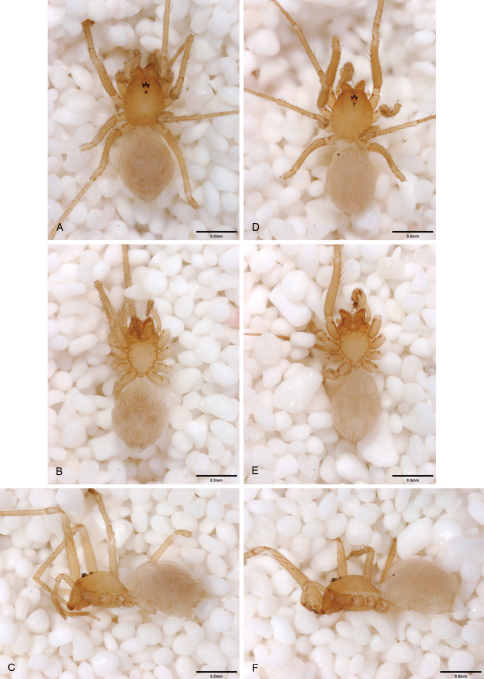
*Tayshaneta valverdae* sp. n., Oriente Milestone Molasses Bat Cave, Val Verde County, Texas (AMNH), habitus. **A** *Tayshaneta valverdae* male holotype, dorsal **B** *Tayshaneta valverdae* male holotype, ventral **C** *Tayshaneta valverdae* male holotype, lateral.

Figs 28A–F 49A–F 54C 60

Leptoneta valverdae Gertsch 1974: 173.
Neoleptoneta valverdae (Gertsch, 1974): Brignoli 1977: 216; Platnick 1986: 10.
Tayshaneta valverdae (Gertsch 1974): Ledford et al. 2011.

##### Type data.

Male holotype from Oriente Milestone Molasses Bat Cave, 20 miles NE of Del Rio, Val Verde County, Texas, 25-January-1964, J. Reddell, McKenzie, Porter, 29.56N, 100.77W, (AMNH).

##### Other material examined.

**USA:** Texas: **Bandera County:** Melanie’s Cave, Hill Country State Natural Area, 23-July-2000, J. Reddell, M. Reyes, 29.63N, 99.18W, 1 ♂, 5 ♀, 2 juvs, (TMM); Harvestman Cave, Hill Country State Natural Area, 24-July-2000, J. Reddell, M. Reyes, 29.63N, 99.18W, 1 ♂, 2 ♀, 2 juvs, (TMM); Love Creek Ranch, 10.5 miles W. Medina, 6-October-1996, A. Grubbs, 29.79N, 99.42W, 1 ♂, 1 ♀, (TMM); **Uvalde County:** Big Fucking Snake Cave, 8-June-1985, A. Grubbs, AC, RW, 29.43N, 99.65W, 1 ♂, 1 ♀, (AMNH); Marneldo Ranch, 18-April-1997, A. Grubbs, 29.50N, 99.61W, 1 ♂, (TMM); **Val Verde County:** Oriente Milestone Molasses Bat Cave, 20 miles NE of Del Rio, 25-January-1964, 1 ♂, (AMNH).

##### Diagnosis.

*Tayshaneta valverdae* may be separated from all other *Tayshaneta* species, except *Tayshaneta emeraldae*, *Tayshaneta fawcetti*, *Tayshaneta grubbsi* and *Tayshaneta vidrio* by having the male palpal tarsus divided apically (TS, Fig. 31D) and by having a mesoapically positioned ventral sclerite on the palpal bulb (VS, Fig. 49E). Separated from *Tayshaneta emeraldae*, *Tayshaneta fawcetti*, *Tayshaneta grubbsi* and *Tayshaneta vidrio* by the unique shape of the embolus with a prominent basal tooth (Fig. 49D).

##### Description.

Complete description in Gertsch (1974: 173). Habitus of male and female in Figs 28A–F, scanning electron micrographs of male palp in Figs 49A–F and female genitalia in Fig. 54C.

##### Distribution.

Known from caves and surface localities in Bandera, Uvalde and Val Verde Counties, Texas (Fig. 60).

#### Tayshaneta vidrio sp. n.

urn:lsid:zoobank.org:act:AFE6D515-6AC6-4C36-907B-5C513DAFA711

http://species-id.net/wiki/Tayshaneta_vidrio

**Figure 29.**
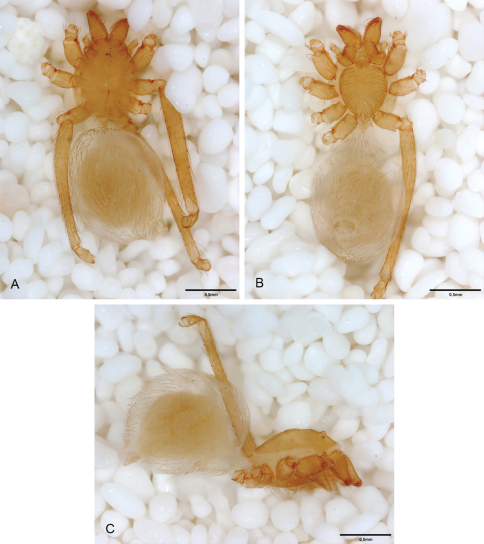
*Tayshaneta vidrio* sp. n., 400 Foot Cave, Brewster County, Texas (AMNH), habitus. **A** *Tayshaneta vidrio* male holotype, dorsal **B** *Tayshaneta vidrio* male holotype, ventral **C** *Tayshaneta vidrio* male holotype, lateral.

Figs 29A–C 31E 50A–F 54D 60

##### Type data.

Male holotype from 400 Foot Cave, Glass Mountains, Brewster County, Texas, 30.38N, 103.15W, (AMNH).

##### Etymology.

This species name is derived from the Spanish name for the Glass Mountains “Sierra del Vidrio” in West Texas. The name is to be treated as a noun in apposition.

##### Diagnosis.

*Tayshaneta vidrio* may be separated from all other *Tayshaneta* species, except *Tayshaneta emeraldae*, *Tayshaneta fawcetti*, *Tayshaneta grubbsi* and *Tayshaneta valverdae* by having the male palpal tarsus divided apically (Fig. 31D) and by having a mesoapically positioned ventral sclerite on the palpal bulb (VS, Fig. 50E). Separated from *Tayshaneta emeraldae*, *Tayshaneta fawcetti*, *Tayshaneta grubbsi* and *Tayshaneta valverdae* by having an oval embolus that is smooth along its margins and a ventral sclerite with a distinct apical division (VS, Fig. 50D–F).

##### Description.

**Male** (holotype). Body length 1.98, carapace 0.80 long, 0.65 wide, length 1.22× width. Carapace orange-yellow, sparsely setose; eyes reduced, surrounded by faint dark markings (Figs 29A–C). Legs elongate and thin, femur I 1.84× carapace length, covered in fine setae and with few scattered spines. Palpal tarsus divided apically (Fig. 31E); retrolateral tibial spine straight, sculptured throughout, length 0.51× tarsus length. Bulb suboval, length 1.70× width; embolus oval, curved at its base, smooth along margins (E, Fig. 50D), length 2.5× width. Abdomen pale yellow, without pattern, 1.18 long, 0.92 wide, covered in fine setae.

**Female** (400ft. Cave).Body length 1.49, carapace 0.63 long, 0.50 wide, length 1.25× width. Pigmentation, setation and eyes same as for male. Legs elongate and thin, femur I 1.57× carapace length, covered in fine setae and with few scattered spines.Atrium oval, length 1.5× width, spermathecae with twisted stalks and elongate heads (Fig. 54D). Abdomen pale yellow, 0.85 long, 0.70 wide, covered in fine setae.

**Variation** **(*n* = 2).** Total length 1.49–1.81; carapace length 1.10–1.25 × carapace width; length femur I 1.50–1.57 × carapace width.

##### Distribution.

Known only from 400 foot Cave, Brewster County, Texas (Fig. 60).

#### Tayshaneta whitei sp. n.

urn:lsid:zoobank.org:act:A0D6F247-E6F5-4DBE-A2EF-4C5354D13AC2

http://species-id.net/wiki/Tayshaneta_whitei

Figs 1D 30A–F 51A–F 54E 59

##### Type.

Male holotype from Lithic Ridge Cave, Government Canyon State Natural Area, Bexar County, Texas, 6-November-2002, Engelhard, J. Krejca, 29.56N, 98.74W, (AMNH).

##### Etymology.

This species is named in honor of Kemble White, fellow caver, geologist and collector of many *Tayshneta* species in Texas.

##### Other material examined.

**USA:** Texas: **Bexar County:** Caracol Creek Coon Cave, 15-June-1993, J. Loftin, J. Reddell, M. Reyes, G. Veni, 29.45N, 98.71W, 1 juv., (TMM); Caracol Creek Coon Cave, 10-March-2005, P. Paquin, 29.45N, 98.71W, 1 ♂, 2 ♀, 1 juv., (CASC); Cave site #801, West of Helotes, November-1999, K. White, 1 ♂, 1 ♀, 1 juv., (TMM); Lithic Ridge Cave, Government Canyon State Natural Area, 1-October-1994, Palit, Atkinson, 29.56N, 98.74W, 1 juv., (TMM); Lithic Ridge Cave, Government Canyon State Natural Area, 4-June-1995, G. Veni, 29.56N, 98.74W, 1 ♀, (TMM); Lithic Ridge Cave, Government Canyon State Natural Area, 6-November-2002, Englehard, J. Krejca, 29.56N, 98.74W, 2 ♂, 2 ♀, 1 juv., (TMM); Lithic Ridge Cave, Government Canyon State Natural Area, 12-November-2009, J. Ledford, M. Sanders, N. Lake, 29.56N, 98.74W, 2 ♂, 2 ♀, 1 juv., (TMM); **Medina County:** Medina Dam Cave, June-2010, K. McDermid, 1 juv., (TMM); Nisbet Cave, 4-March-2001, G. Veni, Waters, 29.53N, 98.91W, 1 ♂, (TMM).

##### Diagnosis.

*Tayshaneta whitei* may be separated from all *Tayshaneta* species, except *Tayshaneta bullis* and *Tayshaneta microps*, by having a combination of males with an elongate retrolateral tibial spine, more than 0.5× length of the palpal tarsus and lacking a ventral sclerite (Figs 51B, E). Separated from *Tayshaneta bullis* and *Tayshaneta microps* by the unique shape of the embolus (E, Fig. 51D) and the distinctive retrlolateral sclerite (RS, Figs 51D–E).

##### Description.

**Figure 30.**
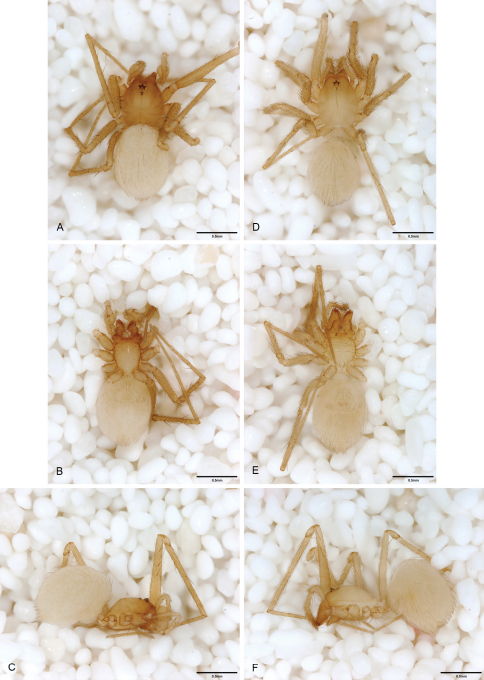
*Tayshaneta whitei* sp. n., Lithic Ridge Cave, Bexar County, Texas (CASC), habitus. **A** *Tayshaneta whitei* male, dorsal **B** *Tayshaneta whitei* male, ventral **C** *Tayshaneta whitei* male, lateral **D** *Tayshaneta whitei* female, dorsal **E** *Tayshaneta whitei* female, ventral **F** *Tayshaneta whitei* female, lateral.

**Male** (holotype). Body length 1.52, carapace 0.61 long, 0.50 wide, length 1.21× width. Carapace brown, sparsely setose; eyes surrounded by dark markings (Figs 30A–C). Legs elongate and thin, femur I 1.61× carapace length, covered in fine setae and with few scattered spines. Palpal tarsus undivided, tapering apically; retrolateral tibial spine straight, sculptured throughout, length 0.51× tarsus length. Bulb suboval, length 1.71× width; embolus subquadrate, with small basal tooth (E, Fig. 51D–F), length 1.6× width. Abdomen white, without pattern, 0.90 long, 0.67 wide, covered in fine setae.

**Variation** **(*n* = 4).** Total length 1.52–1.70; carapace length 1.16–1.21 × carapace width; length femur I 1.45–1.92 × carapace width.

**Female** (Lithic Ridge Cave).Body length 1.49, carapace 0.61 long, 0.49 wide, length 1.25× width. Carapace light brown, sparsely setose; eyes surrounded by dark markings (Figs 30D–F). Legs elongate and thin, femur I 1.58× carapace length, covered in fine setae and with few scattered spines.Atrium oval, length 0.5× width, spermathecae with twisted stalks and large, circular heads (Fig. 54E).Abdomen light brown, 0.90 long, 0.60 wide, covered in fine setae.

**Variation** **(*n* = 3).** Total length 1.41–1.80; carapace length 1.18–1.28 × carapace width; length femur I 1.33–1.58 × carapace width.

##### Natural History.

Several individuals of *Tayshaneta whitei* were collected under loose stones near the bases of walls in Lithic Ridge Cave, Bexar County Texas.

##### Distribution.

Known from caves in Bexar and Medina Counties, Texas (Fig. 59).

**Figure 31.**
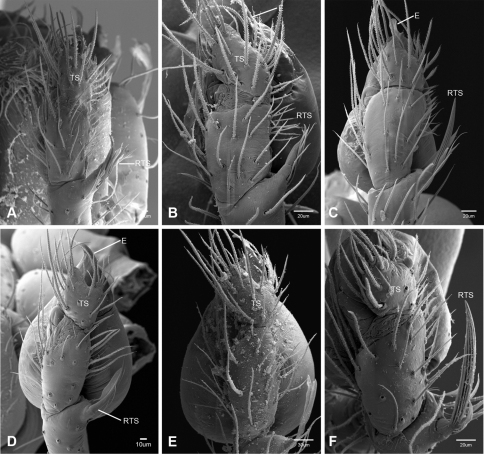
Morphology of *Tayshaneta* right male palpi in dorsal view, showing differences in tarsal shape. **A** *Tayshaneta paraconcinna* (Cokendolpher & Reddell, 2001), Scoot Over Cave, Williamson County, Texas **B** *Tayshaneta devia* (Gertsch, 1974), Stovepipe Cave, Travis County, Texas **C** *Tayshaneta microps* (Gertsch, 1974), Bexar County, Texas **D** *Tayshaneta fawcetti* sp. n., Fawcett’s Cave, Val Verde County, Texas **E** *Tayshaneta vidrio*, sp. n., 400 foot Cave, Brewster County (RTS damaged), Texas **F** *Tayshaneta madla* sp. n., Madla’s Drop Cave, Bexar County, Texas.

**Figure 32.**
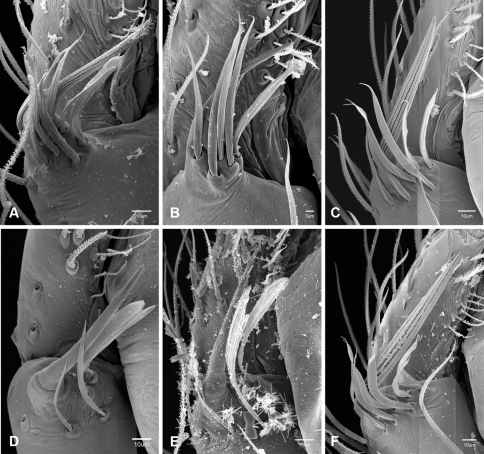
General morphology of *Tayshaneta* male palpi, showing differences in retrolateral tibial spine. **A** *Tayshaneta paraconcinna* (Cokendolpher & Reddell, 2001), Scoot Over Cave, Williamson County, Texas **B** *Tayshaneta devia* (Gertsch, 1974), Stovepipe Cave, Travis County, Texas **C** *Tayshaneta microps* (Gertsch, 1974), Bexar County, Texas **D** *Tayshaneta fawcetti* sp. n., Fawcett’s Cave, Val Verde County, Texas **E** *Tayshaneta grubbsi*, sp. n., Litterbarrel Cave, Val Verde County, Texas **F** *Tayshaneta madla* sp. n., Cave Number 189, Bexar County, Texas.

**Figure 33.**
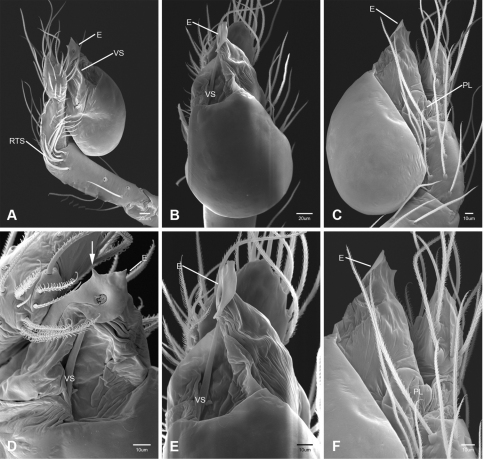
*Tayshaneta anopica* (Gertsch, 1974), Cobb’s Cave, Williamson County, Texas (CASC), male right palp. **A** Retrolateral **B** Ventral **C** Prolateral **D** Retrolateral, embolus **E** Ventroapical **F** Proapical.

**Figure 34.**
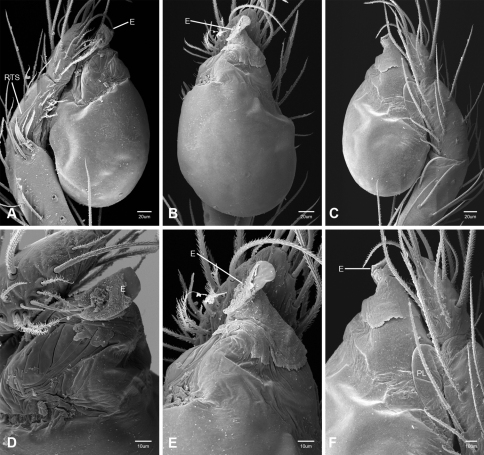
*Tayshaneta archambaulti* sp. n., Grapevine Ranch Cave, Hays County, Texas (CASC), male right palp. **A** Retrolateral **B** Ventral **C** Prolateral **D** Retrolateral, embolus **E** Ventroapical **F** Proapical.

**Figure 35.**
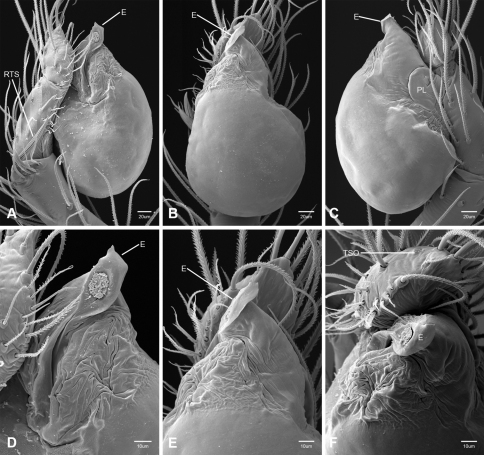
*Tayshaneta bullis* (Cokendolpher, 2004), Up the Creek Cave, Camp Bullis, Bexar County, Texas (TMM), male right palp. **A** Retrolateral **B** Ventral **C** Prolateral **D** Retrolateral, embolus **E** Ventroapical **F** Apical.

**Figure 36.**
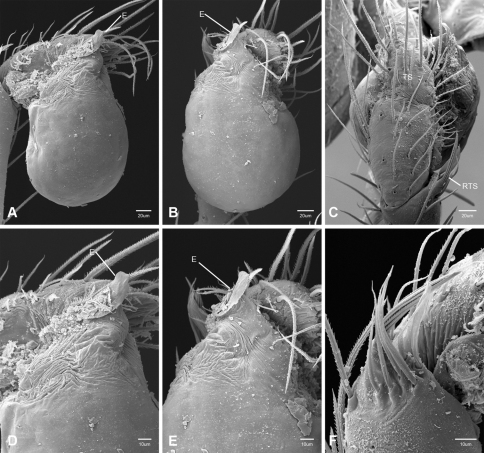
*Tayshaneta coeca* (Chamberlin and Ivie, 1942), Heidrich’s Cave, Comal County, Texas (AMNH), male right palp. **A** Retrolateral **B** Ventral **C** Tarsus, dorsal **D** Retrolateral, embolus **E** Ventroapical **F** Retrolateral tibial spine.

**Figure 37.**
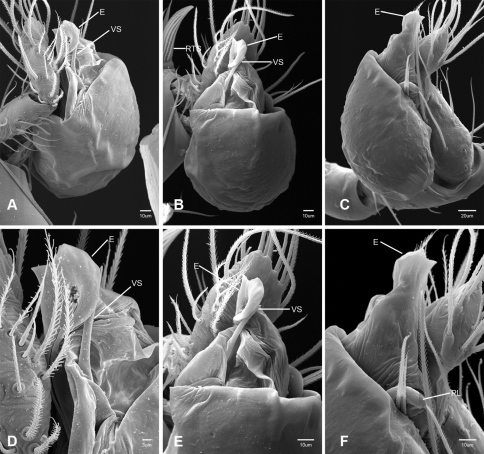
*Tayshaneta concinna* (Gertsch, 1974), Lost Gold Cave, Travis County, Texas (CASC), male right palp. **A** Retrolateral **B** Ventral **C** Prolateral **D** Retrolateral, embolus **E** Ventroapical **F** Proapical.

**Figure 38.**
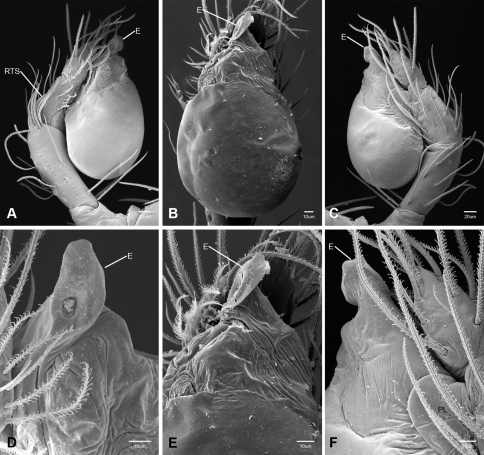
*Tayshaneta devia* (Gertsch, 1974), MacDonald Cave, Travis County, Texas (CASC), male right palp. **A** Retrolateral **B** Ventral **C** Prolateral **D** Retrolateral, embolus **E** Ventroapical **F** Proapical.

**Figure 39.**
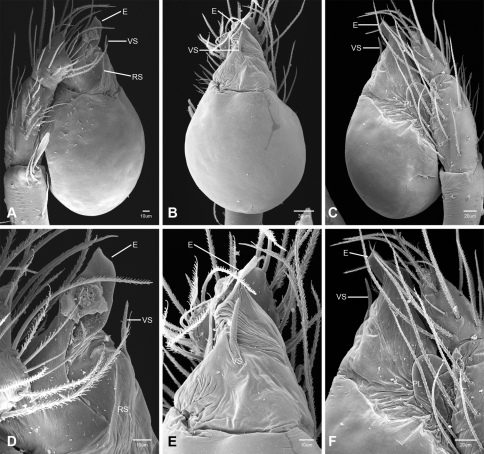
*Tayshaneta emeraldae* sp. n., Emerald Sink, Val Verde County, Texas (AMNH), male right palp. **A** Retrolateral **B** Ventral **C** Prolateral **D** Retrolateral, embolus **E** Ventroapical **F** Proapical.

**Figure 40.**
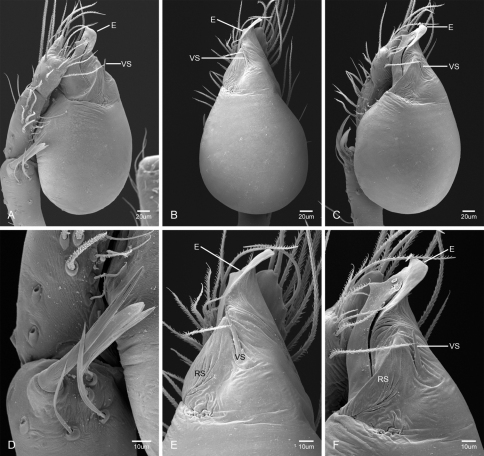
*Tayshaneta fawcetti* sp. n., Fawcett’s Cave, Val Verde County, Texas (CASC), male right palp. **A** Retrolateral **B** Ventral **C** Ventrolateral **D** Retrolateral tibial spine **E** Ventroapical **F** Retrolateral, embolus.

**Figure 41.**
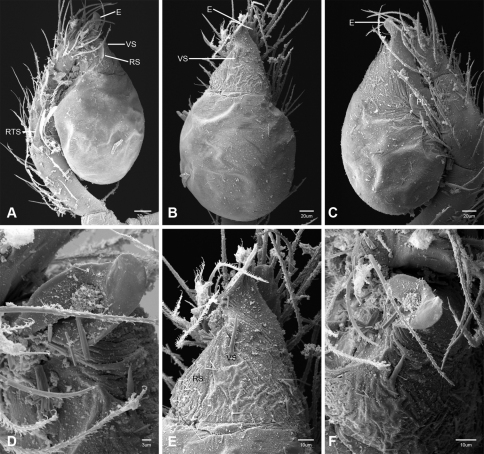
*Tayshaneta grubbsi* sp. n., Litterbarrel Cave, Val Verde County, Texas (AMNH), male right palp. **A** Retrolateral **B** Ventral **C** Prolateral **D** Retrolateral, embolus **E** Ventroapical **F** Apical.

**Figure 42.**
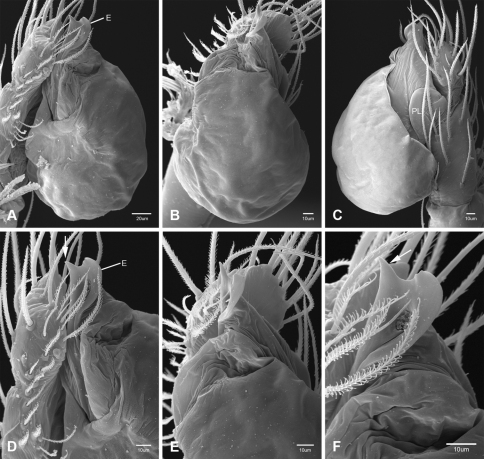
*Tayshaneta madla* sp. n., Madla’s Cave, Bexar County, Texas (CASC), male right palp. **A** Retrolateral **B** Ventral **C** Prolateral **D** Retrolateral, embolus **E** Ventroapical **F** Embolus.

**Figure 43.**
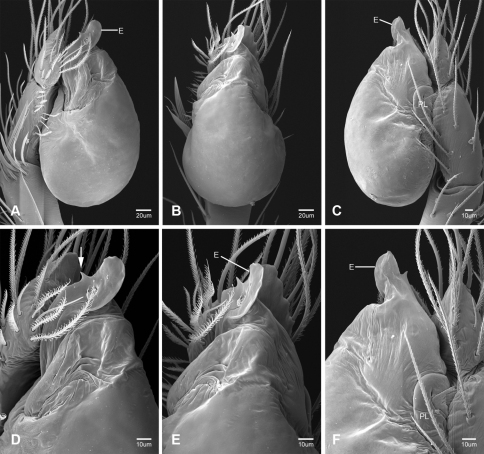
*Tayshaneta microps* (Gertsch, 1974), Government Canyon Bat Cave, Bexar County, Texas (CASC), male right palp. **A** Retrolateral **B** Ventral **C** Prolateral **D** Retrolateral, embolus **E** Ventroapical **F** Proapical.

**Figure 44.**
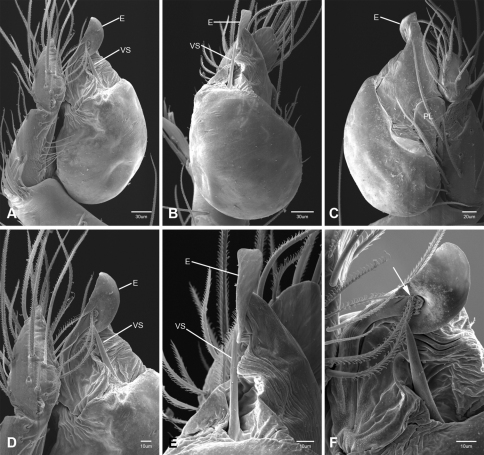
*Tayshaneta myopica* (Gertsch, 1974), Tooth Cave, Travis County, Texas (CASC), male right palp. **A** Retrolateral **B** Ventral **C** Prolateral **D** Retrolateral, embolus **E** Ventroapical **F** Embolus.

**Figure 45.**
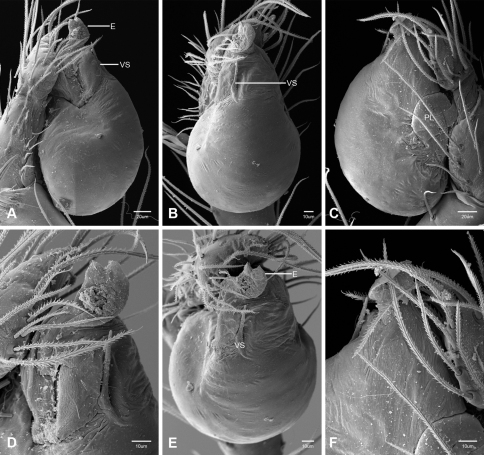
*Tayshaneta oconnorae* sp. n., Fern Cave, Hays County, Texas (TMM), male right palp. **A** Retrolateral **B** Ventral **C** Prolateral **D** Retrolateral, embolus **E** Ventroapical **F** Proapical.

**Figure 46.**
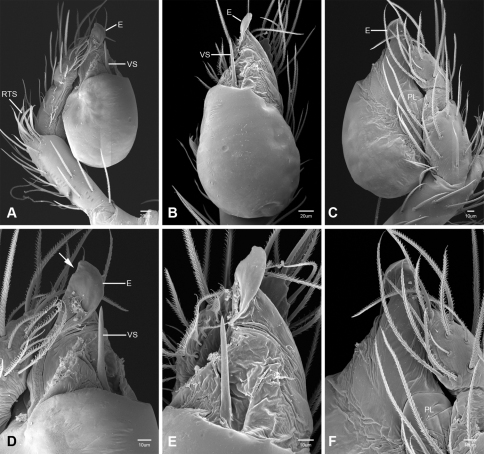
*Tayshaneta paraconcinna* (Cokendolpher & Redell, 2001), Fern Cave, Hays County, Texas (TMM), male right palp. **A** Retrolateral **B** Ventral **C** Prolateral **D** Retrolateral, embolus **E** Ventroapical **F** Proapical.

**Figure 47.**
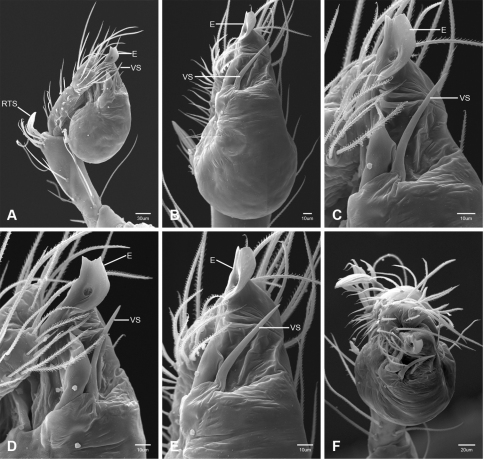
*Tayshaneta sandersi* sp. n., Whirlpool Cave, Travis County, Texas (CASC), male right palp. **A** Retrolateral **B** Ventral **C** Retroventral **D** Retrolateral, embolus **E** Ventroapical **F** Apical.

**Figure 48.**
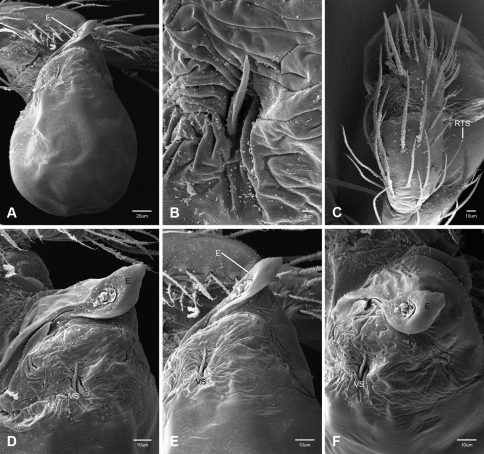
*Tayshaneta sprousei* sp. n., Constant Sorrow Cave, Camp Bullis, Bexar County, Texas (TMM), male right palp. **A** Ventral **B** Ventral sclerite **C** Tarsus, dorsal **D** Retrolateral, embolus **E** Ventroapical **F** Apical.

**Figure 49.**
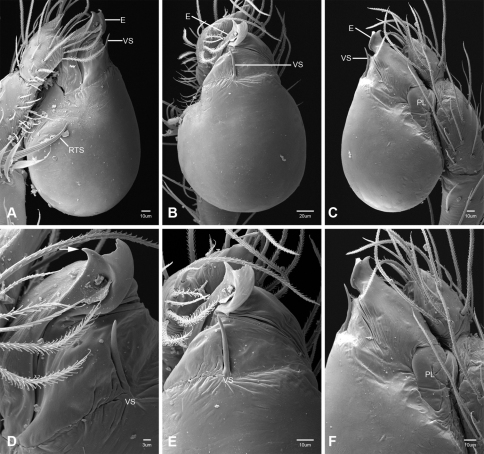
*Tayshaneta valverdae* (Gertsch, 1974), Oriente Milestone Molasses Bat Cave, Val Verde County, Texas (AMNH), male right palp. **A** Retrolateral **B** Ventral **C** Prolateral **D** Retrolateral, embolus **E** Ventroapical **F** Proapical.

**Figure 50.**
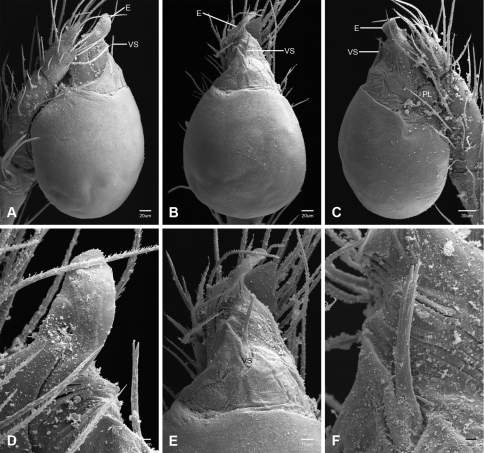
*Tayshaneta vidrio* sp. n., 400 foot Cave, Brewster County, Texas (AMNH), male right palp. **A** Retrolateral **B** Ventral **C** Prolateral **D** Embolus **E** Ventroapical **F** Ventral sclerite.

**Figure 51.**
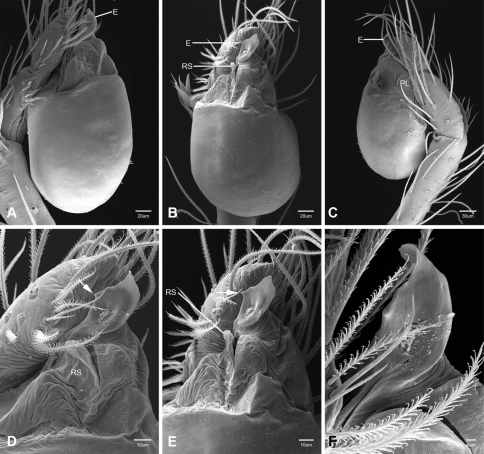
*Tayshaneta whitei* sp. n., Lithic Ridge Cave, Bexar County, Texas (CASC), male right palp. **A** Retrolateral **B** Ventral **C** Prolateral **D** Retrolateral, embolus **E** Ventroapical **F** Embolus.

**Figure 52.**
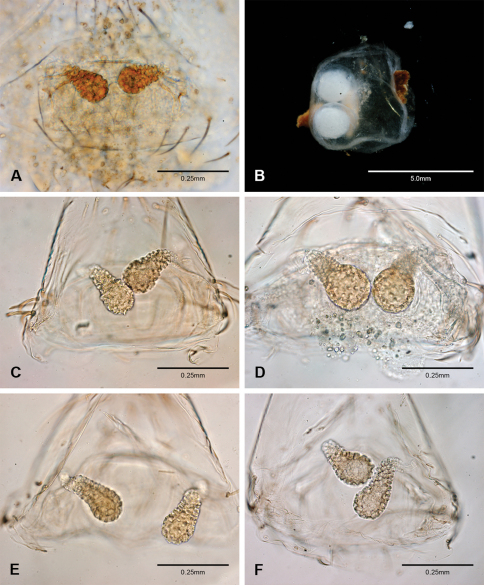
Female genitalia for*Tayshaneta* species. **A** *Tayshaneta anopica* (Gertsch, 1974), Corn Cobb’s Cave, Williamson County, Texas **B** Egg-sac of *Tayshaneta anopica* (Gertsch, 1974), Corn Cobb’s Cave, Williamson County, Texas **C** *Tayshaneta archambaulti* sp. n., Grapevine Cave, Hays County, Texas **D** *Tayshaneta bullis* (Cokendolpher, 2004), Up the Creek Cave, Bexar County, Texas **E** *Tayshaneta coeca* (Chamberlin and Ivie, 1942), Natural Bridge Caverns, Hays County, Texas **F** *Tayshaneta concinna* (Gertsch, 1974), Lost Gold Cave, Travis County, Texas.

**Figure 53.**
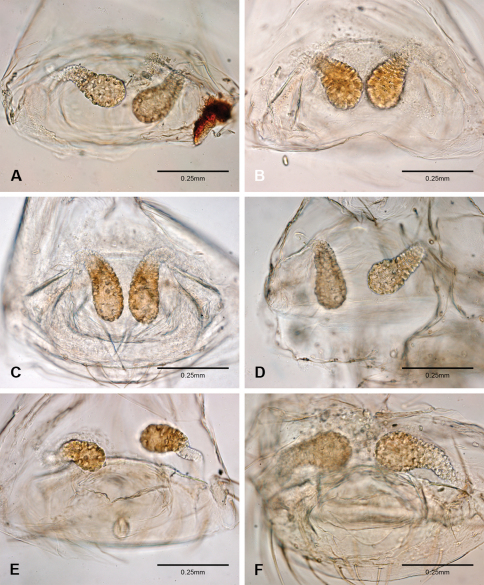
Female genitalia for*Tayshaneta* species. **A** *Tayshaneta devia* (Gertsch, 1974), MacDonald Cave, Travis County, Texas **B** *Tayshaneta emeraldae* sp. n., Emerald Sink, Val Verde County, Texas **C** *Tayshaneta fawcetti* sp. n., Fawcett’s Cave, Val Verde County, Texas **D** *Tayshaneta madla* sp. n., Madla’s Cave, Bexar County, Texas **E** *Tayshaneta microps* (Gertsch, 1974), Government Canyon Bat Cave, Bexar County, Texas **F** *Tayshaneta myopica* (Gertsch, 1974), Tooth Cave, Travis County, Texas.

**Figure 54.**
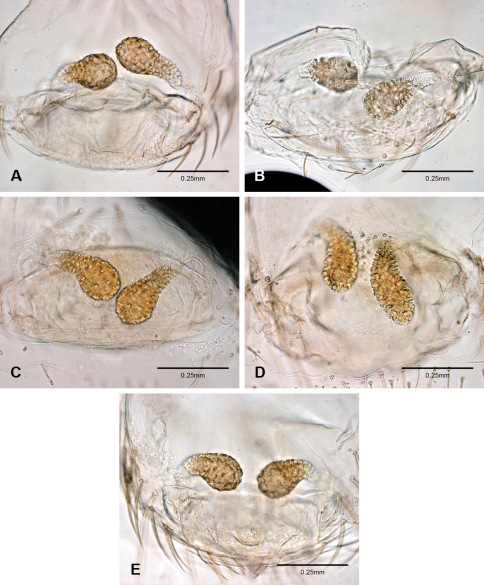
Female genitalia for*Tayshaneta* species. **A** *Tayshaneta paraconcinna* (Cokendolpher & Reddell, 2001), Figure 8 Cave, Fort Hood, Bell County, Texas **B** *Tayshaneta sandersi* sp. n., District Park Cave, Travis County, Texas **C** *Tayshaneta valverdae* (Gertsch, 1974), Love Creek Ranch, Bandera County, Texas **D** *Tayshaneta vidrio* sp. n., 400 foot Cave, Brewster County, Texas **E** *Tayshaneta whitei* sp. n., Lithic Ridge Cave, Bexar County, Texas.

**Figure 55.**
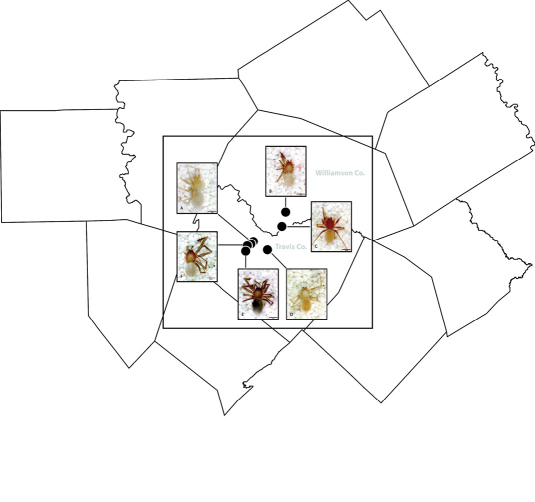
Distribution map showing morphotypes within *Tayshaneta myopica* (Gertsch, 1974). **A** Tooth Cave **B** Goat Cave **C** McNeil Bat Cave **D** Jester Estates Caves **E** Steiner Telephone Pole Cave **F** Geode Cave.

**Figure 56.**
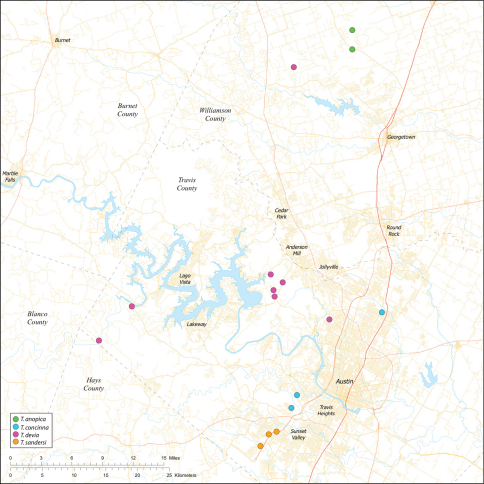
Distribution map for *Tayshaneta anopica* (Gertsch, 1974), *Tayshaneta concinna* (Gertsch, 1974), *Tayshaneta sandersi* sp. n. and *Tayshaneta devia* (Gertsch, 1974).

**Figure 57.**
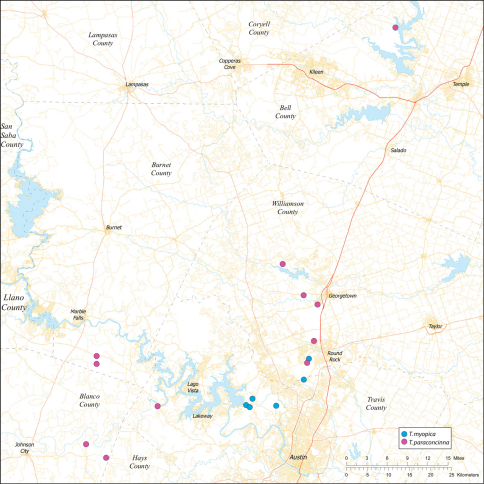
Distribution map for *Tayshaneta myopica* (Gertsch, 1974) and *Tayshaneta paraconcinna* (Cokendolpher & Reddell, 2001).

**Figure 58.**
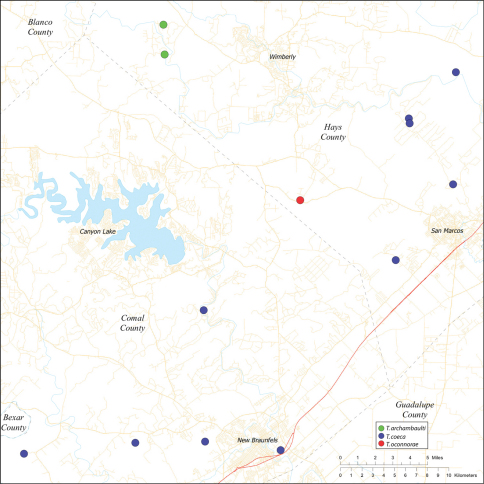
Distribution map for *Tayshaneta archambaulti* sp. n. *Tayshaneta coeca* (Chamberlin and Ivie, 1942) and *Tayshaneta oconnorae* sp. n.

**Figure 59.**
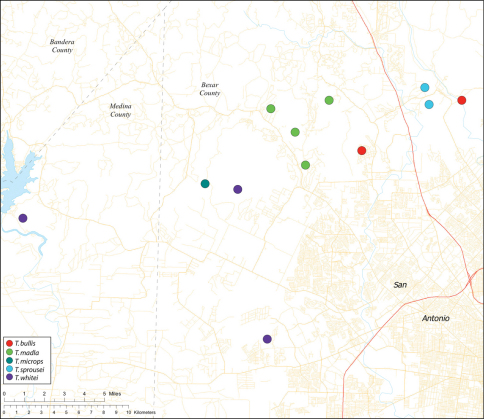
Distribution map for *Tayshaneta madla* sp. n., *Tayshaneta bullis* (Cokendolpher, 2004), *Tayshaneta microps* (Gertsch, 1974), *Tayshaneta sprousei* sp. n. and *Tayshaneta whitei* sp. n.

**Figure 60.**
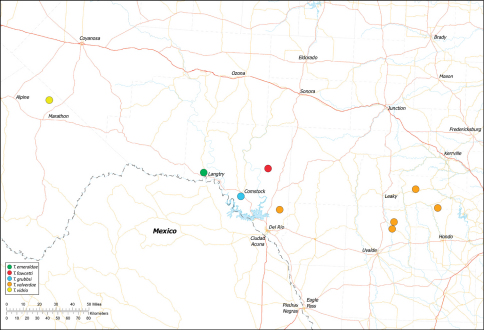
Distribution map for *Tayshaneta emeraldae* sp. n., *Tayshaneta fawcetti* sp. n., *Tayshaneta grubbsi* sp. n., *Tayshaneta valverdae* (Gertsch, 1974) and *Tayshaneta vidrio* sp. n.

**Figure 61.**
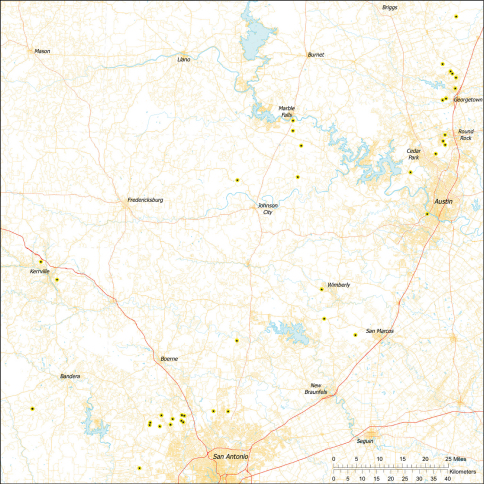
Distribution map for undetermined *Tayshaneta* species.

**Figure 62.**
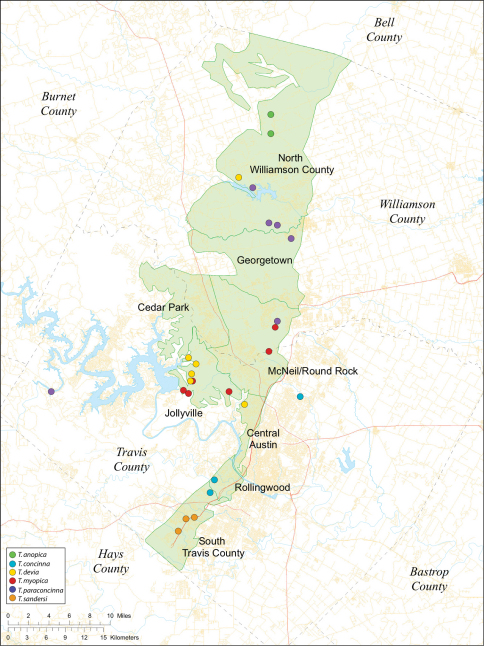
Distribution of *Tayshaneta* species in Travis and Williamson Counties superimposed on Karst Faunal Regions (KFR’s).

**Figure 63.**
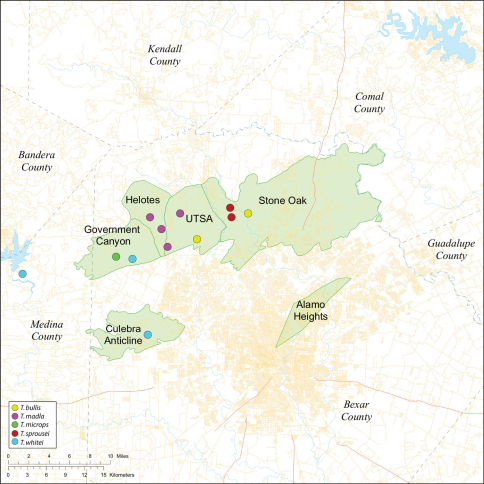
Distribution of *Tayshaneta* species in Bexar County superimposed on Karst Faunal Regions.

## Supplementary Material

XML Treatment for Tayshaneta
